# Paclitaxel-Containing Extract Exerts Anti-Cancer Activity through Oral Administration in A549-Xenografted BALB/C Nude Mice: Synergistic Effect between Paclitaxel and Flavonoids or Lignoids

**DOI:** 10.1155/2022/3648175

**Published:** 2022-04-25

**Authors:** Dake Cai, Jing Jin, Huichang Bi, Guoping Zhong, Minhua Zhou, Jianfen Guo, Yike Cai, Miaoyin Liang, Qiong Gu, Zixuan Hu, Yijing Lai, Zi Dai, Lingjie Li, Yuxing Chen, Haili Gao, Min Huang

**Affiliations:** ^1^Institute of Clinical Pharmacology, School of Pharmaceutical Science, Sun Yat-sen University, Guangzhou 510006, China; ^2^The Fifth Clinical College of Guangzhou University of Chinese Medicine, Guangzhou, Guangdong 510095, China; ^3^Department of Pharmacy, Guangdong Provincial Hospital of Integrated Traditional Chinese and Western Medicine, Guangdong, Foshan 528200, China; ^4^Center for Certification and Evaluation, Guangdong Drug Administration, Guangzhou, Guangdong 510080, China; ^5^Research Center for Drug Discovery, School of Pharmaceutical Science, Sun Yat-sen University, Guangzhou 510006, China

## Abstract

Taxus *yunnanensis* is a paclitaxel-containing herb with traditional usage in cancer treatment, and its extract possesses great oral bioavailability of paclitaxel. However, it is elusive whether paclitaxel-containing extract (HDS-1) can exert anti-tumor effect through oral administration and how other components contribute to its efficacy. Therefore, we investigate the oral-route anti-tumor effect of HDS-1 in A549-bearing mice. HDS-1-derived flavonoids (HDS-2) and lignoids (HDS-3) are hypothesized to contribute to HDS-1's efficacy, and their effects of enhancing enterocytic absorption and cytotoxicity of paclitaxel are validated in 2 permeability experiments and apoptosis-related assay, respectively. *In vivo*, A549 growth is significantly inhibited by 86.1 ± 12.94% (*P* < 0.01) at 600 mg/kg of HDS-1 and 65.7 ± 38.71% (*P* < 0.01) at 200 mg/kg. HDS-2 and HDS-3 significantly reduce the efflux ratio of paclitaxel to 2.33 and 3.70, respectively, in Caco-2 permeability experiment and reduce paclitaxel reflux in MDCK-MDR1 experiment. Furthermore, HDS-2 and HDS-3 potentiated paclitaxel-induced cytotoxicity by 19.1–22.45% (*P* < 0.05) and 10.52–18.03% (*P* < 0.05), respectively, inhibited the expression of cyclinB1, Bcl-2, and pMCL-1, and increased the percentage of necrosis cell in the condition of paclitaxel exposure. Conclusively, paclitaxel-containing extracts exert anti-cancer effects through oral administration, and flavonoid and lignoids contribute to its anti-cancer effect through simultaneously improving enterocytic absorption of paclitaxel and the cytotoxic effect of paclitaxel.

## 1. Introduction

Taxus *yunnanensis* Cheng et L.K. Fu is a traditional Chinese medicine, which is widely distributed in the southern regions of China [1] and is traditionally used for treating diabetes, cancer, etc [2]. It is recorded as “Hongdoushan” [3] in folk and local pharmacopeia in some regions of China, and it is abbreviated as HDS in our research. Currently, Taxus *yunnanensis* Cheng et L.K. Fu (HDS-1) serves as a critical anti-cancer herbal component in Chinese medicine decoction in clinics, and it's commonly prescribed for the elderly with non-small cell lung cancer [4]. Paclitaxel is commonly recognized to be an active component of HDS for treating cancer since it was isolated from Taxus and proved to be a potent anti-cancer agent with special microtubule-binding activity [5, 6]. Recently, it has proved that paclitaxel-containing extract (HDS-1) achieved greater oral bioavailability of paclitaxel than that of pure paclitaxel through oral administration [7], even though it was conventional awareness that paclitaxel is hardly absorbed in the traditional usage due to its poor oral bioavailability. It is supportive to elucidate rationality in the current usage of Taxus extract (HDS) for anti-cancer treatment orally, and this phenomenon is called “pharmacokinetic synergy.” However, it is still elusive whether oral administration of Taxus extract (HDS) can exert anti-cancer activity and how other coexisting components contribute to the anti-cancer effect of HDS. Therefore, A549-bearing nude mice are employed to validate the anti-cancer effect of HDS in oral route, and *in vitro* permeability assay (Caco-2 bidirectional model) and cytotoxicity assay (MTT) are applied to validate the contributive effect of other coexisting components in HDS.

HDS extract is comprised of various types of components including taxane, flavonoids, and lignoids. Taxane diterpenoids are characteristic compounds of the Taxus plant, and paclitaxel is one of the taxane-diterpenoid compounds with specific micro-tubulin binding effect. Taxus extract HDS-1 contains 2.5% paclitaxel, which is higher than that in reported research [8]. To some extent, high-content paclitaxel can guarantee its anti-cancer effect through oral administration. Moreover, other components may contribute to paclitaxel's anti-cancer through affecting paclitaxel's oral absorption [9, 10]. Emerging research revealed that flavonoids or lignoids are candidate bioenhancers for paclitaxel [11–13] due to their regulation on the agent's transporter [14–17] in enterocytes. Furthermore, flavonoids and lignoids possess various pharmacological properties including anti-oxidation [18], anti-inflammation [19], and anti-cancer [20, 21], indicating that they may synergy with paclitaxel as cytotoxic enhancers. Especially, some flavonoids are proved to exert synergistic effect for paclitaxel-caused cell death and proliferation-inhibition in A549 or paclitaxel-resistant A549 cell line [22, 23]. Therefore, in our experiment, flavonoids and lignoids are isolated from the crude extract. Their synergistic effect with paclitaxel (bioenhancer or cytotoxic enhancer) is validated in the Caco-2 bidirectional transport experiment and MTT assay, respectively.

The Caco-2 bidirectional transport experiment is extensively used as a human colon adenocarcinoma model for evaluating the permeability of drug candidates and their interaction with transporters *in vitro*, and it is recommended as a convincing model in Biopharmaceutics Classification System of FDA [24]. Caco-2 cells are cultured for 21 days and then are differentiated into a tidy barrier in transwell; it is well mimicked for the enterocytic absorption barrier of paclitaxel due to its high expression of P-glycoprotein (P-gp), which is responsible for refluxing paclitaxel to the gut lumen. Therefore, the Caco-2 permeability model is suitable to evaluate the bioenhancer for paclitaxel in specific tissues of oral route, and it will, to some extent, explain the contributive effect of flavonoids and lignoids on enterocyte absorption of paclitaxel in the oral administration of HDS. Moreover, Madin–Darby canine kidney–multidrug resistance protein 1 (MDCK–MDR1) is a canine kidney cell line expressing transfected human P-gp (coded by gene MDR1), and it is applied to validate the inhibitory effect of HDS-2 or HDS-3 on P-gp-induced reflux of paclitaxel. Additionally, paclitaxel exerts its anti-cancer effect through a dominant cytotoxic effect including micro-tubulin binding, cell cycle arresting, mitochondria damaging, and cell apoptosis [25–27]. The MTT assay is a method for evaluating cytotoxicity *in vitro*, and it can reflect the integrated cytotoxicity from single agent or combinative treatment *in vitro*. Therefore, it is employed as a measurement for exploring the synergistic effect between candidate components (flavonoids or lignoids) and paclitaxel. Furthermore, apoptosis-related BCL-2 family protein expressions and PI and Annexin V staining experiment are applied to evaluate the synergistic effect between paclitaxel and flavonoids or lignoids with regard to the mechanism underlying cytotoxicity. Fortunately, the inherent synergistic effect is validated between paclitaxel and flavonoid or lignoids, and our finding highlights that HDS is a prospective oral-administrated remedy for non-small lung cancer.

## 2. Materials and Methods

### 2.1. Reagents

Barks and leaves of Taxus *yunnanensis* Cheng et L.K. Fu are provided by Zhongda Nanyao Co Ltd. Paclitaxel (Batch No. SH1102-1121 purity >98%), docetaxel (Batch No. SH1102-1255, internal standard, purity >95%), and Wuweiz alcohol B (WAB, known as schisandrol B, Batch No. SH1102-1189 purity >98%) were obtained from Shanghai Winherb Medical S & *T* Development Co Ltd. Verapamil (Batch No. V4629-5 G, purity >99.0%) was obtained from Sigma. DMEM (Dulbecco's Modified Eagle's medium) was obtained from HyClone. FBS, HBSS, and PBS were obtained from GIBCO. Penicillin and streptomycin were obtained from North China pharmaceutical Group Corporation. BSA was obtained from Sangon Biotech (Shanghai) Co.Ltd. Methanol and tert-butyl methyl ether were obtained from MERCK. Quercetin (purity >95%) and taxanes are provided by Dr. Gu Qiong, and taxane include 10-Deacetyl Baccatin III (10-DAB III), Baccatin III, 7- Xylosyl-10-Deacetyl Cephalomannine, 7-Xylosyl-10-Deacetyl Taxol, Taxinine *M*, 7-Xylosyl-10-Deacetyl Taxol C, 10-Deacetyl Taxol, 7- Xylosyl Taxol, Cephalomannine, 7-Epi-10-Deacetyl Taxol, Taxol C, 7-Epi Taxol, and Taxacin (their structure is identified by NMR, purity >85%).

### 2.2. Extraction, Isolation, and Analysis of the Non-Taxane Component

Dried and pulverized barks (2.5 kg) and leaves of Taxus *yunnanensis* Cheng et L.K. Fu were extracted using a backstreaming apparatus with 70% ethanol/water for 3 times (3 × 15 L). The ethanol/water extract was concentrated (4.2 L) and partitioned sequentially with petroleum ether (b.p. 61°C, 3 × 0.6 L) and dichloromethane (3 × 1.5 L); the dichloromethane fraction (HDS-1) was dried in the evaporator (2.2 g). Further, HDS-1 was loaded on Sephadex LH-20 column (18–111 *μ*m, 4.5 × 100 cm, flow rate 1 mL/min) using a single elution of methanol, resulting in 3 fractions (A1–A3 fractions) after monitoring by TLC (silicagel 60, CHCl3 : CH3OH 4 : 1). HDS-2 (flavonoids, 0.2 g) was accumulated from A2 and HDS-3 (lignoids, 0.1 g) was accumulated from A1, while A3 was identified as taxane (1.1 g) using HPLC described as follows:

The content of the total flavonoid was determined by HPLC-UV and UV as described previously [28]. Determination of paclitaxel, sciadopitysin, and matairesinol, and chromatographic fingerprint analysis of HDS-1, HDS-2, and HDS-3 were performed on Shimadzu LC-20 UFLC system (Shimadzu, Tokyo, Japan) equipped with a LC-20AD pump, a SIL-20A autosampler, a CTO-20A oven, and an SPD-M20 A detector. The separation and analysis of taxane and lignoids in HDS-1 and HDS-3 was performed on a C18 Column (250 mm × 2.1 mm, 5 *μ*m, Curosil-PFP, Phenomenex, USA) kept at 25°C. The mobile phases consisted of acetonitrile (A) and water (B) as the following gradient programs at 1.0 mL/min: 0–50 min (25–65% A), 50–60 min (65–100% A), 60–70 min (100% A), 70–75 min (100–25% A), and 75–90 min (25% B). The eluent was monitored at 190–600 nm wavelengths. The separation and analysis of flavonoids in HDS-1 and HDS-2 was performed on a C18 Column (250 mm × 2.1 mm, 5 *μ*m, Genimi, Phenomenex, USA) kept at 25°C. The mobile phases consisted of methanol (A) and water (B) as the following gradient programs at 1.0 mL/min: 0–30 min (30–60% A), 30–40 min (60–80% A), 40–60 min (80% A), 60–65 min (80–100% A), 65–75 min (100% B), 75–80 min (100–30% A), and 80–90 min (30%). The eluent was monitored at 190–600 nm wavelengths. The injection volume for all samples was 20 *μ*L.

### 2.3. Preparation of Dosing Solutions

For intraperitoneal injection (*I.P.*) administration, commercially available paclitaxel formulation was diluted 2- or 4-fold with saline to yield a solution for 10 mg/kg (PTX *I.P.*) experiment. The required injection volume was 10 mL/kg and was administered into the abdominal cavity.

For intragastric (*I.G.*) administration, the HDS-1 extract was dissolved in a mixture of ethanol and Cremophor EL (1 : 1, vol/vol) and further diluted 10- or 20-fold with saline to achieve a solution for the 600, 200 mg/kg experiments, respectively. Paclitaxel and WAB were dissolved in a mixture of ethanol and Cremophor EL (1 : 1, vol/vol) and further diluted 10-fold with saline to achieve a solution for 10 + 50 (PTX + WAB) mg/kg. Volumes of 10 mL/kg (body weight) of these solutions were administered by direct injection into the stomach using a blunt-ended needle inserted via the esophagus.

All administration solutions were prepared with reference to the method previously reported by Alex Sparreboom et al. [29].

### 2.4. Animal

BALB/c mice (13∼15 g) were provided by Shanghai SLAC Laboratory Animal Co Ltd, PRC. License for BALB/c is SCKX (LU) 2007–0005. All male animals were maintained under environmentally controlled conditions of 23∼25°C and 12 h light/12 h dark cycle. The animals received humane care in accordance with the Guide for the Care and Use of Laboratory Animals, published by the US National Institution of Health (NIH Publication, revised in 1985). The experimental procedures were approved by our institutional animal research ethics committee with reference to the European Community guidelines for the use of experimental animals (GPH20120013).

### 2.5. Anti-Cancer Effect of the Extract on A549 Xenograft Mice and Analysis of Plasma Concentration of Paclitaxel

The in vivo anti-cancer effect of HDS-1 was determined using male BALB/c mice which were subcutaneously implanted with 1 × 10^7^ A549 cells which were trypsinized and resuspended in serum-free PBS. After the establishment of the model with the implanted tumor volume in BALB/c mice reaching 200–250 mm^3^, the BALB/c mice were divided randomly into 5 groups as follows (details see Table 1): vehicle, paclitaxel (PTX, *I.P.*), high-dosage HDS-1(600 mg/kg, I.G.), low-dosage HDS-1(200 mg/kg, I.G.), and PTX + WAB (I.G.). All xenograft mice received the dose solution (Table 1) in 5 different groups according to one of the following dosing schedules: (1) 50 mg/kg paclitaxel (*I.P.*) was given as a single injection every 2 days for the whole schedule, (2) vehicle, HDS-1 (both 600 and 200 mg/kg level) and PTX + WAB combination regimen daily for the first 8 days, (3) vehicle, HDS-1 (both 600 and 200 mg/kg level) and PTX + WAB combination regimen every 2 days from Day 9 to Day 34.

Tumor-bearing mice were weighed using an electronic scale and tumor sizes were surveyed with vernier calipers, and these procedures were conducted twice a week. Tumor weight was calculated from the tumor sizes according to the reported formula [30]: tumor volume (mm^3^) = (tumor length in mm) × (tumor width in mm)^2^/2. One and a half hours after the last administration on Day 34, all mice were sacrificed after blood was collected from the orbital sockets using fine capillaries. The anti-cancer activity of each agent group was determined by calculating the inhibition rate (IR = [1—(average tumor volume treated/average tumor volume control)] × 100%) and the survival ratio (number surviving mice/number of mice per group at the start of the study) for each group. The serum was separated and analyzed by method described previously [7].

### 2.6. Effect of Flavonoids and Lignoids on the Bidirectional Transport of Paclitaxel in Caco-2

Caco-2 cells were incubated in 37°C, 5% CO_2_ flanks for 2 weeks, and were further seeded onto 1.12 cm^2^ polycarbonate filter Transwells^TM^ at a density of 90,000 cells per well. The procedure was carried out with reference to a previous research [31] with little modifications.

All drug transport experiments were performed at 37°C and paclitaxel with or without flavonoids and lignoids was prepared using 4% BSA HBSS buffer. The apical (AP) media volume was 500 *μ*L and the basolateral (BL) volume was 1500 *μ*L. To determine the BL–AP transport, paclitaxel with flavonoids or lignoids was added to the BL compartment and its appearance in the AP compartment was monitored during the whole period. To determine absorptive transport (apical-to-basolateral; AP–BL), paclitaxel with flavonoids or lignoids was added to the AP compartment and its concentration in the BL compartment over time was tested. Verapamil serves as a positive control for this experiment and was added to both compartments prior to initiation. At 30, 60, 90, and 120 min, 100 *μ*L of media was removed from the receiver compartment followed by the addition of 100 *μ*L of preheated buffer as replenishment.

### 2.7. Effect of Flavonoids and Lignoids on Permeation of PTX through MDCK–MDR1 Cell Monolayers

To evaluate the inhibitory effect of flavonoids or lignoids on P-gp-induced reflux of PTX across the cultured cell monolayer, MDCK–MDR1cells were seeded on to Falcon 24-multiwell insert system at a density of 6 × 10^5^ cells/mL. The detailed method is applied as previous research [32] with some modification (such as time points of sampling including 0, 30, 60, 90, 120 min are selected, and C_0_ of PTX in the apical compartment is 1 *μ*g/mL).

All drug transport experiments were performed at 37°C and paclitaxel with or without flavonoids and lignoids was prepared using 4% BSA HBSS buffer. The apical (AP) media volume was 500 *μ*L and the basolateral (BL) volume was 1500 *μ*L. To determine the BL–AP transport, paclitaxel with flavonoids or lignoids was added to the BL compartment and its appearance in the AP compartment was monitored during the whole period.

The determination of paclitaxel in HBSS using UPLC-MS/MS as described previously [7].

### 2.8. Permeability Calculations and Statistical Analysis

The accumulated amount of paclitaxel appearing in the AP compartment (BL–AP) and the BL compartment (AP–BL) over time, dQ/dt, was used to calculate the apparent permeability (P_app_), and efflux ratio (ER, P_app_^BL–AP^/P_app_^AP–BL^) was determined according to previous research [31].

For ER and IR, statistical significance of the difference between the control and treatments (HDS-1,2,3) was evaluated using Student's *t*-test. Data are presented as Mean ± SD.

### 2.9. MTT Assay for Paclitaxel and HDS-1 or HDS-2 *in vitro*

Cell viability assay is chosen to determine the anti-cancer effect of the combination of paclitaxel with 2 HDS-1-derived extract fractions. 100 *μ*L A549 cells (2 × 10^5^ cells/mL) were seeded onto a 96-well plate. Following overnight incubation, the culture medium was aspirated, and the cells were incubated with paclitaxel or the combination of paclitaxel with various concentrations of HDS-2 or HDS-3 (the final concentrations were 100, 50, 25, 12.5, 6.25, 3.125, and 1.5625 *μ*g/mL) or treated with HDS-2 or HDS-3 in a complete culture medium for 24 h. The same volume of complete culture medium served as the negative control. Then, 20 *μ*l MTT solution (5 mg/mL) was added to each well, and the plates were incubated for 4 h. The medium was replaced by 150 *μ*l DMSO, which solubilized the MTT formazan salt. The absorbance of the solution was measured on a microplate reader (Vrioskan Flash, Thermo Scientific, USA) at 490 nm and the results were expressed as a percentage of the control cells.

### 2.10. Western Blotting

A549 cells are treated with paclitaxel with or without flavonoids or lignoids for 24 h, and then the cells are washed with cold PBS twice. Total cell extracts were lysed in lysis buffer (Ripa, Solarbio, China), and protein extract (60 *μ*g) was added into each well of 10% gel and separated through gel electrophoresis. Gels were soaked in transfer buffer (30 mM glycine, 16 mM Tris-HCl, and 20% methanol) and proteins in the gels were then blotted to polyvinylidene difluoride (PVDF) membranes. Nonfat dry milk in PBST (137 mM NaCl, 27 mM KCl, 100 mM Na2HPO4, 20 mM KH2PO4 0.05% tween 20, pH 7.4) was used for blocking nonspecific binding sites. The polyvinylidene difluoride membranes were then incubated with primary antibodies: anti-BCL-2 mAb (1 : 1000), anti-BAK mAB (1 : 2000), anti-MCL-1 pAb (1 : 3000), anti-pMCL-1 mAb (1 : 2000), anti-cyclinB1 mAb (1 : 1000), or anti-b-actin mAb (1 : 5000). Then, secondary antibodies (anti-mouse 1 : 5000, anti-rabbit–1:5000) were applied to the membranes. The ECL western blotting detection kit was used for detecting protein bands in the membranes. Protein bands were detected using the ECL Plus Kit (Huaan biotech, Hangzhou, China), imaged on Tanon 2000. Densitometry analysis was performed using ImageJ software to calculate the relative expression change after normalizing with *β*-actin.

### 2.11. Detection of Apoptosis by Flow Cytometry Analysis with Annexin V/PI Staining

A549 cells were incubated for 24 h, and then treated with paclitaxel with or without flavonoid or lignoids for another 24 h. Afterward, they were washed with PBS, counted, and adjusted to 1 × 10^6^ cells/ml. Apoptosis of A549 cells was determined by flow cytometry (FC500; Beckman Coulter, Brea, CA, USA) using the Annexin V-fluorescein isothiocyanate (FITC)/propidium iodide (PI) kit (MULTISCIENCES (LIANKE) BIOTECH, CO., LTD, Hangzhou, China). Staining was performed in accordance with the method described in the manufacturer's instructions. The rate of apoptosis was analyzed using MCF software (Beckman Coulter, Brea, CA, USA).

## 3. Results

### 3.1. Flavonoids and Lignoids Are Major Franctions of the Non-Taxane Component in HDS-1 Extract

**Figure 1:**
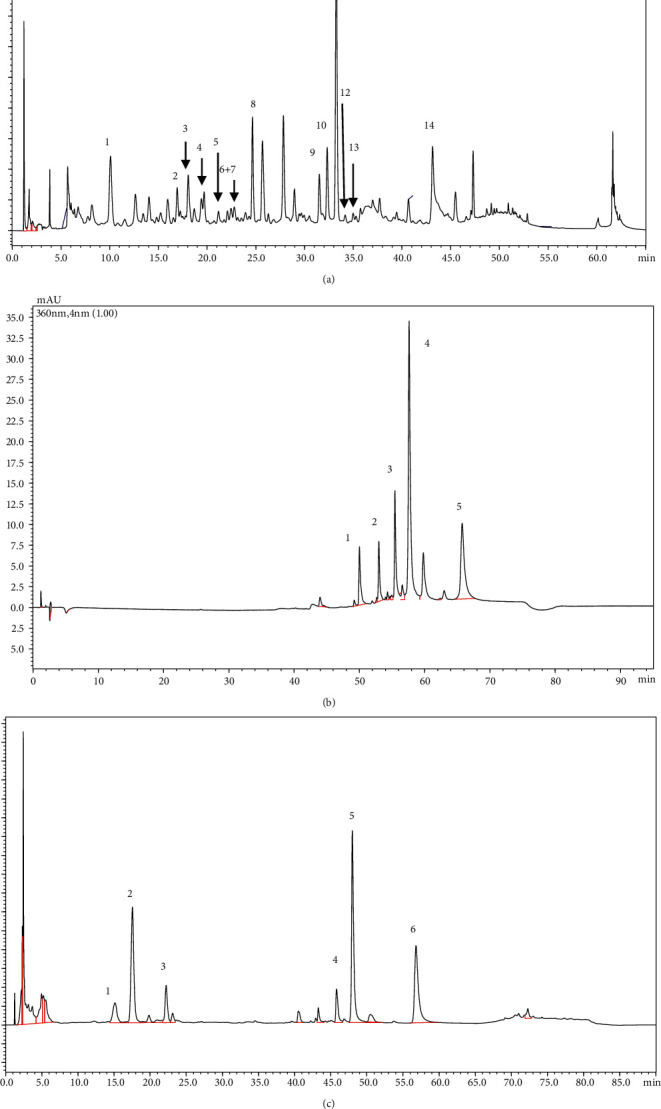
Chromatographic analysis of HDS-1, HDS-2, and HDS-3: (a) HPLC analysis of HDS-1 in 227 nm: 1.10-Deacetyl Baccatin III, 2. Baccatin III, 3. 7-Xylosyl-10-Deacetyl Cephalomannine, 4. 7-Xylosyl-10-Deacetyl Taxol, 5. Taxinine M, 6. 7-Xylosyl-10-Deacetyl Taxol C, 7.10-Deacetyl Taxol, 8.7-Xylosyl Taxol, 9.Cephalomannine, 10.7-Epi-10-Deacetyl Taxol, 11.Taxol, 12.Taxol C, 13.7-Epi Taxol, and 14.Taxacin; (b) HPLC analysis of HDS-2 in 360 nm: 1. amentoflavone, 2.bilobetin, 3. 7-O-methylamentoflavone, 4. ginkgetin, and 5. Sciadopitysin; (c) HPLC analysis of HDS-3 in 280 nm: 1. Secoisolariciresinol, 2.matairesinol, and 3. Brevitaxin.

In Figure 1, the HPLC-PDA profiles of HDS-1, HDS-2, and HDS-3 are demonstrated. 14 taxane, 5 flavonoids, and 3 lignoids are identified by comparing the retention time of standards and published data [33–37]. As shown in Table 2, the percentage of paclitaxel in HDS-1 is 2.50%, while paclitaxel (less than 0.002%) and other taxanes cannot be detected in either HDS-2 or HDS-3. The preclude of paclitaxel and taxane in HDS-2 and HDS-3 will facilitate further research.

In HDS-2, the content of total flavonoid and sciadopitysin is 31.46% and 29.81%, which is enriched from HDS-1 (9.59% and 7.67%), and matairesinol or lignoids cannot be detected (less than 0.01%). In HDS-3, the content of matairesinol (one of the lignoids) is 2.2% while 0.9% in HDS-1, and either sciadopitysin or flavonoids (less than 0.01%) cannot be detected. To sum up, refined flavonoids, which are partially reported previously [38], and lignoids are major components in HDS-2 and HDS-3, respectively, and they both exclude taxanes and componential overlap. Therefore, it made our further research possible to identify the responsible fraction for the absorptive improvement of paclitaxel.

### 3.2. Anti-Cancer Effect of the Extract on a549 Xenograft Mice and Analysis of Plasma Concentration of Paclitaxel

**Figure 2:**
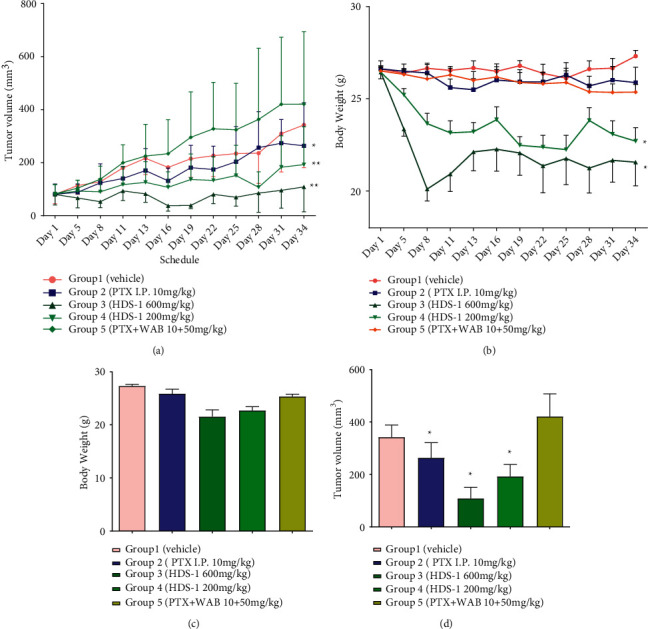
Inhibitory effect of HDS-1 on the growth of tumor in A549 xenograft-bearing mice. (a) Effect of HDS-1 on the growth of A549 tumor in A549-bearing nude mice. (b) Impact of the various HDS-1 components on the body weight of A549 -bearing mice through the 34-day treatment. (c) Impact of HDS-1 on the body weight of A549 xenograft-bearing mice on Day 34. (d) Effect of HDS-1 on the size of A549 tumor in nude mice on Day 34. *N* = 10 ^*∗*^*P* < 0.05,^*∗∗*^*P* < 0.01.

**Figure 3:**
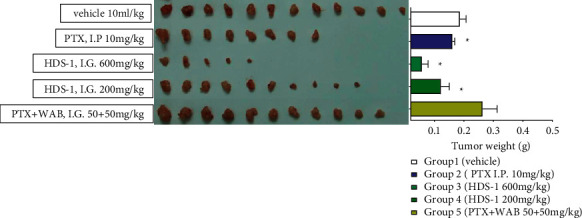
Inhibitory effect of HDS-1 on the weight of A549 tumor in A549 xenograft-bearing mice on Day 34 ^*∗*^*P* < 0.05.

**Figure 4:**
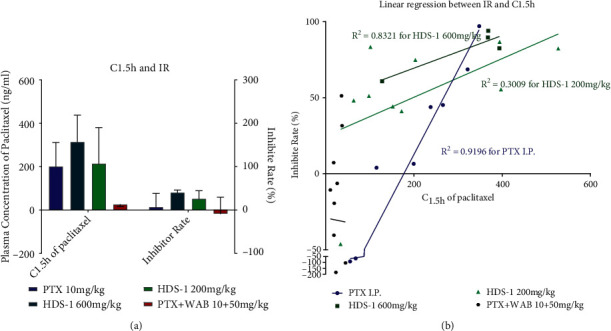
The inhibitory effect rate of HDS-1 on A549 tumor growth, plasma concentration of paclitaxel 1.5 h after administration in each group on Day 34 (a) and relationship of the inhibition rate and C_1.5h_ among oral route groups on Day 34 (b).

**Figure 5:**
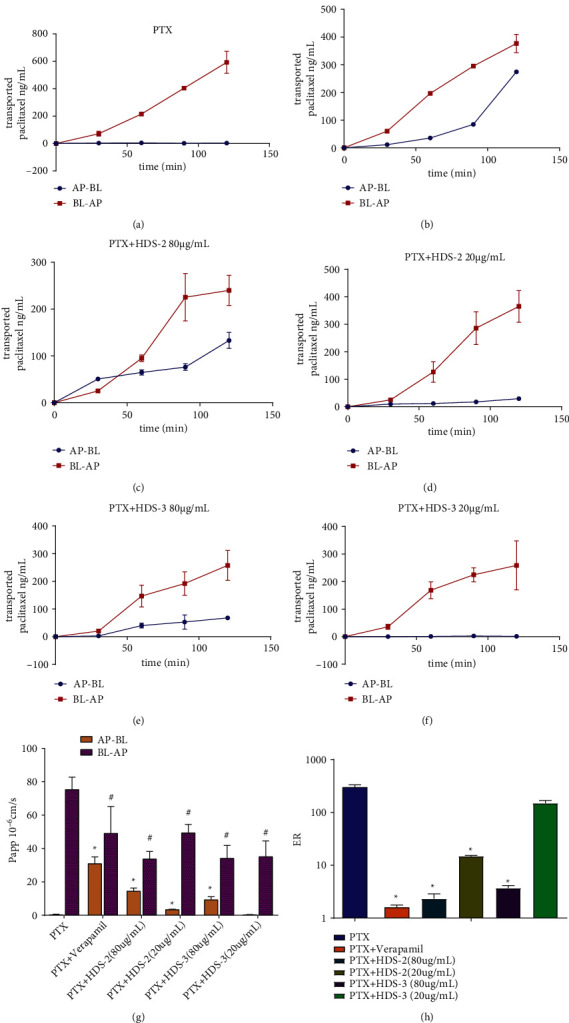
The effect of HDS-1-derived flavonoids (HDS-2) and lignoids (HDS-3) on the efflux of paclitaxel (P_app_) in the Caco-2 permeability experiment. ^*∗*^*P* < 0.05 versus P_app_ of AP–BL in PTX only. ^#^*P* < 0.05 versus P_app_ of BL–AP in PTX only. The effect of HDS-2 and HDS-3 on regulating the efflux ratio (ER) of paclitaxel in the Caco-2 bidirectional transport experiment.

The anti-tumor activities of HDS-1 (600 mg/kg, 200 mg/kg) and the combination of WAB (Wuweiz alcohol B, P-gp inhibitor [39] and paclitaxel, and paclitaxel (I.P.)) are summarized in Figures 2–5. Among the 3 oral route groups (I.G.), HDS-1 exerted potent anti-tumor activity in human xenograft models yielding an inhibition rate (IR) ranging from 60.85 to 93.99% (*P* < 0.01) at 600 mg/kg. At a dose of 200 mg/kg, HDS-1 still exhibited significant inhibition of tumor growth with the IR ranging from 41.16 to 86.68% (*P* < 0.01). In contrast, the combination (WAB + PTX) did not exhibit repression against the A549 growth, which is unexpected according to the previous study [39]; however, this result is reasonable since natural inhibitors may preferentially induce hapatic P-gp expression but not colonic P-gp in vivo [40, 41]. In an attempt to identify the correlation between efficacy and oral absorption of paclitaxel, the plasma levels of the 3 groups are analyzed using linear regression of individual C1.5 h and IR. As shown in Figure 4(a), the plasma concentration of paclitaxel 1.5 h after administration in HDS-1 (600 mg/kg, 200 mg/kg) and combination (WAB + PTX) is 314.6 ± 124.9 ng/mL, 214.3 ± 167.1 ng/mL, and 26.1 ± 11.1 ng/mL, respectively. In Figure 4(b), injection of PTX showed a greater relationship (*R*^2^ = 0.9196) between C_1.5h_ and inhibition ratio (IR), but R^2^s are 0.8321 and 0.3009 for 600 mg/kg and 200 mg/kg of HDS-1, respectively. Taken together, the anti-cancer activities of these 3 groups (oral route) are positively correlated with their C_1.5h_, and HDS-1 repressed the A549 tumor in a dose-dependent manner, but these results also indicate that the plasma concentration of paclitaxel is not a unique factor in affecting its efficacy in the HDS-1 treatment. For the I.P. group, paclitaxel (I.P.) exhibited mild activity against the growth of tumor in the first 15 days of the schedule, but it did not repress A549 tumor as before during the last 20 days (Figure 2). Consequently, paclitaxel (I.P.) exerted weak inhibitory activity against A549, which might be associated with multi-drug resistance (MDR) due to long-term exposure to paclitaxel [42]. In contrast, the xenograft A549 tumor was effectively inhibited by HDS-1 for the whole schedule, indicating components in HDS-1 are able to exert long-term activity (Figure 3). In terms of drug safety in this long-term experiment, low dosage of HDS-1 exhibited the advantage on the safety, which included fewer side effects and higher survival rates (Table 3). Additionally, A549 tumor-bearing mice remained in good condition with less toxic effects in oral administration through the whole schedule. The adverse effect of paclitaxel (I.P.) overweighed its efficacy in our research, which included hematochezia and seroperitoneum, which reflects accumulated serious organ damage in the paclitaxel (I.P.) group (Table 3). Our results indicate this extract can be applied as an effective and alternative regimen for treating cancer.

### 3.3. Flavonoids and Lignoids Increase the Uptake of Paclitaxel in the Caco-2 Permeability Model

**Figure 6:**
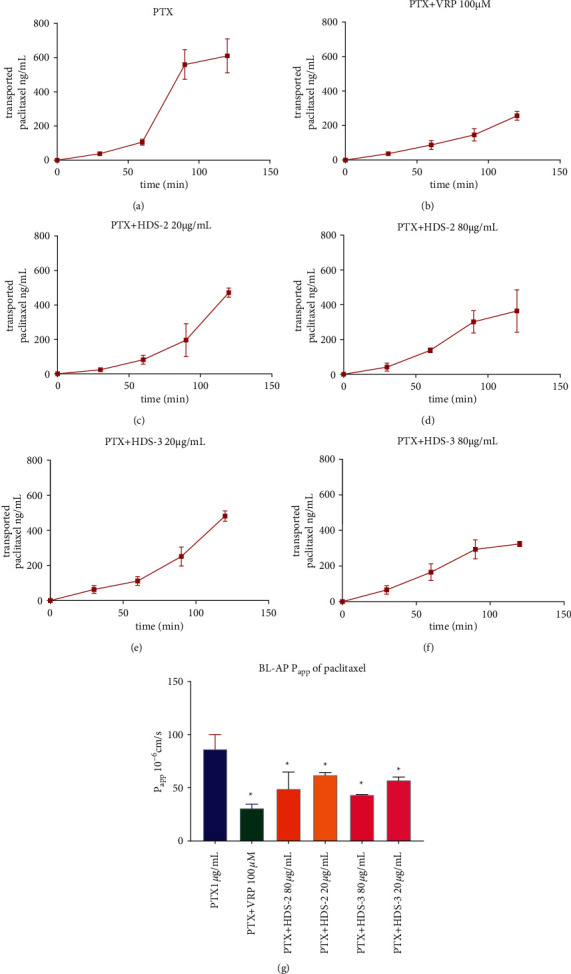
Inhibitory effect of HDS-2 and HDS-3 on P-gp-induced reflux of paclitaxel in the MDCK–MDR1 transport model.

Experiments were conducted using Caco-2 monolayers to compare the regulatory effect of HDS-2 and HDS-3, and verapamil on the absorptive transport and the efflux P_app_ of paclitaxel. The efflux ratio (ER) of paclitaxel was reduced to 1.3 in the presence of verapamil, while HDS-2 reduced the ER to about 2.4 and 14.9 in 80 and 20 *μ*g/mL, respectively. HDS-3 reduced the ER to less than 3.8 in 80 *μ*g/mL, but showed a mild effect in 20 *μ*g/mL (Figure 6). In terms of the apparent permeability coefficient (P_app_) of paclitaxel (Table 4), HDS-2 declined the efflux of paclitaxel from basolateral (BL) to apical (AP) and also enhanced its permeate from AP to BL, and thus weakened the ER of paclitaxel by more than 20-folds when compared to the control group. HDS-3 decreased the P_app_ (BL-AP) of paclitaxel from 75.26 ± 7.43 × 10^−6 ^cm/s to 34.06 ± 7.76 × 10^−6 ^cm/s in 80 *μ*g/mL, which was equal to HDS-2 (80 *μ*g/mL). However, HDS-2 exerted more potent effect than HDS-3 on promoting the absorption of paclitaxel in the AP–BL direction (Figure 5 and Table 4), which proves that HDS-2 is more effective in inhibiting P-gp-induced efflux of paclitaxel. Interestingly, in our previous study, HDS-1, containing HDS-2 and HDS-3, reduced the ER to 1.1 [7], which means the efflux of P-gp is completely prohibited. Taken together, HDS-2 might synergize more effectively with HDS-3 on inhibiting P-gp function than they did alone.

### 3.4. Flavonoids and Lignoids Reduce the Efflux of Paclitaxel in the MDCK–MDR1 Model

To further validate the permeability-enhancing effect of flavonoids or lignoids, the MDCK–MDR1 cell line is applied to evaluate their effect on P-gp-induced reflux of paclitaxel. As Figure 6(a)–6(f)) shows, compared with paclitaxel donated in the basal compatment, transported paclitaxel from BL–AP is significantly reduced by combining verapamil (100 *μ*M), HDS-2 (80 and 20 *μ*g/mL), and HDS-3 (80 and 20 *μ*g/mL) in the time course of 0–120 minutes. In terms of apparent permeability (P_app_) in Figure 6(g), P_app_ of paclitaxel is reduced from 86.43 ± 14.09 × 10^−6^ cm/s (PTX alone) to 30.85 ± 4.08 × 10^−6^ cm/s (PTX + VRP 100 *μ*M), 62.04 ± 2.72 × 10^−6^ cm/s (HDS-2 20 *μ*g/mL), 49.02 ± 16.14 × 10^−6 ^cm/s (HDS-2 80 *μ*g/mL), 57.14 ± 3.38 × 10^−6^ cm/s (HDS-3 20 *μ*g/mL), and 43.46 ± 0.55 × 10^−6^ cm/s (HDS-2 80 *μ*g/mL). This finding implies that the bio-enhancing effect of flavonoids (HDS-2) and lignoids (HDS-3) is associated with a reduction in the P-gp function in enterocytes.

### 3.5. Flavonoids and Lignoids Enhance Paclitaxel-Induced Cytotoxic in vitro

**Figure 7:**
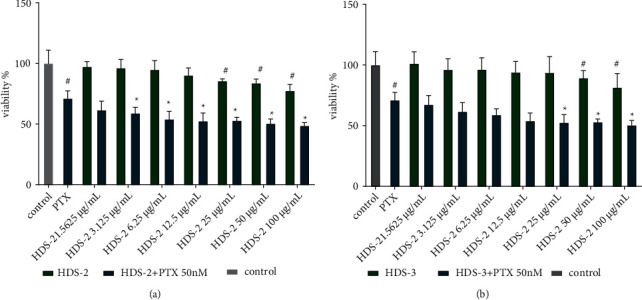
The synergistic cytotoxicity of paclitaxel and HDS-2, HDS-3 on A549 in vitro. (a) HDS-2 alone and combination of HDS-2 with paclitaxel; (b) HDS-3 alone and combination of HDS-3 with paclitaxel ^*∗*^*P* < 0.05: versus paclitaxel (50 nM) alone; ^#^*P* < 0.05: versus control.

Paclitaxel inhibits the proliferation of A549 cells while HDS-2 or HDS-3 exerts mild inhibitory effect on the growth of A549 (Figure 7). The combination of paclitaxel with various doses extracts (100, 50, 25, 12.5, 6.25, 3.125, and 1.5625 *μ*g/mL) was used to treat A549 cells for 24 h. The anti-proliferative effect of paclitaxel or the combination of paclitaxel with HDS-2 or HDS-3 on A549 was assessed by the MTT assay, and the results showed that paclitaxel inhibited the proliferation of A549 cells efficiently by approximately 28% at a concentration of 50 nM for 24 h. The inhibitory effect of paclitaxel is enhanced by co-incubating HDS-2 of 3.125–100 *μ*g/mL, which yields the viability of A549 ranging from 58.92% to 48.69% (*P* < 0.05). However, HDS-3 exerts a synergistic effect with paclitaxel against A549 growth in high concentration. Taken together, HDS-2 is more efficient than HDS-3 in terms of potentiating paclitaxel-induced cytotoxic effect in vitro, while HDS-2 and HDS-3 exhibit mild inhibitory effect toward A549 growth.

### 3.6. Flavonoids and Lignoids Synergize with Paclitaxel on Regulating BCL-2 Family Proteins' Expressions

**Figure 8:**
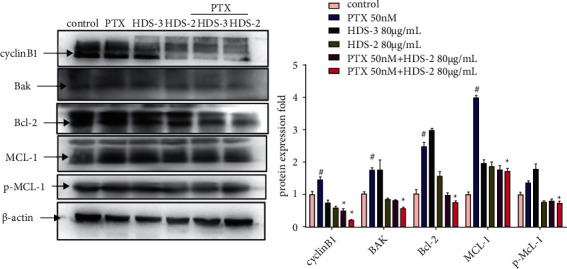
Regulative effect of paclitaxel combined with HDS-2 or HDS-3 on the expressions of BCL-2 family proteins in A549.^#^*P* < 0.05 versus control, and ^*∗*^*P* < 0.05 versus PTX group.

To further investigate the synergistic effect between paclitaxel and flavonoids or lignoids, the expression of apoptosis-related BCL-2 family proteins is evaluated by western blot. After 24 h treatment of PTX 50 nM, the expressions of cyclinB1, BAK, BCL-2, MCL-1, and pMCL-1 are significantly upregulated (Figure 8), indicating the A549 cell is arrested by G2/M due to the abnormal expression of cyblinB1 and A549 cells are possibly adapted to PTX challenge in 24 h exposure. In contrast, the expressions of cyclinB1, BAK, BCL-2, and pMCL-1 are remarkably inhibited by the combination of PTX 50 nM and HDS-2 (80 *μ*g/mL) or HDS-3 (80 *μ*g/mL) when compared with the control group. The expression of MCL-1 is upregulated by PTX but it is slightly reversed to 1.5-fold in the combinative treatment of PTX and HDS-2. Interestingly, mono treatment of HDS-2 or HDS-3 cannot inhibit all BCL-2 proteins except cyclinB1, and this finding suggests that the synergetic effect of PTX and flavonoids or lignoids may be associated with the downregulation of BCL-2 family proteins.

### 3.7. Flavonoids Synergize with Paclitaxel on Causing Different Cell Death

**Figure 9:**
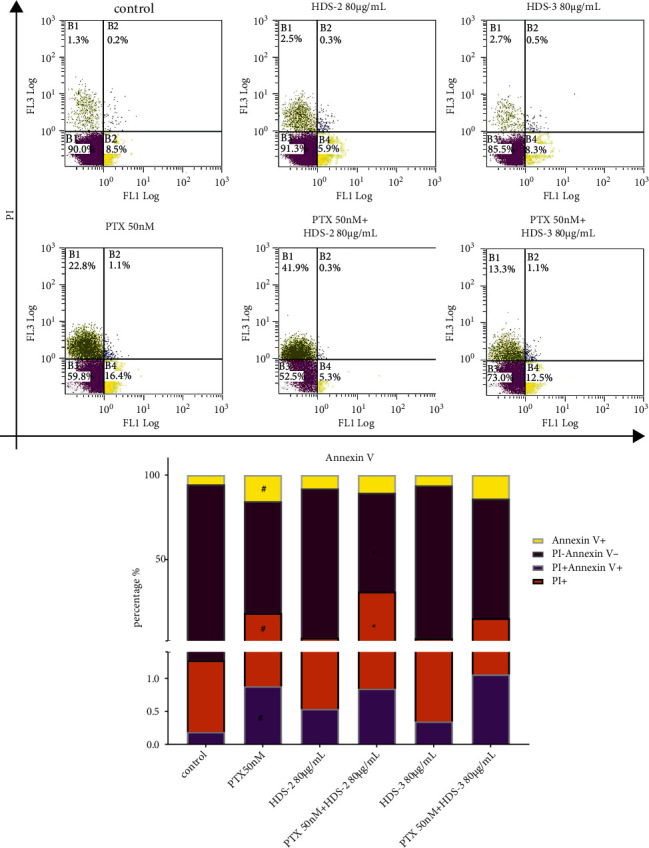
Regulative effect of HDS-2 and HDS-3 on PTX-induced A549 cell apoptosis. n = 3, ^#^*P* < 0.05 versus control, and ^*∗*^*P* < 0.05 versus PTX group.

To further investigate the cytotoxic effect of PTX-flavonoids or PTX-lignoids combination, PI and Annexin V staining experiments are conducted using flow cytometry. As Figure 9 shows, the PTX treatment can induce a percentage of cells that are PI positive (approx. 22.8%) and Annexin V positive (approx. 16.3%), indicating A549 cells have morphological changes in the cell membrane integrity and structures after PTX (50 nM) treatment. Interestingly, the percentage of cells that are PI positive is enhanced by combining HDS-2 80 *μ*g/mL, even though they do not demonstrate the potential effect of changing A545 cell membrane morphology (mild PI and Annexin V staining). Specially, the combination of HDS-2 and PTX increased PTX-induced necrosis (PI positive) from 22.8% to 41.9% and reduced PTX-induced apoptosis from 17.5% to 5.63%. This finding suggests that the PTX-induced damage of A549 cell integrity is strengthened by the coexisting component in HDS-2, and the synergistic effect of flavonoids or lignoids will trigger different cell death of A549 under paclitaxel exposure.

## 4. Discussion

**Figure 10:**
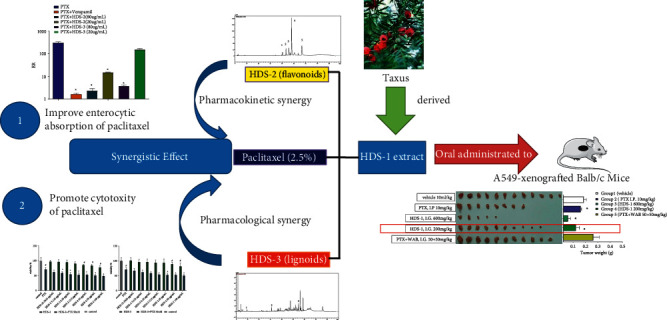
Graphic abstract of oral-route anti-cancer effect of HDS-1 and the synergistic effect between flavonoids or lignoids and paclitaxel.

It is a common strategy to improve paclitaxel's oral absorption through combining P-gp inhibitors; however, a suitable P-gp inhibitor remains unavailable though great effort has been devoted [43] in preclinic and clinic research. Clinical trial of these inhibitors is partially terminated for their serious toxicity [44]. As an alternative to modern medicine, herbal medicine and traditional Chinese medicine (TCM) are promising to provide natural inhibitors against P-gp-induced efflux [45]. In the case of our previous research, oral bioavailability of paclitaxel was elevated by 9.7-fold in paclitaxel-containing HDS-1 extract [7], implying that some components may serve inherent bioenhancers in HDS-1 and may enhance the anti-cancer activity of PTX through oral administration. Therefore, the aims of this study were to evaluate the anti-cancer effect of the Taxus extract (HDS-1) as well as to explore the contributive effect of relevant components. Flavonoids and lignoids are isolated from the crude extract HDS-1 according to previous methods [46, 47], and their effects on oral absorption and cytotoxicity of paclitaxel were explored. The most significant finding of our research is that oral administration of paclitaxel-containing extract exerts anti-cancer effect in A549-bearing nude mice, and HDS-1-derived flavonoids and lignoids contribute to their anti-cancer effect through enhancing enterocyte absorption of paclitaxel and augmenting cytotoxicity and BCL-2-related cell death of paclitaxel (Figure 10). The HDS-1 extract serves as a spontaneous oral-route anti-cancer complex, which is comprised of paclitaxel, bioenhancers, and cytotoxic enhancers. The finding highlights that it is prospective strategy to conquer the barrier for oral-route paclitaxel through inherent drug–drug interaction in the herbal extract.

Both flavonoids and lignoids contribute to the anti-cancer activity of HDS as bioenhancers of paclitaxel in enterocytes of oral route.

*C*_max_ is a pharmacokinetic parameter which is indicative of the oral absorption of paclitaxel (PTX); it is at 1.5 h that the plasma concentration of PTX reached the peak in the oral absorption of HDS-1 according to previous research [7]. Therefore, *C*_max_ is indexed as a reference to evaluate the exposure of HDS-1 in A549-bearing nude mice. Furthermore, the relationship between *C*_max_ and the inhibition rate is analyzed by linear regression, and it is validated that the plasma concentration of PTX is still highly associated with the anti-cancer effect of HDS-1 in oral administration groups. These preliminary outcomes indicate that the components of HDS-1 may serve as inherent bioenhancers for PTX in oral absorption and PTX is an active component contributing to HDS-1's anti-cancer activity. Since PTX is hard to penetrate into the colon epithelial, it is assumed that some components may enhance its permeation in the colon. This assumption is further validated by our *in vitro* experiment, and the action mechanism of this enhancement is related to the inhibition of specific agent transporters.

A variety of agent metabolism enzymes and transporters are expressed in the colon epithelial, and they block the penetration of paclitaxel (PTX) into the enterocytes. P-glycopretein (P-gp) is one canonical transporter that mediates the oral absorption of PTX. Specifically, PTX is pumped out from the enterocytes through P-gp; in other words, P-gp blocks the permeation of PTX through the epithelia. To date, P-gp inhibitors attract numerous investigators' attention and devotion, but they are not available in clinic trials. Our previous finding is indicative of the inherent P-gp inhibitors in the HDS-1 extract. In an attempt to identify the bioenhancers in HDS-1 and their mediation on potential target P-gp, two models of drug transportation are employed. The Caco-2 bidirectional permeability assay is a well-developed monolayer model for evaluating the permeation of the agent; it is extensively used due to its enterocyte-like phenotype. The differentiated Caco-2 is capable of mimicking the physical and metabolic barrier of the intestinal epithelium, and it expresses several drug transporters including P-gp [48]. In addition, the MDCR–MDR1 model is a monolayer model with overexpressing human P-gp (encoded by MDR1), and it is applied as a validation method for HDS-1's mediation mechanism regarding specific drug transporters. In our research, it is confirmed that paclitaxel is hardly absorbed in the oral route due to carrier-mediated efflux in the Caco-2 bidirectional permeability assay [49]. Our research also finds that both HDS-2 (flavonoid) and HDS-3 (lignoids) significantly inhibit the efflux of paclitaxel in the enterocytes in the two models. Specifically, inhibition of paclitaxel efflux from the basolateral-to-apical (BL-AP) direction contributes to absorption improvement, indicating oral improvement is mainly associated with potent reduction in P-gp-induced paclitaxel efflux. Therefore, flavonoids and lignoids may serve as natural P-gp inhibitors for paclitaxel. Moreover, P-gp is further confirmed to be interrupted by HDS-2 or HDS-3 in the MDCK–MDR1 monolayer model. Human P-gp is highly expressed in MDCK-MDR1 and it is abundant than other drug transporters; P-gp-mediated efflux is mainly responsible for the reflux of PTX. Therefore, P-gp activity is assumed to be restricted by flavonoids or lignoid of HDS-1 in the MDCK–MDR1 model. Accumulating evidence supports that herbal extracts serves as a complexity which may integrate various effects of different components to achieve greater inhibition of P-gp [50]. More specifically, since P-gp is one of ATP-binding cassette transporters, whose function critically depends on ATP hydrolysis [51] and cytomembrane expression, P-gp-inhibiting effects of flavonoids and lignoids may stem from various effects: deleting ATP [52], destabilizing P-gp through phosphorylation [53], and downregulating the protein expression of the cytomembrane [54]. Therefore, the inhibitory mechanism of P-gp function is worth an in-depth research for refining specific enterocytic P-gp inhibitors from HDS-derived flavonoids. Moreover, nuclear receptors such as NF*κ*B and PXR, which are candidate targets of flavonoids and lignoids, are likely to involve in mediating the P-gp expression in the upstream pathway [55]. Therefore, compared with synthesized P-gp inhibitors, plant-derived inhibitors may inhibit enterocytic P-gp through different targets, which may render options for P-gp inhibitors specific for enterocytes and avoid triggering side effects of P-gp inhibitors for other tissues [56]. Additionally, tissue-specific P-gp inhibitors may explain the unfavored result in the combination treatment of PTX with P-gp inhibitor schisandrol B, which is well-established herbal component for improving absorption in liver. It seems that schisandrol B is a liver-specific P-gp inhibitor but may not function well in enterocytes. Therefore, it is confirmed that the anti-cancer effect of HDS-1 partially is associated with elevated paclitaxel exposure which is raised by HDS-1-derived bioenhancers or enterocyte-specific P-gp inhibitors in the HDS-1 extract.

Both flavonoids and lignoids contribute to the anti-cancer effect of HDS as cytotoxic enhancers for paclitaxel.

Cytotoxic effect is still the main activity for the anti-cancer effect of PTX, and its efficacy is associated with the systemic exposure of PTX. However, even though the systemic exposure of HDS-1 (200 mg/kg) is much lower than that of PTX injection, HDS-1 exhibits more potent efficacy than the PTX injection. It is possible that other components of the HDS-1 extract enhance the cytotoxic effect of PTX. Synergistic effect is found between flavonoid or lignoids and PTX, and a combination of harmless flavonoids or lignoids with PTX results in cytotoxicity enhancement. Therefore, this finding indicates that other action mechanisms may contribute to the efficacy of the HDS-1 extract beyond bioenhancers for paclitaxel. According to previous findings, two possibilities are posed as follow: Taxus-derived taxane compounds may contribute to the *in vivo* anti-cancer effect due to the cytotoxicity property [57], or flavonoids or lignoids may exert a synergistic effect to enhance PTX's efficacy [58, 59]]. The latter hypothesis is confirmed by the MTT assay in our investigation, and we find that HDS-2 (flavonoids) and HDS-3 (lignoids) exert a synergistic effect with paclitaxel toward A549 growth *in vitro*. The action mechanism of this synergism is assumed to be associated with augmenting the vulnerability of cancer cells to PTX-induced programmed cell death (PCD).

PCD is conventionally recognized to be the action mechanism of PTX's cytotoxicity, and it includes apoptosis, necrosis, necroptosis, autophagic death, and ferroptosis. BCL-2 family members, which mediate the faith of cancer cells, are closely associated with the PCD regulation [60–62] and switch of various PCD [63]. BCL-2 family proteins are divided into 2 kinds as regard to their biofunction: pro-apoptosis (such as BAX) and anti-apoptosis (such as BCL-2 and MCL-1). It is reported that BCL-2 proteins' expression is not sensitive to paclitaxel due to tumor genetic or epigenetic regulation, which inhibits PCD and renders the cancer cell to survive in response to PTX. In our case, the expression of BCL-2 is prone to be upregulated after 24 h incubation with low levels of PTX in the A549 cell line. BCL-2 regulation shows inconsistency with previous research [64] due to the adaptive regulation (abnormal BCL-2 expression) of carcinoma cells in low levels of PTX. Moreover, the combination of PTX and flavonoid or lignoid attenuates the adaption through inhibiting BCL-2, MCL-1, and p-MCL-1, which maintain cell survival as anti-apoptosis factors in A549 [65]. This outcome indicates that BCL-2-related mitochondria function is interrupted by flavonoids or lignoids and consequently lead to PCD. Moreover, in the cytometry experiment, this combination increases the percentage of cells undergoing PCD, and especially switch from apoptosis or normal cells to necrosis cells. Taken together, BCL-2-inhibiting effect of flavonoid and lignoid may contribute to the apoptosis–necrosis transition in the context of PTX according to previous research [66].

Additionally, there is limitation in our research; it cannot be exclusive that taxane components of the HDS-1 extract may contribute to the anti-cancer effect of HDS, but their toxicity remains a major concern for development as adjuvant treatment. Therefore, validation of the taxane's contribution to the anti-cancer property of HDS-1 as well as its safety is still an ongoing research in our lab.

## 5. Conclusions

Conclusively, the present study provides corroborative evidence for the first time that HDS-1 possesses anti-cancer activity through oral route, and HDS-2 (flavonoids) and HDS-3 (lignoids), derived from Taxus *yunnanensis* Cheng et L.K. Fu, can serve as inherent bioenhancers and cytotoxicity-enhancers for improving the oral bioavailability and anti-cancer activity of paclitaxel. Moreover, HDS-1 exhibited more sustainable efficacy with less side effects in low dosage, and the dosage of HDS-1 should be refined to achieve better outcomes. Therefore, HDS-1 has the potential for developing a long-term regimen for treating cancer under suitable dosage adjustments. To extrapolate the core of Chinese medicine in this research, it highlights the synergy of therapeutic agents and auxiliary components in cancer remedy.

## Figures and Tables

**Table 1:** Preparation of the dosing solution for the *in vivo* experiment.

Group number	Group name	Solution and dosage	Administration route
1	Vehicle	Vehicle (10 mL/kg)	Intragastric (*I.G.*)
2	PTX (*I.P.*)	Paclitaxel (10 mg/kg)	Intraperitoneal injections (*I.P.*)
3	HDS-1 (600 mg/kg)	HDS-1 extract (600 mg/kg)	*I.G.*
4	HDS-1 (200 mg/kg)	HDS-1 extract (200 mg/kg)	*I.G.*
5	PTX + WAB	Paclitaxel and WAB (10 + 50 mg/kg)	*I.G.*

**Table 2:** The content of paclitaxel, sciadopitysin, matairesinol, and total flavonoids in HDS-1,2,3.

	Major component	Dectected UV wavelength (nm)	Content of relative components			
			Paclitaxel	Sciadopitysin	Matairesinol	Total flavonoids
HDS-1	Taxane, flavonoids, and lignoids	227 for taxane	2.50%	7.67%	0.9%	9.59%
HDS-2	Flavonoids	360	<0.002%	29.81%	<0.01%	31.46%
HDS-3	Lignoids	280	<0.002%	<0.01%	2.2%	<0.01%

**Table 3:** The anti-cancer effect and adverse effect of HDS-1 on A549 xenograft-bearing mice.

Group	Administration dosage mg/kg	Mortality	Inhibition rate %	Survival rate %	Side effect
Vehicle	—	0	—	100	—
PTX I.P.	10	4	38.60 ± 64.87	66.7	Seroperitoneum and hematochezia
HDS-1 (H)	600	6	86.1 ± 12.94	54.55	—
HDS-1 (L)	200	1	65.7 ± 38.71	91.1	—
PTX + WAB	10 + 50	0	−17.6 ± 77.88	100	—

**Table 4:** The effect of HDS-2 and HDS-3 on enterocytic permeability (P_app_ and ER) of paclitaxel in the Caco-2 permeability experiment. ^*∗*^*P* < 0.05 versus PTX group.

Group	Agents		P_app_ (×10^−6 ^cm/s)		Efflux ratio ER
	Targeted agent	Combinative agents	AP–BL	BL–AP	
PTX	Paclitaxel 1 *μ*g/mL	—	0.25 ± 0.43	75.26 ± 7.43	299.26 ± 34.25
PTX + VRP	Paclitaxel 1 *μ*g/mL	Verapamil 100 *μ*M	30.85 ± 4.08^*∗*^	49.01 ± 16.15^*∗*^	1.59 ± 0.18^*∗*^
PTX + HDS-2	Paclitaxel 1 *μ*g/mL	HDS-2 80 *μ*g/mL	14.45 ± 1.83^*∗*^	33.72 ± 4.52^*∗*^	2.33 ± 0.55^*∗*^
PTX + HDS-2	Paclitaxel 1 *μ*g/mL	HDS-2 20 *μ*g/mL	3.31 ± 0.30^*∗*^	49.24 ± 5.12^*∗*^	14.89 ± 0.53^*∗*^
PTX + HDS-3	Paclitaxel 1 *μ*g/mL	HDS-3 80 *μ*g/mL	9.21 ± 1.88^*∗*^	34.06 ± 7.76^*∗*^	3.70 ± 0.43^*∗*^
PTX + HDS-3	Paclitaxel 1 *μ*g/mL	HDS-3 20 *μ*g/mL	0.23 ± 0.20	35.02 ± 9.53^*∗*^	150.62 ± 17.98

## Data Availability

Data are included in the supplement file.

## Supplementary Materials

Supplementary Materials Supplementary Materials. Data are available in the supplement file.
