# Supplementary material for: Paclitaxel-Containing Extract Exerts Anti-Cancer Activity through Oral Administration in A549-Xenografted BALB/C Nude Mice: Synergistic Effect between Paclitaxel and Flavonoids or Lignoids
Source: Evid Based Complement Alternat Med. 2022 Apr 25;2022:3648175. doi: 10.1155/2022/3648175 (PMC9060980; doi:10.1155/2022/3648175)
Supplement: Supplementary Materials — Data are available in the supplement file. [file 3648175.f1.zip › 3648175.f1/Figure 1 HDS-1 (1).docx]

HDS-1 TIC图谱

HDS-1 PDA图谱

HDS-1 227nm

280nm

**360nm**
