# Supplementary material for: Paclitaxel-Containing Extract Exerts Anti-Cancer Activity through Oral Administration in A549-Xenografted BALB/C Nude Mice: Synergistic Effect between Paclitaxel and Flavonoids or Lignoids
Source: Evid Based Complement Alternat Med. 2022 Apr 25;2022:3648175. doi: 10.1155/2022/3648175 (PMC9060980; doi:10.1155/2022/3648175)
Supplement: Supplementary Materials — Data are available in the supplement file. [file 3648175.f1.zip › 3648175.f1/Figure 1 HDS-2 (1).pdf]

==== Shimadzu LCsolution 分析报告 ====

采集人	: Admin
样品名称	: 黄酮（GQ）
样品 ID	: 黄酮（GQ）
样品架	: 1
样品瓶#	: 69
进样体积	: 20 uL
数据文件名	: 黄酮（GQ）.lcd
方法文件名	: Curosil PFP柱测定紫杉烷类-20120425-3楼.lcm
批处理文件名	: 20120620-3.lcb
报告文件名	: Default.lcr
数据采集	: 2012-6-20 23:22:07
数据处理	: 2012-6-21 8:44:33

<色谱图>

D:\LabSolutions\Data\红豆杉\20120620\黄酮（GQ）.lcd

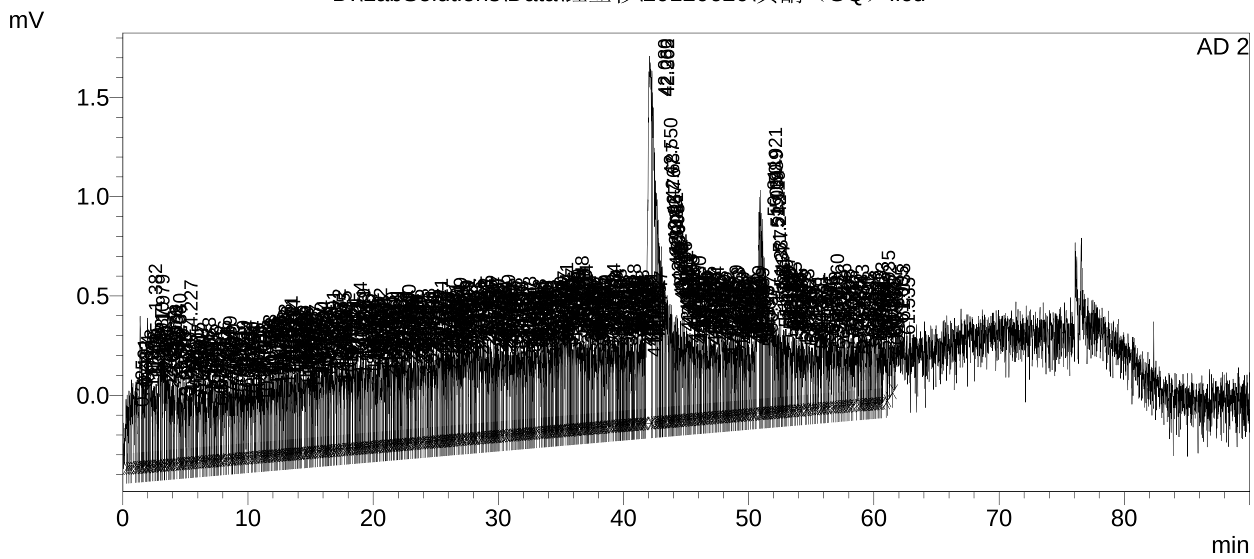

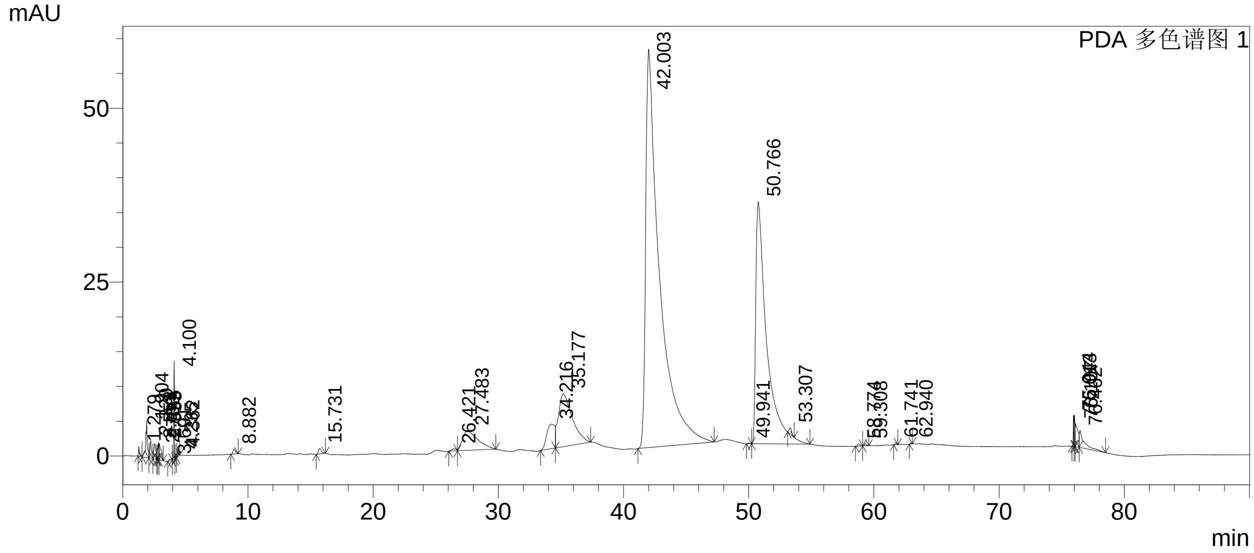

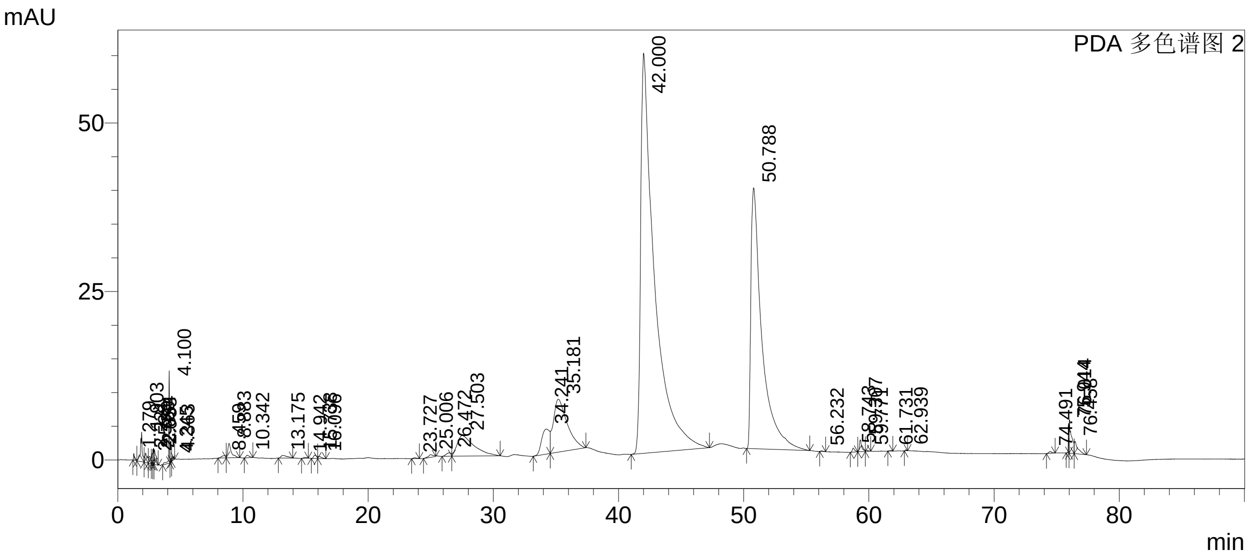

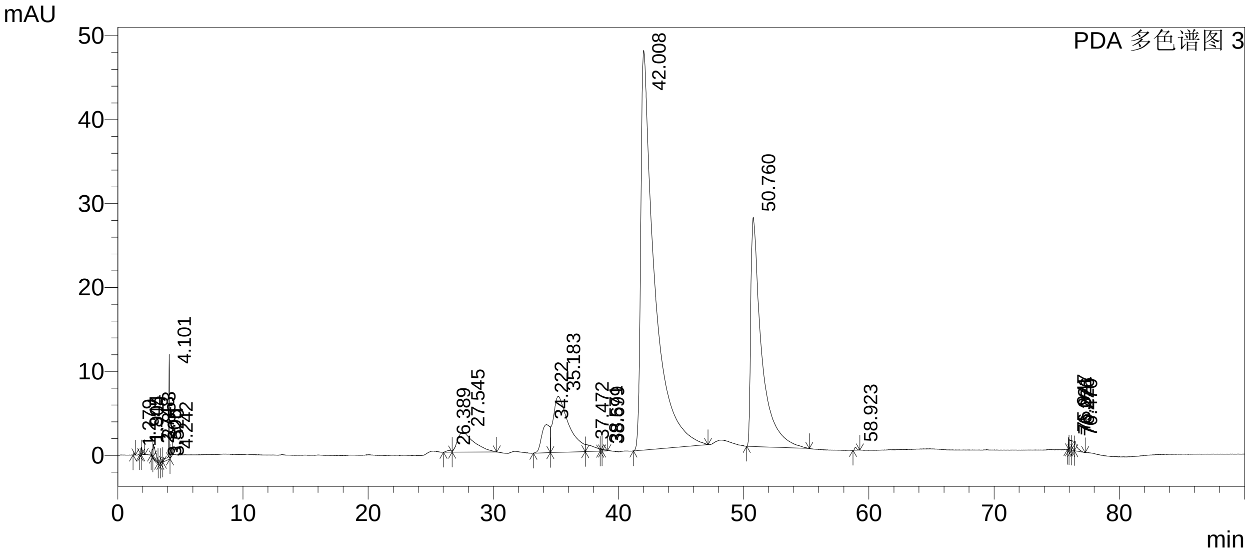

1 AD 2/
2 PDA 多色谱图 1/254nm 4nm
3 PDA 多色谱图 2/280nm 4nm
4 PDA 多色谱图 3/360nm 4nm

峰表

PDA Ch3 360nm 4nm

峰#	保留时间	面积	高度	面积 %	高度 %
1	1.279	1365	389	0.021	0.348
2	1.845	2766	764	0.043	0.682
3	1.904	3345	877	0.052	0.784
4	2.748	4648	869	0.073	0.776
5	2.853	21326	1632	0.333	1.458
6	3.305	1043	187	0.016	0.167
7	3.520	1150	166	0.018	0.148
8	4.101	46267	12483	0.723	11.149
9	4.242	2778	374	0.043	0.334
10	26.389	5314	187	0.083	0.167
11	27.545	216319	2358	3.380	2.106
12	34.222	150804	3352	2.356	2.994
13	35.183	577101	6692	9.016	5.977
14	37.472	40658	819	0.635	0.731
15	38.571	2275	262	0.036	0.234
16	38.699	3452	218	0.054	0.195
17	42.008	3620240	47637	56.558	42.545

峰#	保留时间	面积	高度	面积 %	高度 %
18	50.760	1645345	27320	25.705	24.400
19	58.923	4486	382	0.070	0.341
20	75.947	5426	1477	0.085	1.319
21	76.021	12371	1231	0.193	1.099
22	76.226	15066	1169	0.235	1.044
23	76.470	17333	1124	0.271	1.004
总计		6400877	111969	100.000	100.000
