# Supplementary material for: Paclitaxel-Containing Extract Exerts Anti-Cancer Activity through Oral Administration in A549-Xenografted BALB/C Nude Mice: Synergistic Effect between Paclitaxel and Flavonoids or Lignoids
Source: Evid Based Complement Alternat Med. 2022 Apr 25;2022:3648175. doi: 10.1155/2022/3648175 (PMC9060980; doi:10.1155/2022/3648175)
Supplement: Supplementary Materials — Data are available in the supplement file. [file 3648175.f1.zip › 3648175.f1/Figure 1 HDS-3 (1).pdf]

==== Shimadzu LCsolution 分析报告 ====

采集人	: Admin
样品名称	: 木脂素（GQ）
样品 ID	: 木脂素（GQ）
样品架	: 1
样品瓶#	: 64
进样体积	: 20 uL
数据文件名	: 木脂素（GQ）.lcd
方法文件名	: Curosil PFP柱测定紫杉烷类-20120425-3楼.lcm
批处理文件名	: 20120620-2.lcb
报告文件名	: Default.lcr
数据采集	: 2012-6-20 15:49:29
数据处理	: 2012-6-21 8:46:29

<色谱图>

D:\LabSolutions\Data\红豆杉\20120620\木脂素（GQ）.lcd

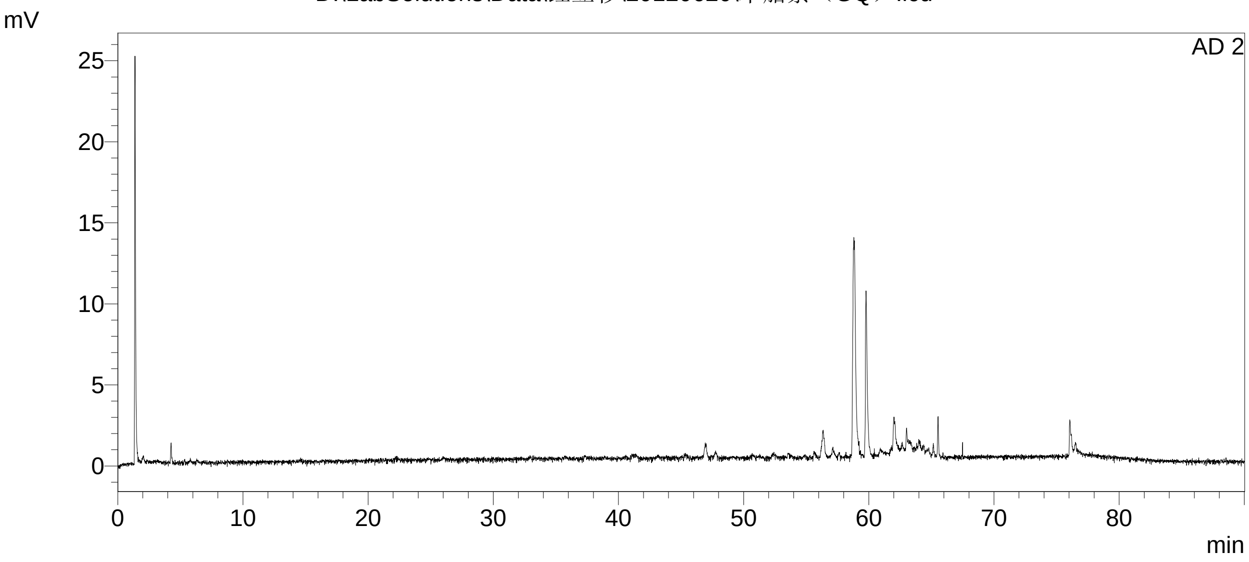

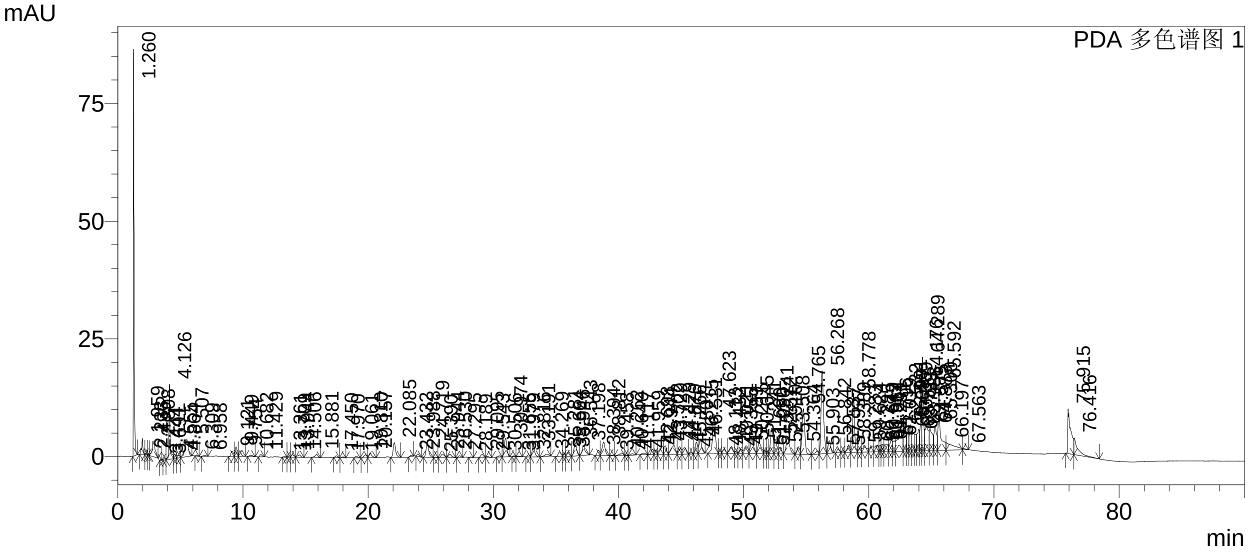

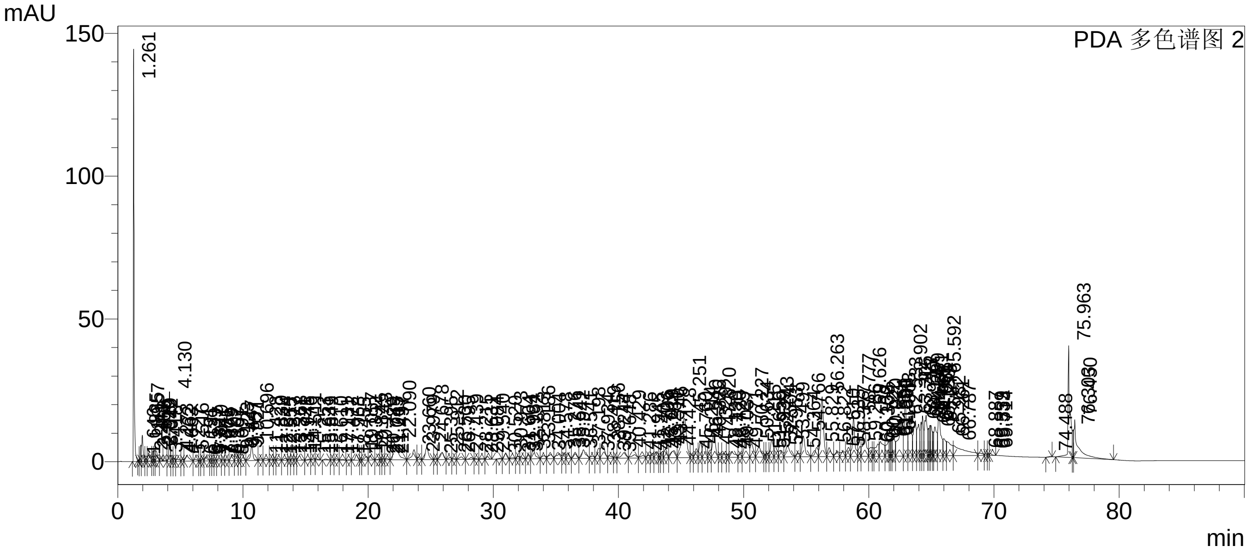

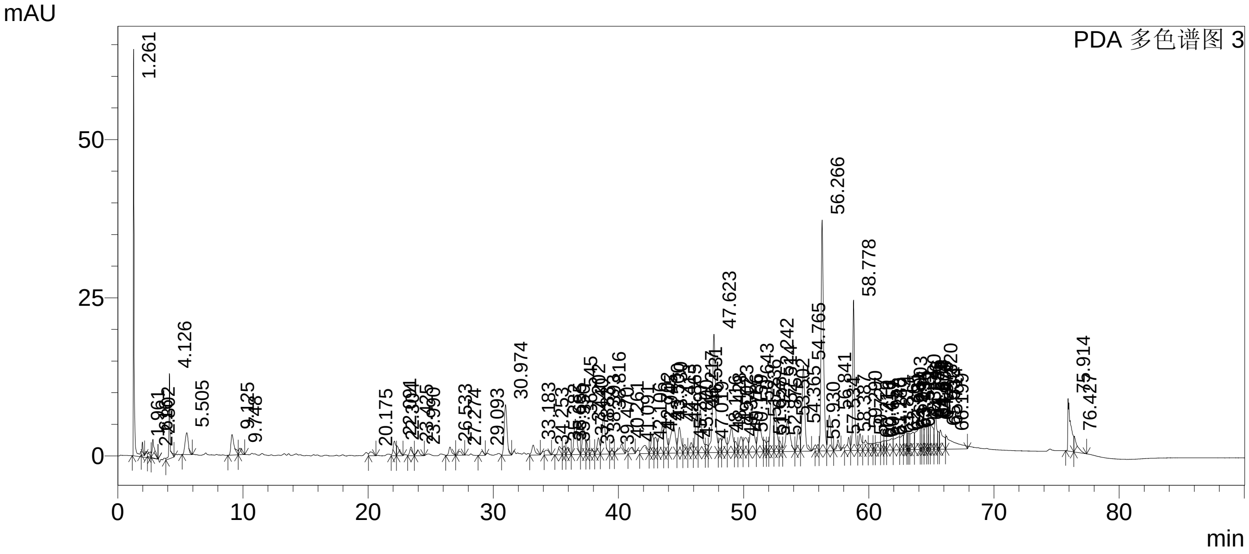

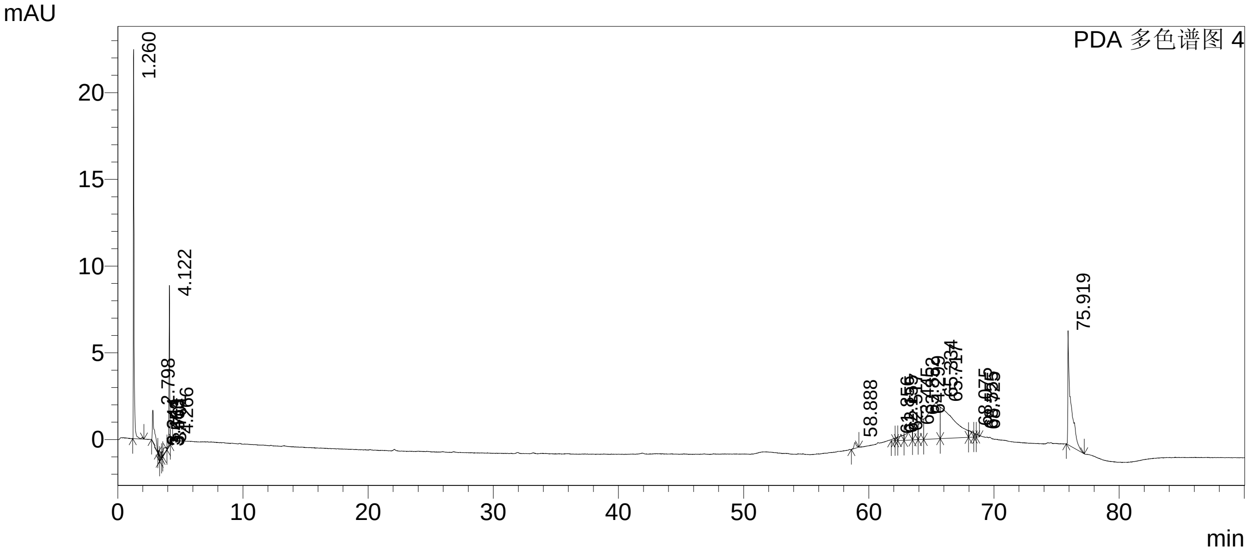

1 AD 2/
2 PDA 多色谱图 1/254nm 4nm
3 PDA 多色谱图 2/227nm 4nm
4 PDA 多色谱图 3/280nm 4nm
5 PDA 多色谱图 4/360nm 4nm

峰表

PDA Ch3 280nm 4nm

峰#	保留时间	面积	高度	面积 %	高度 %
1	1.261	263394	64250	4.525	15.043
2	1.961	13464	2049	0.231	0.480
3	2.616	11694	829	0.201	0.194
4	2.802	40363	2927	0.693	0.685
5	4.126	75832	13447	1.303	3.148
6	5.505	61633	3459	1.059	0.810
7	9.125	62257	3257	1.070	0.763
8	9.748	14184	1044	0.244	0.244
9	20.175	13652	617	0.235	0.144
10	22.091	34847	2346	0.599	0.549
11	22.304	23879	1585	0.410	0.371
12	23.425	21027	1385	0.361	0.324
13	23.990	16703	856	0.287	0.200
14	26.533	21305	1345	0.366	0.315
15	27.274	11629	627	0.200	0.147
16	29.093	11406	684	0.196	0.160
17	30.974	125551	8000	2.157	1.873
18	33.183	28660	1578	0.492	0.369
19	34.253	17256	766	0.296	0.179
20	35.282	24983	1318	0.429	0.309
21	35.655	19761	1401	0.339	0.328
22	35.950	18864	1198	0.324	0.281
23	36.545	93563	5597	1.607	1.310
24	37.202	66048	4359	1.135	1.021
25	37.440	23291	2064	0.400	0.483
26	37.883	14122	719	0.243	0.168
27	38.393	39475	2601	0.678	0.609
28	38.816	109405	6188	1.880	1.449
29	39.470	10969	616	0.188	0.144
30	40.261	42285	1600	0.726	0.375
31	41.091	24391	1029	0.419	0.241
32	42.106	40940	1290	0.703	0.302
33	42.702	43654	2876	0.750	0.673
34	42.945	39147	2563	0.673	0.600
35	43.500	92557	4328	1.590	1.013
36	43.730	69602	4537	1.196	1.062
37	44.413	115261	4107	1.980	0.962
38	44.865	70502	4086	1.211	0.957
39	45.297	23634	1324	0.406	0.310
40	45.800	28254	1622	0.485	0.380
41	46.217	88539	6177	1.521	1.446
42	46.531	126140	6797	2.167	1.592
43	47.019	16953	1294	0.291	0.303
44	47.623	313211	18707	5.381	4.380
45	48.116	32536	2242	0.559	0.525
46	48.428	54025	2653	0.928	0.621
47	49.023	83063	3906	1.427	0.915
48	49.347	24125	1699	0.414	0.398
49	49.786	43905	2453	0.754	0.574
50	50.159	52864	2279	0.908	0.533
51	50.643	137424	7287	2.361	1.706
52	51.236	100668	4713	1.729	1.103
53	51.658	24938	1878	0.428	0.440
54	51.927	18183	1697	0.312	0.397
55	52.242	166062	11169	2.853	2.615
56	52.514	108891	6849	1.871	1.604
57	52.875	20452	1691	0.351	0.396
58	53.502	118101	4778	2.029	1.119
59	54.365	52855	3549	0.908	0.831
60	54.765	207748	13554	3.569	3.173
61	55.930	13181	1021	0.226	0.239
62	56.266	499225	36505	8.576	8.547
63	56.841	85365	5648	1.467	1.322
64	57.534	25886	1714	0.445	0.401
65	58.387	31446	2114	0.540	0.495
66	58.778	233384	23759	4.009	5.563
67	59.290	39087	2649	0.672	0.620

峰#	保留时间	面积	高度	面积 %	高度 %
68	59.727	32996	1632	0.567	0.382
69	60.219	23715	1084	0.407	0.254
70	60.416	11704	1080	0.201	0.253
71	60.651	30975	1231	0.532	0.288
72	61.148	21906	1454	0.376	0.340
73	61.280	18579	1470	0.319	0.344
74	61.814	50956	1785	0.875	0.418
75	62.365	58947	2060	1.013	0.482
76	62.681	38318	2308	0.658	0.540
77	62.903	55920	4804	0.961	1.125
78	63.094	14749	2593	0.253	0.607
79	63.196	19449	2556	0.334	0.598
80	63.511	58761	2784	1.009	0.652
81	63.980	93316	5126	1.603	1.200
82	64.176	28116	3814	0.483	0.893
83	64.286	39595	4190	0.680	0.981
84	64.573	47432	4191	0.815	0.981
85	64.674	30867	4174	0.530	0.977
86	64.808	34671	4079	0.596	0.955
87	64.976	63544	3858	1.092	0.903
88	65.320	76422	6810	1.313	1.594
89	65.536	22709	2854	0.390	0.668
90	65.714	65314	3051	1.122	0.714
91	66.199	104563	2081	1.796	0.487
92	75.914	136360	8281	2.343	1.939
93	76.427	43313	2500	0.744	0.585
总计		5820897	427106	100.000	100.000
