# Supplementary material for: Paclitaxel-Containing Extract Exerts Anti-Cancer Activity through Oral Administration in A549-Xenografted BALB/C Nude Mice: Synergistic Effect between Paclitaxel and Flavonoids or Lignoids
Source: Evid Based Complement Alternat Med. 2022 Apr 25;2022:3648175. doi: 10.1155/2022/3648175 (PMC9060980; doi:10.1155/2022/3648175)
Supplement: Supplementary Materials — Data are available in the supplement file. [file 3648175.f1.zip › 3648175.f1/Figure 2-4 and Table 3 in vivo experiment data (1).pdf]

Date	Day 1				Day 4				Day 7				Day 10			
		tumor volume=Lenth×Width^2/2				tumor volume=Lenth×Width^2/2				tumor volume=Lenth×Width^2/2				tumor volume=Lenth×Width^2/2		
Animal No.	Weigh g	length mm	width mm	tumor volume mm^3	Weigh g	length mm	width mm	tumor volume mm^3	Weigh g	length mm	width mm	tumor volume mm^3	Weigh g	length mm	width mm	tumor volume mm^3
801	25.2	5.52	4.86	65.19	25.8	6.17	4.72	68.73	25.6	6.06	5.48	90.99	25.4	6.57	5.81	110.89
802	25.6	4.53	4.48	45.46	26.2	6.18	5.37	89.11	26.5	6.17	5.29	86.33	26.4	6.24	6.09	115.71
803	25.8	4.65	4.69	51.14	25.9	5.73	4.82	66.56	25.9	6.21	5.4	90.54	26.9	7.06	5.59	110.31
804	27.6	4.38	4.05	35.92	27.3	5.37	4.85	63.16	26.9	5.74	4.88	68.35	27.2	6.13	5.69	99.23
805	26.2	6.34	5.25	87.37	26.8	7.5	6.4	153.60	26.2	8.7	6.18	166.14	26.9	8.31	7.27	219.60
806	26.6	4.84	4.32	45.16	27.5	7.4	6.08	136.78	28	8.71	5.68	140.50	28	9.13	7.78	276.31
301	26.7	6.63	6.16	125.79	26.4	7.39	6.23	143.41	27	7.75	6.79	178.65	26.6	7.75	7.61	224.41
302	26.4	7.44	5.14	98.28	27.3	6.94	6.19	132.96	26.5	6.5	6.47	136.05	27.6	7.77	6.97	188.74
303	27	7.68	5.92	134.58	26.5	7.7	5.85	131.76	25	9.23	6.34	185.50	25.1	10.05	7.16	257.61
304	26.3	5.89	5.5	89.09	26.4	6.21	6.24	120.90	26.3	7.05	6.26	138.14	26.4	7.95	6.71	178.97
305	27.5	5.98	6.49	125.94	27.4	6.64	6.53	141.57	28.3	6.81	6.03	123.81	28.7	7.13	6.81	165.33
306	27	4.9	4.9	58.82	26.4	8.14	5.36	116.93	25.7	8.03	5.73	131.82	26.4	8.67	7.08	217.30
Mean	26.49			80.23	26.66			113.79	26.49			128.07	26.80			180.37
SD	0.73			35.04	0.59			32.96	0.96			37.84	1.01			60.91

Date	Day 13				Day 16				Day 19				Day 22			
		tumor volume=Lenth×Width^2/2				tumor volume=Lenth×Width^2/2				tumor volume=Lenth×Width^2/2				tumor volume=Lenth×Width^2/2		
Animal No.	Weigh g	length mm	width mm	tumor volume mm^3	Weigh g	length mm	width mm	tumor volume mm^3	Weigh g	length mm	width mm	tumor volume mm^3	Weigh g	length mm	width mm	tumor volume mm^3
801	25.3	7.53	6.59	163.51	26.5	7.2	6.01	130.03	26.2	8.04	6.21	155.03	25.9	8.4	6.38	170.96
802	26.7	6	6.56	129.10	24.9	6.25	5.87	107.68	24.6	7.32	7.2	189.73	25.5	7.49	7.5	210.66
803	26.4	7.22	6.56	155.35	26.5	7.1	6.06	130.37	26.4	8.09	6.52	171.95	26.7	8.46	6.7	189.88
804	27.4	7.29	6.86	171.53	29.1	6.33	6.15	119.71	28.8	7.22	5.84	123.12	28.3	7.04	5.93	123.78
805	27	9.91	8.22	334.80	27.2	9.53	7.16	244.28	26.9	8.95	7.11	226.22	27.3	9.76	7.58	280.39
806	27.5	8.36	7.11	211.31	27.4	9.93	7.34	267.49	27.3	10.2	6.2	196.04	27.1	11.13	6.39	227.23
301	26.6	8.17	8.17	272.67	26.9	7.45	6.37	151.15	26.7	7.19	6.13	135.09	26.7	6.51	6.25	127.15
302	26.4	8.05	7.19	208.08	27.3	8.74	7.56	249.76	27.5	8.95	8.67	336.38	28	9.73	7.24	255.01
303	25.7	10.1	7.32	270.59	25.8	10.12	6.45	210.51	24.7	10.46	7.31	279.47	26	11.24	6.6	244.81
304	26.4	7.81	7.02	192.44	25.1	7.99	7.49	224.12	25.8	9.37	8.35	326.65	26.4	10.55	8.92	419.71
305	27.8	8.71	8.04	281.51	28.9	7.35	6.94	177.00	28.7	7.85	6.97	190.68	28	7.59	7.34	204.46
306	26	8.71	6.88	206.14	26.8	9.65	6	173.70	26.1	10.23	6.96	247.78	25.9	10.7	6.97	259.91
Mean	26.60			216.42	26.87			182.15	26.64			214.85	26.82			226.16
SD	0.74			61.27				55.74	1.33			70.34	0.93			78.67

Date	Day 25				Day 28				Day 31				Day 34			
		tumor volume=Lenth×Width^2/2				tumor volume=Lenth×Width^2/2				tumor volume=Lenth×Width^2/2				tumor volume=Lenth×Width^2/2		
Animal No.	Weigh g	length mm	width mm	tumor volume mm^3	Weigh g	length mm	width mm	tumor volume mm^3	Weigh g	length mm	width mm	tumor volume mm^3	Weigh g	length mm	width mm	tumor volume mm^3
801	24.6	7.88	6.38	160.38	23.7	8.83	5.98	157.88	25.5	9.54	6.92	228.42	25.3	9.58	6.91	228.71
802	25.5	7.49	7.5	210.66	25	7.42	7.41	203.71	25.9	7.65	7.55	218.03	25.9	8.73	8.41	308.73
803	26.7	8.46	6.7	189.88	26.1	9.47	6.71	213.19	26.4	9.55	7.52	270.03	27.2	10.1	7.92	316.77
804	28.3	7.04	5.93	123.78	28.8	7.65	6.52	162.60	28.9	8.63	7.06	215.08	29.8	9.22	6.58	199.60
805	27.3	9.76	7.58	280.39	26.1	10.83	7.62	314.42	26.8	12.1	8.7	457.92	26.9	12.55	8.97	504.89
806	27.1	11.13	6.39	227.23	28	10.43	6.62	228.54	27.2	11.11	8.03	358.19	27.9	11.06	8.01	354.81
301	26.7	6.51	6.25	127.15	26.4	4.5	4.79	51.62	26.9	4.5	4.6	47.61	26.2	4	4.51	40.68
302	28.5	9.71	7.33	260.85	28	9.92	7.77	299.45	28.6	10.59	8.12	349.12	28	10.91	8.3	375.79
303	25.5	11.39	7.67	335.03	24.7	10.65	6.18	203.37	24.3	11.93	7.54	339.12	23.8	12.09	7.73	361.21
304	23.6	9.96	9	403.38	24.4	10.9	9.78	521.28	25.6	11.77	10.31	625.55	25.6	12.04	10.83	706.08
305	27.1	7.75	7.35	209.34	27.3	8.49	6.66	188.29	27.5	8.38	7.46	233.18	26.7	9.68	8.41	342.32
306	25.6	11.74	6.93	281.91	25.5	11.02	7.18	284.05	26	12.53	7.67	368.56	25.8	12.17	7.77	367.37
Mean	26.38			234.16	26.17			235.70	26.63			309.24	26.59			342.25
SD	1.46			83.23	1.60			114.54	1.31			144.62	1.54			161.54

Date	Day 1				Day 4			
		tumor volume=Lenth×Width^2/2				tumor volume=Lenth×Width^2/2		
Animal No.	Weigh g	length mm	width mm	tumor volume mm^3	Weigh g	length mm	width mm	tumor volume mm^3
1401	28	5.59	4.32	52.16	27.5	7.79	5.04	98.94
1402	25.5	4.94	4.46	49.13	25.4	5.64	4.82	65.52
1403	26.4	6.12	4.01	49.21	25.8	6.88	3.83	50.46
1404	27.6	4.8	4.37	45.83	27.4	6.11	5.45	90.74
1405	25.6	4.2	4.14	35.99	25.4	5.92	5.79	99.23
1406	25.6	6.33	5.69	102.47	26.1	5.19	4.08	43.20
101	24.8	6.33	5.69	102.47	25.6	6.76	5.03	85.52
102	29.2	4.77	5.8	80.23	29.5	4.99	4.91	60.15
103	26.9	5.78	5.32	81.79	26.4	7.95	5.63	126.00
104	27.1	7.62	6.77	174.62	27	7.65	6.29	151.33
105	24.5	6.57	5.72	107.48	24.4	6.15	5.75	101.67
1201	28.3	6.16	5.29	86.19	27.4	6.43	5.22	87.60
	26.63			80.63	26.49			88.36
SD	1.47			38.77	1.35			31.04

Date	Day 7				Day 10			
		tumor volume=Lenth×Width^2/2				tumor volume=Lenth×Width^2/2		
Animal No.	Weigh g	length mm	width mm	tumor volume mm^3	Weigh g	length mm	width mm	tumor volume mm^3
1401	26.3	7.76	5.15	102.91	25.8	9.42	6.23	182.81
1402	25.4	6.13	5.28	85.45	23.4	7.29	5.94	128.61
1403	26.2	7.04	4.31	65.39	26.6	7.67	5	95.88
1404	28.2	6.22	5.4	90.69	27.8	6.73	5.38	97.40
1405	25	7.11	6.04	129.69	24.1	6.83	6.14	128.74
1406	25.9	8	5.71	130.42	25.2	7.45	5.32	105.43
101	25.5	6.95	4.78	79.40	23.4	9.56	5.82	161.91
102	29.2	5.19	5.46	77.36	27.1	5.44	5.17	72.70
103	26.9	8.87	6.38	180.52	26.4	9.54	6.24	185.73
104	27.5	10.19	8.05	330.17	27.4	10.4	6.6	226.51
105	23.1	6.93	5.74	114.16	23.6	7.46	6.48	156.62
1201	27.6	6.11	5.73	100.30	26.5	7.27	6.22	140.63
	26.40			123.87	25.61			140.25
SD	1.62			72.03	1.62			44.63

Date	Day 13				Day 16			
		tumor volume=Lenth×Width^2/2				tumor volume=Lenth×Width2/2		
Animal No.	Weigh g	length mm	width mm	tumor volume mm^3	Weigh g	length mm	width mm	tumor volume mm^3
1401	28.3	9.03	7.7	267.69	27.8	9.32	6.33	186.72
1402	22.6	7.06	6.17	134.38	23.2	7.21	5.74	118.78
1403	26.1	7.98	4.95	97.76	26.2	7.7	5.01	96.64
1404	27.5	5.23	5.41	76.54	27.7	6.6	5.43	97.30
1405	24.9	6.91	6.87	163.07	25.5	7.11	5.91	124.17
1406	26.1	8.44	6.24	164.32	25.7	9.04	4.97	111.65
101	21.4	9.75	5.52	148.54	death in Day 14			
102	26.8	5.53	4.73	61.86	24	5.79	4.92	70.08
103	26.9	10.14	7.44	280.64	28	9.12	5.88	157.66
104	26.7	10.8	7.8	328.54	28.7	11.19	6.24	217.86
105	23.3	7.65	6.92	183.17	23.5	7.7	6.9	183.30
1201	25.3	7.07	6.19	135.45	25.9	6.58	5.03	83.24
	25.49			170.16	26.02			131.58
SD	2.09			82.98	1.89			47.93

Date	Day 19				Day 22			
		tumor volume=Lenth×Width^2/2				tumor volume=Lenth×Width2/2		
Animal No.	Weigh g	length mm	width mm	tumor volume mm^3	Weigh g	length mm	width mm	tumor volume mm^3
1401	28.2	10	7.84	307.33	28.2	9.91	6.86	233.18
1402	23.5	8.7	6.63	191.21	24.2	8.46	6.7	189.88
1403	27.1	8.03	5.35	114.92	27	9.41	5.79	157.73
1404	25.3	8.34	5.2	112.76	26.8	6.9	6.06	126.70
1405	25.8	7.53	6.98	183.43	25.9	5.07	6.64	111.77
1406	23.9	9.13	5.16	121.55	24.3	9.02	5.19	121.48
101	death in Day 14				death in Day 14			
102	25.5	6.69	5.26	92.55	25.2	5.09	4.31	47.28
103	28	10.41	6.22	201.37	24.3	10.55	6.69	236.09
104	28.9	11.91	7.58	342.15	27.4	12.32	7.48	344.65
105	23	7.65	6.18	146.09	death in Day 20			
1201	death in Day 17				death in Day 17			
	25.92			181.34	25.92			174.31
SD	2.08			84.36	1.50			87.98

Date	Day 25				Day 28			
		tumor volume=Lenth×Width^2/2				tumor volume=Lenth×Width2/2		
Animal No.	Weigh g	length mm	width mm	tumor volume mm^3	Weigh g	length mm	width mm	tumor volume mm^3
1401	28.4	10.63	7.84	326.69	26.90	12.06	8.08	393.68
1402	23.9	8.82	6.1	164.10	24.10	8.74	6.45	181.80
1403	27.5	8.68	6.02	157.28	26.40	9.15	6.29	181.01
1404	28.4	7.59	6.68	169.34	28.00	7.11	6.52	151.12
1405	25.8	7.85	6.66	174.10	25.00	8.30	7.04	205.68
1406	25.2	9.32	5.78	155.68	25.10	9.67	5.78	161.53
101	death in Day 14				death in Day 14			
102	death in Day 23			0.00	death in Day 23			
103	23.6	11	6.03	199.98	23.60	11.03	6.71	248.31
104	27.5	13.77	8.37	482.34	26.50	12.94	9.06	531.08
105	death in Day 20				death in Day 20			
1201	death in Day 17				death in Day 17			
	26.29			203.28	25.70			256.78
SD	1.93			133.40	1.50			135.36

Date	Day 31				Day 34			
		tumor volume=Lenth×Width^2/2				tumor volume=Lenth×Width2/2		
Animal No.	Weigh g	length mm	width mm	tumor volume mm^3	Weigh g	length mm	width mm	tumor volume mm^3
1401	27.3	11.6	8.38	407.30	27.00	12.53	8.89	495.14
1402	24.2	9.75	6.82	226.75	23.80	9.92	6.77	227.33
1403	27.5	10.98	7.06	273.64	27.70	10.41	7.28	7.26
1404	28.1	8.94	7.09	224.70	28.50	8.27	7.26	217.95
1405	26.1	8.65	8.09	283.06	25.90	8.48	8.35	295.62
1406	26.2	7.13	6.53	152.01	25.80	7.18	6.66	159.24
101					death in Day 14			
102	death in Day 23				death in Day 23			
103	21.7	10.96	6.36	221.66	21.20	10.94	6.37	221.96
104	27	11.08	8.49	399.32	27.10	11.88	9.04	485.43
105	death in Day 20				death in Day 20			
1201	death in Day 17				death in Day 17			
	26.01			273.56	25.88			263.74
SD	2.11			89.31	2.37			162.82

Day 19 Abdominal swelling was observed.

Date	Day 1				Day 4				Day 7				Day 10			
		tumor volume=Lenth×Width^2/2				tumor volume=Lenth×Width^2/2				tumor volume=Lenth×Width^2/2				tumor volume=Lenth×Width^2/2		
Animal No.	Weigh g	length mm	width mm	tumor volume mm^3	Weigh g	length mm	width mm	tumor volume mm^3	Weigh g	length mm	width mm	tumor volume mm^3	Weigh g	length mm	width mm	tumor volume mm^3
1501	26.6	5.79	5.41	84.73	23.8	7.85	5.7	127.52	18.9	4.51	3.99	35.90	18.8	6.9	5.39	100.23
1502	27.8	5.05	4.85	59.39	23.9	5.7	4.02	46.06	20.7	4.02	3.69	27.37	21.5	4.64	4.33	43.50
1503	26.6	7.1	5.11	92.70	22.8	7.12	5.67	114.45	Daeth in Day 5				Daeth in Day 5			
1504	23.9	6.16	4.58	64.61	21.9	5.59	5.27	77.63	18.9	4.82	4.59	50.77	20.1	6.14	5.06	78.60
1505	28.7	4.91	4.67	53.54	25.2	4.31	4.05	35.35	Daeth in Day 5				Daeth in Day 5			
1506	25.6	4.86	4.29	44.72	24	4.34	3.88	32.67	Daeth in Day 5				Daeth in Day 5			
401	26.3	5.73	4.93	69.63	21.8	5.17	4.59	54.46	Daeth in Day 5				Daeth in Day 5			
402	26.8	6.03	5.89	104.60	24.7	4.6	4.76	52.11	22.3	5.49	5.46	81.83	24.3	6.91	5.44	102.25
403	26.2	5.22	5.16	69.49	24.2	4.55	4.32	42.46	Daeth in Day 5				Daeth in Day 5			
404	26.6	8.56	6.59	185.87	23.4	7.35	5.7	119.40	19.7	7.29	4.31	67.71	19.9	9.55	5.45	141.83
405	25.8	4.82	4.68	52.78	21.2	4.19	3.98	33.19	Daeth in Day 5				Daeth in Day 5			
Mean	26.45			80.19	23.35			66.84	20.10			52.72	20.92			93.28
sd	1.22			39.45	1.28			36.77	1.44			22.37	2.12			35.98

Date	Day 13				Day 16				Day 19				Day 22			
		tumor volume=Lenth×Width^2/2				tumor volume=Lenth×Width2/2				tumor volume=Lenth×Width^2/2				tumor volume=Lenth×Width2/2		
Animal No.	Weigh g	length mm	width mm	tumor volume mm^3	Weigh g	length mm	width mm	tumor volume mm^3	Weigh g	length mm	width mm	tumor volume mm^3	Weigh g	length mm	width mm	tumor volume mm^3
1501	19.7	6.34	4.91	76.42	20.4	5.89	4.93	71.58	20.3	4.97	4.17	43.21	20.3	5.83	5.18	78.22
1502	22.4	5.07	4.23	45.36	22.2	3.85	3.32	21.22	21.6	4.71	3.53	29.35	20.3	4.2	4.09	35.13
1503	Daeth in Day 5				Daeth in Day 5				Daeth in Day 5				Daeth in Day 5			
1504	21.8	5.86	5.01	73.54	21.4	3.95	3.8	28.52	21.1	4.8	4.78	54.84	19	5.02	5.1	65.29
1505	Daeth in Day 5				Daeth in Day 5				Daeth in Day 5				Daeth in Day 5			
1506	Daeth in Day 5				Daeth in Day 5				Daeth in Day 5				Daeth in Day 5			
401	Daeth in Day 5				Daeth in Day 5				Daeth in Day 5				Daeth in Day 5			
402	25.8	4.85	5.84	82.71	26.8	4.15	4.55	42.96	26.8	4.15	4.55	42.96	27.1	5.46	5.85	93.43
403	Daeth in Day 5				Daeth in Day 5				Daeth in Day 5				Daeth in Day 5			
404	20.9	10.37	5.08	133.81	20.5	4.68	3.22	24.26	20.5	4.68	3.22	24.26	20.1	7.56	5.86	129.80
405	Daeth in Day 5				Daeth in Day 5				Daeth in Day 5				Daeth in Day 5			
Mean	22.12			82.37	22.26			37.71	22.06			38.92	21.36			80.37
sd	2.29			32.13	2.64			20.69	2.70			12.19	3.25			34.98

Date	Day 25				Day 28				Day 31				Day 34			
		tumor volume=Lenth×Width^2/2				tumor volume=Lenth×Width2/2				tumor volume=Lenth×Width^2/2				tumor volume=Lenth×Width2/2		
Animal No.	Weigh g	length mm	width mm	tumor volume mm^3	Weigh g	length mm	width mm	tumor volume mm^3	Weigh g	length mm	width mm	tumor volume mm^3	Weigh g	length mm	width mm	tumor volume mm^3
1501	20.5	6.81	5.34	97.10	20.9	5.55	4.11	46.88	21.9	6.84	4.74	76.84	21.6	6.46	4.78	73.80
1502	20.3	3.69	3.86	27.49	20.6	3.76	2.99	16.81	21.5	3.68	3.43	21.65	22	3.49	3.18	17.65
1503	Daeth in Day 5				Daeth in Day 5				Daeth in Day 5				Daeth in Day 5			
1504	19.4	4.33	4.5	43.84	18.7	4.49	4.17	39.04	19	5.29	4.56	55.00	18.5	4.12	4.02	33.29
1505	Daeth in Day 5				Daeth in Day 5				Daeth in Day 5				Daeth in Day 5			
1506	Daeth in Day 5				Daeth in Day 5				Daeth in Day 5				Daeth in Day 5			
401	Daeth in Day 5				Daeth in Day 5				Daeth in Day 5				Daeth in Day 5			
402	27.3	5.52	5.16	73.49	26.4	8.63	6.52	183.43	25.9	8.43	5.65	134.55	26	8.49	6.91	202.69
403	Daeth in Day 5				Daeth in Day 5				Daeth in Day 5				Daeth in Day 5			
404	21.3	9.11	4.81	105.38	19.6	9.82	5.33	139.49	20	9.62	6.29	190.30	19.7	9.4	6.78	216.05
405	Daeth in Day 5				Daeth in Day 5				Daeth in Day 5				Daeth in Day 5			
Mean	21.76			69.46	21.24			85.13	21.66			95.67	21.56			108.70
sd	3.17			33.50	3.01			72.24	2.64			67.00	2.86			94.28
