# Supplementary material for: Paclitaxel-Containing Extract Exerts Anti-Cancer Activity through Oral Administration in A549-Xenografted BALB/C Nude Mice: Synergistic Effect between Paclitaxel and Flavonoids or Lignoids
Source: Evid Based Complement Alternat Med. 2022 Apr 25;2022:3648175. doi: 10.1155/2022/3648175 (PMC9060980; doi:10.1155/2022/3648175)
Supplement: Supplementary Materials — Data are available in the supplement file. [file 3648175.f1.zip › 3648175.f1/Figure 2-4 and Table 3 in vivo experiment data-2 (1).pdf]

Date	Day 1				Day 4				Day 7				Day 10			
		tumor volume=Lenth×Width^2/2				tumor volume=Lenth×Width^2/2				tumor volume=Lenth×Width^2/2				tumor volume=Lenth×Width^2/2		
Animal No.	Weigh g	length mm	width mm	tumor volume mm^3	Weigh g	length mm	width mm	tumor volume mm^3	Weigh g	length mm	width mm	tumor volume mm^3	Weigh g	length mm	width mm	tumor volume mm^3
1601	26.5	5.28	5.77	87.89	24.7	8.97	5.84	152.96	23.5	5.52	5.18	74.06	22.9	6.53	5.46	97.33
1602	28.2	4.48	4.09	37.47	27.8	5.21	4.32	48.62	23.6	5.82	4.24	52.31	27.7	5.53	4.96	68.02
1603	25.3	4.49	4.09	37.55	23.7	3.91	3.6	25.34	22.5	4.17	4.14	35.74	21.4	4.31	4.61	45.80
1604	26.9	7.19	5.05	91.68	24.2	7.33	5.89	127.15	21.1	5.92	5.16	78.81	20.4	4.7	4.17	40.86
1605	26.1	4.92	5.32	69.62	25.5	5.44	5.56	84.08	20.1	4.03	4.01	32.40	20.1	4.7	4.17	40.86
601	26.8	9.22	5.8	155.08	24.9	8.89	5.39	129.14	23.4	9.66	6.55	207.22	23.4	10.13	6.77	232.14
602	26.3	5.04	4.41	49.01	26.2	6.25	4.07	51.77	26.3	7.73	4.79	88.68	22.9	7.98	6.51	169.10
603	26.5	5.97	4.89	71.38	25	6.05	4.45	59.90	25	6.23	4.91	75.10	23	6.67	5.21	90.53
604	26.6	6.18	5.68	99.69	25	6.28	5.62	99.18	24.7	6.64	5.89	115.18	23.7	8.4	6.89	199.38
605	26.7	7.33	5.5	110.87	26	7.88	5.43	116.17	25.4	8.11	5.73	133.14	25.4	8.2	5.68	132.28
606	25	6.2	5.59	96.87	24.3	7.15	5.87	123.18	24.6	6.87	5.35	98.32	23.8	8.08	6.52	171.74
	26.45			82.47	25.21			92.50	23.65			90.09	23.15			117.10
sd	0.84			34.85	1.14			41.20	1.86			49.59	2.16			67.93

Date	Day 13				Day 16				Day 19				Day 22			
		tumor volume=Lenth×Width^2/2				tumor volume=Lenth×Width2/2				tumor volume=Lenth×Width^2/2				tumor volume=Lenth×Width2/2		
Animal No.	Weigh g	length mm	width mm	tumor volume mm^3	Weigh g	length mm	width mm	tumor volume mm^3	Weigh g	length mm	width mm	tumor volume mm^3	Weigh g	length mm	width mm	tumor volume mm^3
1601	23.8	6.46	5.47	96.64	24	6.45	5.75	106.63	24.6	6.97	6.59	151.35	25	7.6	5.97	135.44
1602	23.1	4.8	4.53	49.25	22.8	5.05	4.58	52.97	22.7	4.32	4.06	35.60	22.2	4.6	4.32	42.92
1603	21.5	3.99	4.55	41.30	21.4	3.99	3.59	25.71	21.1	4	4.2	35.28	21.5	3.69	3.66	24.71
1604	21	5.65	5.07	72.62	22.5	7.69	4.86	90.82	22	5.65	5.03	71.48	21.4	6.29	4.8	72.46
1605	20.6	5.15	4.44	50.76	20.3	4.85	4.33	45.47	20.6	4.6	4.81	53.21	19.5	4.46	4.38	42.78
601	25.1	10.7	7.36	289.81	27.6	9.9	6.43	204.66	24.1	11.3	7.56	322.92	25.3	10.37	7.15	265.07
602	24.2	7.19	6.29	142.23	24.8	8.14	6.11	151.94	22.5	9.39	7.17	241.36	22.4	9.05	7.32	242.46
603	22.5	7.02	5.51	106.56	22.7	6.91	4.63	74.06	21.2	6.78	5	84.75	20.7	7.29	5.38	105.50
604	24.1	7.78	6.91	185.74	25	7.71	7.03	190.52	22.7	8.03	7.43	221.65	23.1	8.36	7.33	224.59
605	25.9	8.79	6.91	209.85	27.7	8.21	5.83	139.52	25	9.52	6.36	192.54	25.4	9.56	6.75	217.79
606	23.5	7.78	5.96	138.18	23.7	6.54	5.38	94.65	20.7	6.7	5.27	93.04	19.6	6.46	5.31	91.07
	23.21			125.72	23.86			106.99	22.47			136.65	22.37			133.16
sd	1.68			77.88	2.32			58.65	1.55			96.19	2.14			88.97

Date	Day 25				Day 28				Day 31				Day 34			
		tumor volume=Lenth×Width^2/2				tumor volume=Lenth×Width2/2				tumor volume=Lenth×Width^2/2				tumor volume=Lenth×Width2/2		
Animal No.	Weigh g	length mm	width mm	tumor volume mm^3	Weigh g	length mm	width mm	tumor volume mm^3	Weigh g	length mm	width mm	tumor volume mm^3	Weigh g	length mm	width mm	tumor volume mm^3
1601	25	7.07	5.65	112.85	24	6.45	5.75	106.63	25.2	7.39	6.27	145.26	25.5	7.76	7.07	193.94
1602	22.5	5.81	4.55	60.14	22.8	5.05	4.58	52.97	23.6	5.36	5.04	68.08	23.6	4.94	4.35	46.74
1603	21.5	4.1	3.5	25.11	21.4	3.99	3.59	25.71	21.8	4.36	4.39	42.01	21.7	4.01	4.05	32.89
1604	22.3	5.49	4.76	62.20	22.5	7.69	4.86	90.82	20.4	6.09	5.1	79.20	20	5.67	5.16	75.48
1605	19.7	4.6	4.9	55.22	20.3	4.85	4.33	45.47	20	5.06	5.12	66.32	20.5	4.12	4.72	45.89
601	25.9	11.16	7.59	321.45	27.36	9.9	6.43	204.66	26.7	10.54	7.72	314.08	27	11.58	8.6	428.23
602	22.4	9.34	7.65	273.30	24.8	8.14	6.11	151.94	23.5	10.43	7.8	317.28	22.8	10.58	8.19	354.83
603	20.6	7.85	5.82	132.95	22.47	6.91	4.63	74.06	20.6	8.8	6.42	181.35	20.7	9.1	6.16	172.65
604	23	9.18	7.97	291.56	25	7.71	7.03	190.52	23.8	9.55	7.7	283.11	24	9.73	7.73	290.70
605	24.8	9.99	7.4	273.53	27.7	8.21	5.83	139.52	25.2	10.81	7.84	332.22	21.2	10.2	7.47	284.58
606	17	6.07	4.29	55.86	23.7	6.54	5.38	94.65	Death in Day 29				Death in Day 29			
	22.25			151.29	23.82			106.99	23.08			182.89	22.70			192.59
sd	2.56			114.36	2.30			58.65	2.30			118.51	2.31			142.34

606 10-31死
