# Supplementary material for: Paclitaxel-Containing Extract Exerts Anti-Cancer Activity through Oral Administration in A549-Xenografted BALB/C Nude Mice: Synergistic Effect between Paclitaxel and Flavonoids or Lignoids
Source: Evid Based Complement Alternat Med. 2022 Apr 25;2022:3648175. doi: 10.1155/2022/3648175 (PMC9060980; doi:10.1155/2022/3648175)
Supplement: Supplementary Materials — Data are available in the supplement file. [file 3648175.f1.zip › 3648175.f1/Figure 2-4 and Table 3 in vivo experiment data-3 (1).pdf]

Date	Day 1				Day 4				Day 7				Day 10			
		tumor volume=Lenth×Width^2/2				tumor volume=Lenth×Width^2/2				tumor volume=Lenth×Width^2/2				tumor volume=Lenth×Width^2/2		
Animal No.	Weigh g	length mm	width mm	tumor volume mm^3	Weigh g	length mm	width mm	tumor volume mm^3	Weigh g	length mm	width mm	tumor volume mm^3	Weigh g	length mm	width mm	tumor volume mm^3
1001	25.6	5.33	5.47	79.74	26.6	6.16	5.41	90.15	26.6	6.56	5.85	112.25	27.8	7.74	7.4	211.92
1002	25.9	5.03	5.02	63.38	25.7	5.97	5.83	101.46	25.7	7.17	6.24	139.59	26.5	8.51	7.2	220.58
1003	27.3	6.78	5.49	102.17	24.8	6.73	5.22	91.69	24.3	6.7	5.7	108.84	23.8	8.49	6.18	162.13
1004	24.9	5.95	4.73	66.56	25.8	8.31	5.55	127.98	25.4	9.41	5.42	138.22	25.1	10.34	6.18	197.45
1005	27.2	5.18	4.48	51.98	26.6	5.66	5.02	71.32	26.6	5.42	4.92	65.60	25.8	6.11	5.47	91.41
1006	26.3	5.82	5.11	75.99	26	6.93	5.24	95.14	26.3	7.66	5.65	122.26	26.5	7.84	6.71	176.49
1204	27.6	5.45	5.3	76.55	26.6	6.93	5.24	95.14	25.9	7.3	5.55	112.43	25.5	7.67	6.14	144.58
902	27.7	6.12	4.22	54.49	27.7	6.95	5.1	90.38	27.3	7.89	6.84	184.57	27.4	8.71	7.31	232.71
903	26.5	5.79	5.1	75.30	27.5	6.73	5.13	88.56	27.2	7.24	5.44	107.13	27.5	9.29	6.07	171.14
904	26.5	5.79	5.1	75.30	25.8	6.28	5.76	104.18	26.3	7.07	7.01	173.71	27.3	8.38	7.33	225.12
905	26.1	10.31	5.89	178.84	26.5	10.95	5.83	186.09	25.3	10.35	6.93	248.53	26	11.76	7.83	360.50
	26.51			81.85	26.33			103.83	26.08			137.56	26.29			199.46
sd	0.88			34.91	0.83			30.52	0.88			49.27	1.21			67.61

Date	Day 13				Day 16				Day 19				Day 22			
		tumor volume=Lenth×Width^2/2				tumor volume=Lenth×Width2/2				tumor volume=Lenth×Width^2/2				tumor volume=Lenth×Width2/2		
Animal No.	Weigh g	length mm	width mm	tumor volume mm^3	Weigh g	length mm	width mm	tumor volume mm^3	Weigh g	length mm	width mm	tumor volume mm^3	Weigh g	length mm	width mm	tumor volume mm^3
1001	24.3	6.98	6.48	146.55	23.2	6.82	5.74	112.35	Death in Day 17				Death in Day 17			
1002	27	8.3	7.8	252.49	28.2	8.86	8.37	310.35	27.4	8.62	8.31	297.63	27.9	9.56	9.41	423.26
1003	23.2	8.7	6.03	158.17	23.6	8.13	6.85	190.74	23.7	8.8	6.51	186.47	23.6	8.76	6.82	203.72
1004	24.4	10.55	6.5	222.87	24.3	10.59	7.01	260.20	23.5	11.34	7.26	298.85	23.5	11.17	7.22	291.14
1005	26.7	6.36	6.2	122.24	27.3	5.62	5.09	72.80	26.7	6.81	6.04	124.22	27.4	6.35	6.11	118.53
1006	27.2	7.45	5.41	109.02	25.9	8.21	6.05	150.25	25.8	8.35	6.32	166.76	25.3	9.35	7.03	231.04
1204	24.8	8.49	6.39	173.33	25.9	8.05	5.96	142.97	24.2	8.86	6.36	179.19	24	9.52	6.73	215.59
902	28.1	9.04	8.01	290.00	28.6	8.94	8.48	321.44	29.1	9.74	9.06	399.75	29.3	11.37	8.86	446.27
903	27.4	8.99	6.68	200.58	27.4	9.1	6.26	178.30	26.8	9.35	6.77	214.27	26.8	9.16	6.93	219.95
904	26.9	8.36	7.9	260.87	27.8	8.61	8.5	311.04	25.7	9.06	9.22	385.09	25.4	9.34	9.33	406.52
905	26	14.45	8.64	539.34	25.8	14.69	8.44	523.21	25.9	15.15	9.65	705.40	25	16.71	9.28	719.52
	26.00			225.04	26.18			233.97	25.88			295.76	25.82			327.55
sd	1.58			119.57	1.86			128.37	1.75			171.55	1.96			175.41

Date	Day 25				Day 28				Day 31				Day 34			
		tumor volume=Lenth×Width^2/2				tumor volume=Lenth×Width2/2				tumor volume=Lenth×Width^2/2				tumor volume=Lenth×Width2/2		
Animal No.	Weigh g	length mm	width mm	tumor volume mm^3	Weigh g	length mm	width mm	tumor volume mm^3	Weigh g	length mm	width mm	tumor volume mm^3	Weigh g	length mm	width mm	tumor volume mm^3
1001	Death in Day 17				Death in Day 17				Death in Day 17				Death in Day 17			
1002	28.2	9.87	9.5	445.38	27.2	10.26	10.18	531.63	27.6	11.23	10.13	576.19	27.8	11.48	10.57	641.30
1003	23.5	8.69	7.01	213.51	23.5	9.01	6.71	202.83	22.9	8.72	7.15	222.89	23.4	9.23	7.31	246.61
1004	24	12.02	6.1	223.63	24.6	12.28	7.19	317.41	25.2	12.9	7.97	409.71	25.4	12.61	7.9	393.50
1005	27.2	7.74	6.31	154.09	26.6	7.84	6.31	156.08	26.2	7.56	6.28	149.08	25.9	7.89	6.68	176.04
1006	25.9	8.06	7.28	213.58	25.4	9.07	7.9	283.03	25.2	9.5	8.3	327.23	25.2	9.84	8.42	348.81
1204	24.5	9.56	6.94	230.22	25.2	10.45	7.4	286.12	25	11.01	7.53	312.14	27	11.84	8.25	402.93
902	29.8	10.35	8.14	342.89	26.3	9.34	6.83	217.85	27.5	11.96	8	382.72	24.9	9.54	7.91	298.45
903	26.3	9.63	6.8	222.65	24.1	11.017	9.86	535.53	24.9	9.62	7.72	286.67	23.6	11.08	9.72	523.41
904	24.8	10.69	9.38	470.28	25.1	17.23	10.81	1006.72	23.9	10.31	9.72	487.04	25	17.29	11.07	1059.40
905	24.6	16.27	9.42	721.87	25.8	7.3	5.07	93.82	25.1	17.4	10.98	1048.88	25.4	8.17	5.4	119.12
	25.88			323.81	25.38			363.10	25.35			420.25	25.36			420.96
sd	2.02			175.50	1.14			268.53	1.45			253.01	1.34			272.73

	Day 1	Day 4	Day 7	Day 10	Day 13	Day 16	Day 19	Day 22	Day 25	Day 28	Day 31	Day 34
control group	80.23	113.79	128.07	180.37	216.42	182.15	214.85	226.16	234.16	235.70	309.24	342.25
PTX group	80.63	88.36	123.87	140.25	170.16	131.58	181.34	174.31	203.28	256.78	273.56	263.74
HDS-1 600mg/kg	80.19	66.84	52.72	93.28	82.37	37.71	38.92	80.37	69.46	85.13	95.67	108.70
HDS-1 200mg/kg	82.47	92.50	90.09	117.10	125.72	106.99	136.65	133.16	151.29	106.99	182.89	192.59
PTX+WAB 10+50mg/kg	81.85	103.83	137.56	199.46	225.04	233.97	295.76	327.55	323.81	363.10	384.06	420.96

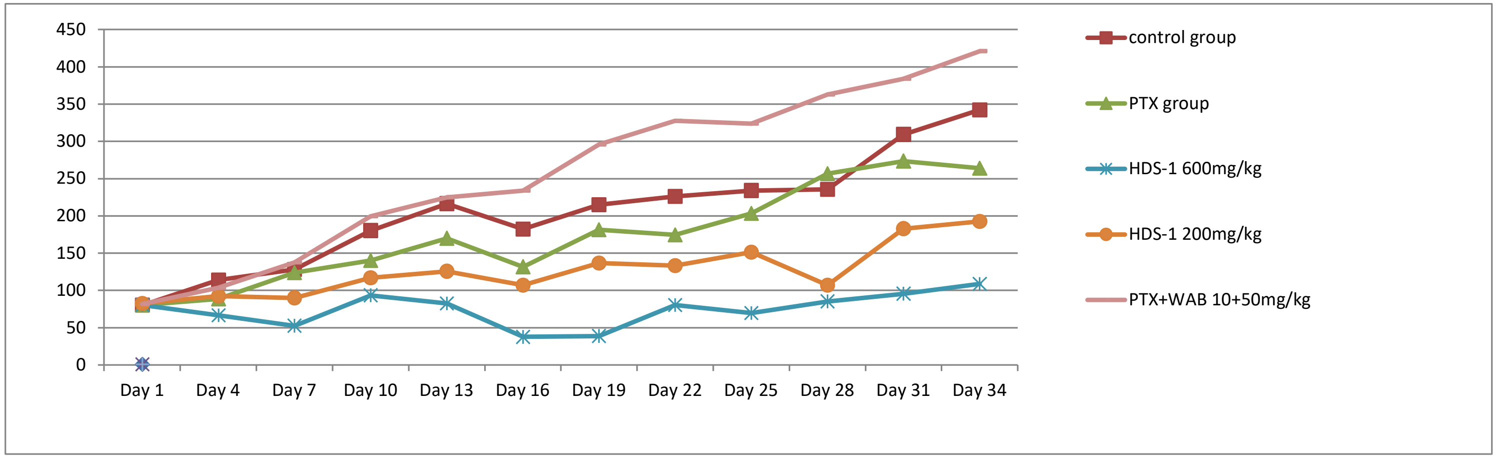

	Day 1	Day 4	Day 7	Day 10	Day 13	Day 16	Day 19	Day 22	Day 25	Day 28	Day 31	Day 34
control group	26.49	26.66	26.49	26.80	26.60	26.87	26.64	26.82	26.375	26.17	26.63333	26.59167
PTX group	26.63	26.49	26.40	25.61	25.49	26.02	25.92	25.92	26.2875	25.70	26.0125	25.875
HDS-1 600mg/kg	26.45	23.35	20.10	20.92	22.12	22.26	22.06	21.36	21.76	21.24	21.66	21.56
HDS-1 200mg/kg	26.45	25.21	23.65	23.15	23.21	23.86	22.47	22.37	22.2455	23.82	23.08	22.7
PTX+WAB 10+50mg/kg	26.51	26.33	26.08	26.29	26.00	26.18	25.88	25.82	25.88	25.38	25.35455	25.36

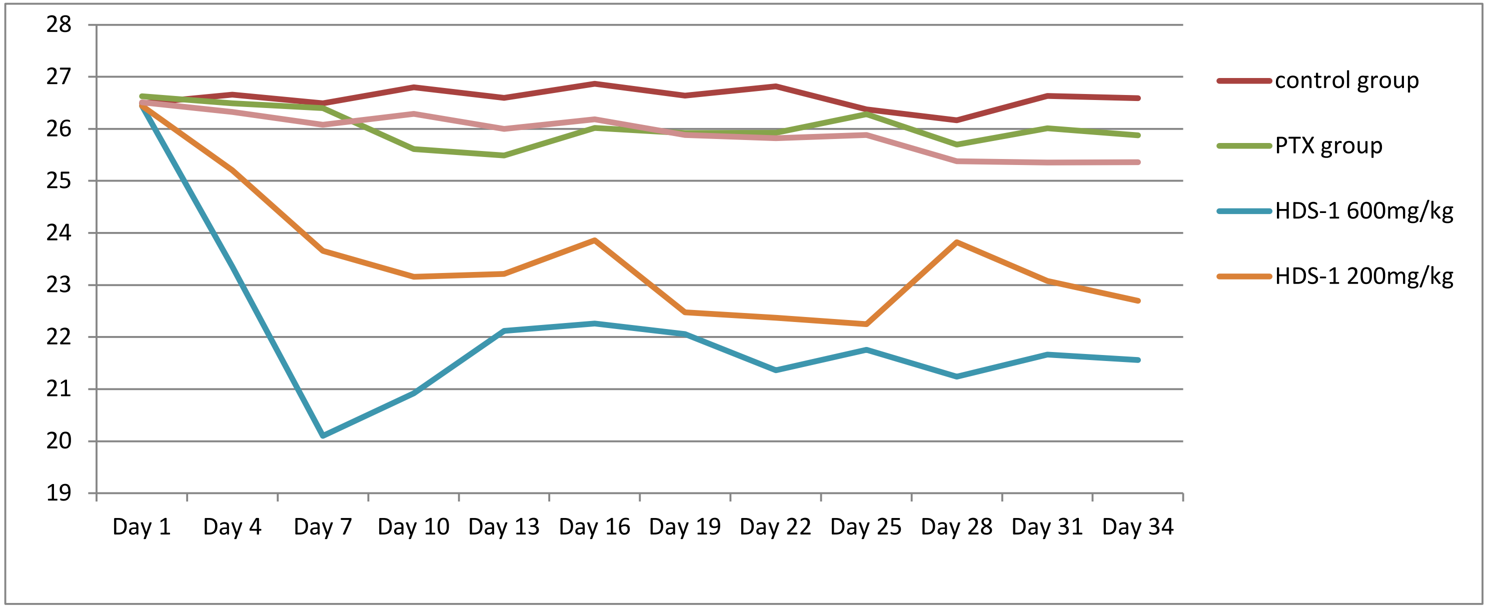

control		PTX		HDS-1 600mg/kg		HDS-1 200mg/kg		PTX+WAB 10+50mg/kg	
animal NO.	tumor weight	animal NO.	tumor weight	animal NO.	tumor weight	animal NO.	tumor weight	animal NO.	tumor weight
801	0.1283	1401	0.2469	1501	0.0353	1601	0.1426	1002	0.2973
802	0.1436	1402	0.1592	1502	0.0202	1602	0.0286	1003	0.1913
803	0.1495	1403	0.156	1504	0.017	1603	0.0243	1004	0.1569
804	0.1271	1404	0.1923	402	0.1213	1604	0.0461	1005	0.1152
805	0.2307	1405	0.1802	404	0.0944	1605	0.024	1006	0.2596
806	0.1865	1406	0.1183			601	0.2589	1204	0.1953
301	0.0474	103	0.1699			602	0.2126	902	0.3959
302	0.3012	104	0.486			603	0.0954	903	0.1666
303	0.2061					604	0.1856	904	0.3606
304	0.2934					605	0.2042	905	0.6613
305	0.2198							906	0.0975
306	0.2032								

Day 34

Group	Death Balb/c nude mice	total Balb/c nude	mortality %
control group	0	12	0.00%
PTX group	4	12	33.33%
HDS-1 600mg/kg	6	11	54.55%
HDS-1 200mg/kg	1	11	9.09%
PTX+WAB 10+50mg/kg	1	10	10.00%

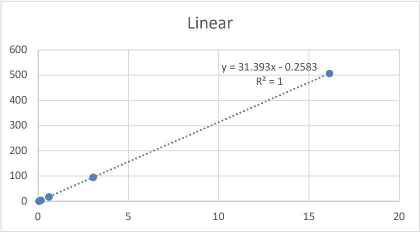

31.393 0.2583

sample ID	position		RT	Area	IS Area	Response	Conc.	%Dev	Adjusted concentration of PTX(ng/ml)	Mean	SD	
blank	1:A,4		0.95	66.069	11227.43	0.006						
1	1:A,5	1	0.94	278.132	5961.806	0.047	1.2	17.8				
5	1:A,6	5	0.94	1114.82	7074.832	0.158	4.7	-6.8				
20	1:A,7	20	0.95	3773.968	6317.027	0.597	18.5	-7.7				
100	1:A,8	100	0.95	22362.24	7338.162	3.047	95.4	-4.6				
500	1:B,1	500	0.95	95998.81	5949.175	16.136	506.3	1.3				
QC-1	1:B,2	1	0.95	252.442	4907.922	0.051	1.3	32.8				
QC-1	1:B,3	1	0.95	271.137	5957.47	0.046	1.1	14.2				
QC-1	1:B,4	1	0.95	274.88	6264.594	0.044	1.1	9.1				
QC-5	1:B,5	5	0.95	950.59	6100.294	0.156	4.6	-7.9				
QC-5	1:B,6	5	0.95	1034.488	5387.535	0.192	5.7	14.8				
QC-5	1:B,7	5	0.95	802.818	5521.642	0.145	4.3	-14.4				
QC-100	1:B,8	100	0.95	15929.17	5794.863	2.749	86	-14				
QC-100	1:C,1	100	0.95	10844.4	4996.328	2.17	67.9	-32.1				
QC-100	1:C,2	100	0.95	13694.71	4372.018	3.132	98.1	-1.9				
QC-400	1:C,3	400	0.95	55701.47	4040.554	13.786	432.5	8.1				
QC-400	1:C,4	400	0.95	63459.31	5497.869	11.543	362.1	-9.5				
QC-400	1:C,5	400	0.95	63786.89	5400.164	11.812	370.5	-7.4				
801	1:D,8	801	0.95	59.994	3699.901	0.016	0.2	-88.9				control group
802	1:E,1	802	0.95	34.003	3309.863	0.01	0	-98.2				
803	1:E,2	803	0.95	40.837	2722.401	0.015	0.2	-90.8				
804	1:E,3	804	0.94	75.466	3074.216	0.025	0.5	-75.8				
805	1:E,4	805	0.95	41.843	6350.717	0.007	NA					
806	1:E,5	806	0.95	93.896	2797.873	0.034	0.8	-61.7				
301	1:E,6	301	0.95	41.099	3093.435	0.013	0.1	-93.5				
302	1:E,7	302	0.95	54.761	3001.801	0.018	0.3	-85.7				
303	1:E,8	303	0.96	20.175	2872.624	0.007	NA					
304	1:F,1	304	0.94	5.508	2655.647	0.002	NA					
1401	1:F,2	1401	0.94	3388.414	2546.139	1.331	41.5	1283.1	55.33	201.48	112.17	PTX group
1402	1:F,3	1402	0.95	14609.04	3060.772	4.773	149.6	4885.3	199.47			
1403	1:F,4	1403	0.94	22673.68	2723.5	8.325	261.1	8602.6	348.13			
1404	1:F,5	1404	0.95	5466.526	1977.063	2.765	86.5	2783.9	115.33			
1405	1:F,6	1405	0.94	4526.103	2772.366	1.633	51	1598.9	68.00			
1406	1:F,7	1406	0.95	19211.25	2495.597	7.698	241.4	7946.3	321.87			
103	1:F,8	103	0.95	15488.99	2435.126	6.361	199.4	6546.7	265.87			
104	2:A,1	104	0.95	13082.95	2298.644	5.692	178.4	5846.6	237.87			
1501	2:A,2	1501	0.95	22392.16	2376.818	9.421	295.5	1310.8	394.00	314.60	124.99	HDS-1 600mg/kg
1502	2:A,3	1502	0.95	24193.16	2745.296	8.812	276.4	928.5	368.53			
1504	2:A,4	1504	0.95	24152.33	2745.166	8.798	275.9	5418.5	367.87			
402	2:A,5	402	0.95	8070.947	2630.364	3.068	96	1820.9	128.00			
1601	2:A,6	1601	0.94	21592	2272.796	9.5	298	4866.1	397.33	214.35	167.05	HDS-1 200mg/kg
1602	2:A,7	1602	0.94	12941.07	2659.053	4.867	152.5	2441.7	203.33			
1603	2:A,8	1603	0.94	24116.61	1912.746	12.608	395.5	6492.4	527.33			
1604	2:B,1	1604	0.95	6367.892	2612.151	2.438	76.2	1170.8	101.60			
1605	2:B,2	1605	0.95	19035.9	2019.223	9.427	295.7	4828	394.27			
601	2:B,3	601	0.95	8786.453	2413.962	3.64	114	1799.7	152.00			
602	2:B,4	602	0.94	2069.164	2528.176	0.818	25.4	323.5	33.87			
603	2:B,5	603	0.94	6641.55	2823.138	2.353	73.6	1126.2	98.13			
604	2:B,6	604	0.94	11003.24	2670.818	4.12	129.1	2050.9	172.13			
605	2:B,7	605	0.94	4988.275	3270.041	1.525	47.6	693.4	63.47			
1002	2:C,8	1002	0.95	3091.385	2843.247	1.087	33.8	323.1	45.07	26.07	11.14	PTX+WAB 10+50mg/kg
1003	2:D,1	1003	0.94	2294.35	2635.033	0.871	27	238.1	36.00			
1004	2:D,2	1004	0.94	1349.431	2852.51	0.473	14.6	82.1	19.47			
1005	2:D,3	1005	0.94	2165.762	2411.816	0.898	27.9	248.8	37.20			
1006	2:D,4	1006	0.94	1071.798	2403.91	0.446	13.7	71.4	18.27			
1204	2:D,5	1204	0.93	1836.956	2883.921	0.637	19.7	146.4	26.27			
902	2:D,6	902	0.93	738.221	2844.851	0.259	7.9	-1.7	10.53			
903	2:D,7	903	0.93	1247.75	2747.688	0.454	14	74.6	18.67			
904	2:D,8	904	0.93	1510.694	2682.953	0.563	17.4	117.4	23.20			
METHOD	1:A,1											
METHOD	1:A,1											
METHOD	1:A,1											
BLANK	1:A,2											
BLANK	1:A,3											
BLANK	1:A,4	BLANK	0.95	49.212	3086.672	0.016						
1	1:A,5	1	0.95	169.262	2152.928	0.079	1	0.5				
5	1:A,6	5	0.94	869.609	2911.747	0.299	5.4	8.2				
20	1:A,7	20	0.94	2746.884	2903.041	0.946	18.4	-8.1				
100	1:A,8	100	0.95	15497.2	3119.12	4.968	98.9	-1.1				
500	1:B,1	500	0.95	68878.38	2743.807	25.103	502.3	0.5				
QC-1	1:B,2	1	0.94	224.957	2185.819	0.103	1.5	49.1				
QC-1	1:B,3	1	0.91	115.543	1725.792	0.067	0.8	-22.9				
QC-1	1:B,4	1	0.94	180.932	2580.28	0.07	0.8	-16.6				
QC-5	1:B,5	5	0.94	801.19	2489.426	0.322	5.9	17.5				
QC-5	1:B,6	5	0.95	805.103	2379.664	0.338	6.2	24.1				
QC-5	1:B,7	5	0.95	734.38	2328.526	0.315	5.7	14.9				
QC-100	1:B,8	100	0.95	12246.27	2404.181	5.094	101.5	1.5				
QC-100	1:C,1	100	0.94	10717.35	2144.337	4.998	99.5	-0.5				
QC-100	1:C,2	100	0.95	12780.59	2145.93	5.956	118.7	18.7				
QC-400	1:C,3	400	0.95	56083.8	2514.853	22.301	446.1	11.5				
QC-400	1:C,4	400	0.95	60972.29	2655.056	22.965	459.4	14.9				
QC-400	1:C,5	400	0.95	64573.48	2872.862	22.477	449.6	12.4				

	Group1 (vehivle)											
Day 1 tumor size mm^3	65.1901	45.4595	51.1409	35.9215	87.3731	45.16301	125.79	98.2809	134.578	89.0863	125.939	58.8245
Day 34 tumor size mm^3	228.713	308.728	316.768	199.596	504.892	354.8054	40.6802	375.795	361.206	706.079	342.324	367.369
Inhibition Ratio= Tumor size Day 34/Tumor size Day 1	3.51	6.79	6.19	5.56	5.78	7.86	0.32	3.82	2.68	7.93	2.72	6.25
Mean (growth factor)	4.95											
Individual IR	29.1291	-37.186	-25.121	-12.242	-16.729	-58.695668	93.4673	22.7605	45.7826	-60.103	45.0921	-26.154

	Group 2 (PTX 10mg/k)											
Day 1 tumor size mm^3	52.1614	49.1323	49.2051	45.8326	35.9932	102.47			81.7939	174.623		
Day 34 tumor size mm^3	495.136	227.331	7.26	217.946	295.623	159.237			221.956	485.426		
Inhibition Ratio= Tumor size Day 34/Tumor size Day 1	9.49	4.63	0.15	4.76	8.21	1.55	#DIV/0!	#DIV/0!	2.71	2.78	#DIV/0!	#DIV/0!
Mean (growth factor)	13.43											
Individual IR	-91.749	6.53476	97.0195	3.94227	-65.912	68.6092			45.1846	43.8463		

	Group 3 HDS-1 600mg/k)											
Day 1 tumor size mm^3	84.7312	59.3943		64.6073				104.597		185.872		
Day 34 tumor size mm^3	73.8003	17.6461		33.2904				202.691		216.052		
Inhibition Ratio= Tumor size Day 34/Tumor size Day 1	0.87	0.30	#DIV/0!	0.52	#DIV/0!	#DIV/0!	#DIV/0!	1.94	#DIV/0!	1.16	#DIV/0!	#DIV/0!
Mean (growth factor)	80.67											
Individual IR	82.4057	93.9985		89.5913				60.8552		76.5199		

	Group 4HDS-1 200mg/k)											
Day 1 tumor size mm^3	87.8933	37.4709	37.5546	91.6815	69.6239	155.08	49.0092	71.3776	99.6908	110.866		
Day 34 tumor size mm^3	193.941	46.7386	32.887	75.4836	45.8935	428.228	354.833	172.653	290.698	284.585		
Inhibition Ratio= Tumor size Day 34/Tumor size Day 1	2.21	1.25	0.88	0.82	0.66	2.76	7.24	2.42	2.92	2.57	#DIV/0!	#DIV/0!
Mean (growth factor)	52.09											
Individual IR	55.4269	74.8036	82.3104	83.3686	86.6847	44.2203	-46.253	51.1383	41.0961	48.1475		

	Group 5 PTX+WAB 10+50mg/k)											
Day 1 tumor size mm^3		63.379	102.175	66.5594	51.9823	75.9862	76.5453	54.4937	75.299	75.299	178.838	
Day 34 tumor size mm^3		641.301	246.608	393.495	176.035	348.81	402.93	298.45	523.41	1059.4	119.119	
Inhibition Ratio= Tumor size Day 34/Tumor size Day 1	#DIV/0!	10.12	2.41	5.91	3.39	4.59	5.26	5.48	6.95	14.07	0.67	
Mean (growth factor)	-18.87											
Individual IR		-104.4	51.2449	-19.423	31.5928	7.27171	-6.3332	-10.632	-40.414	-184.2	86.5452	
