# Supplementary material for: Paclitaxel-Containing Extract Exerts Anti-Cancer Activity through Oral Administration in A549-Xenografted BALB/C Nude Mice: Synergistic Effect between Paclitaxel and Flavonoids or Lignoids
Source: Evid Based Complement Alternat Med. 2022 Apr 25;2022:3648175. doi: 10.1155/2022/3648175 (PMC9060980; doi:10.1155/2022/3648175)

PTX												
	AP-BL						BL-AP					
Time min	0	30	60	90	120		Time min	0	30	60	90	120
well-1 BL	0	0	0	0	0		well-1 AP	0	58.1786		404.8657	
well-2 BL	0	0	5.444271	0	0.034815		well-2 AP	0	64.7416	221.3719	412.9709	649.4581
well-3 BL	0	7.758878	6.584158	5.031508	8.934463		well-3 AP	0	91.58275	208.7763	394.3772	535.3417
Mean	0	2.586293	4.009476	1.677169	2.989759		Mean	0	71.50098	215.0741	404.0713	592.3999
SD	0	4.47959	3.518773	2.904942	5.148294		SD	0	17.6982	8.906434	9.322272	80.69248
slope	0.016901318						slope	5.057900278				
P_{app}	2.51508E-07						P_{app} BL-AP	7.52664E-05				
Efflux Ratio= Papp BL-AP/Papp AP-BL							299.26					

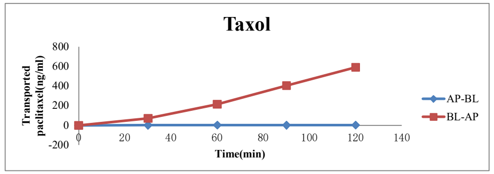

PTX+HDS-2 80ug/mL												
	AP-BL						BL-AP					
Time min	0	30	60	90	120		Time min	0	30	60	90	120
well-1 BL	0	51.40373	64.46845	69.24308	137.4186		well-1 AP	0	23.88043	98.56289	283.0036	253.3529
well-2 BL	0	50.67225	59.214	83.34232	147.4398		well-2 AP	0	26.5422	99.62147	204.7761	203.0089
well-3 BL	0	50.1846	70.25931	75.86904	114.121		well-3 AP	0		86.88968	187.9033	263.1588
Mean	0	50.75353	64.64725	76.15148	132.9931		Mean	0	25.21132	95.02468	225.2277	239.8402
SD	0	0.613615	5.524825	7.053862	17.09457		SD	0	1.882156	7.064971	50.74167	32.27146
slope	0.971280733						slope	2.265655839				
P_{app}	1.44536E-05						P_{app} BL-AP	3.37151E-05				
Efflux Ratio= Papp BL-AP/Papp AP-BL							2.33					

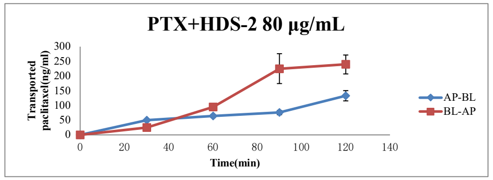

PTX+HDS-2 20ug/mL												
	AP-BL						BL-AP					
Time min	0	30	60	90	120		Time min	0	30	60	90	120
well-1 BL	0	8. 307486	11. 27171	16. 33894	26. 44117		well-1 AP	0	25. 70912	153. 8098	342. 9441	377. 5619
well-2 BL	0	11. 59914	11. 29609	18. 28344	32. 87818		well-2 AP	0	19. 00391	84. 15684	291. 2835	301. 8844
well-3 BL	0		13. 83798	18. 32002	29. 12935		well-3 AP	0	24. 69318	142. 4922	223. 8514	415. 3691
Mean	0	9.953313	12.13526	17.64747	29.4829		Mean	0	23.1354	126.8196	286.0263	364.9385
SD	0	2.327551	1.474649	1.133365	3.233036		SD	0	3.613857	37.37788	59.72015	57.78588
slope	0.222199846						slope	3.309226211				
P_{app}	3.30655E-06						P_{app} BL-AP	4.92444E-05				
Efflux Ratio= Papp BL-AP/Papp AP-BL							14.89					

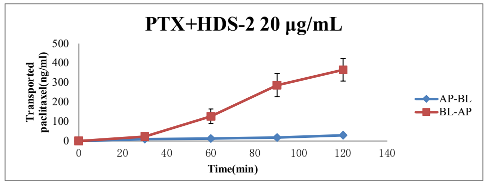

PTX+HDS-3 80ug/mL												
	AP-BL						BL-AP					
Time min	0	30	60	90	120		Time min	0	30	60	90	120
well-1 BL	0	2. 280593	30. 1357	27. 53655	61. 46531		well-1 AP	0	14. 1079	186. 9272	235. 6034	298. 7925
well-2 BL	0	0	42. 85811	53. 16951	66. 01787		well-2 AP	0	28. 72766	108. 3388	151. 246	195. 7142
well-3 BL	0	5. 666787	46. 92155	78. 2166	75. 62821		well-3 AP	0	17. 15368	145. 5744	188. 5909	277. 7551
Mean	0	2.649127	39.97179	52.97422	67.7038		Mean	0	19.99641	146.9468	191.8134	257.4206
SD	0	2.851312	8.757245	25.34059	7.230403		SD	0	7.713315	39.31215	42.27097	54.4647
slope	0.619108977						slope	2.288860484				
P_{app}	9.21293E-06						P_{app} BL-AP	3.40604E-05				
Efflux Ratio= Papp BL-AP/Papp AP-BL							3.70					

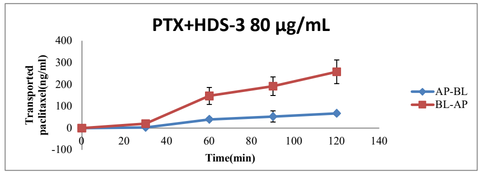

PTX+HDS-3 20ug/mL												
	AP-BL						BL-AP					
Time min	0	30	60	90	120		Time min	0	30	60	90	120
well-1 BL	0	0	0	7. 187139	0		well-1 AP	0	25. 07272	201. 71	253. 7026	299. 5091
well-2 BL	0	0	2. 605401	0	0		well-2 AP	0	39. 8179	142. 2509	210. 5653	156. 8428
well-3 BL	0	0	0	0	3. 437762		well-3 AP	0	42. 14703	161. 3838	208. 9887	319. 5665
Mean	0	0	0.868467	2.395713	1.145921		Mean	0	35.67921	168.4483	224.4188	258.6395
SD	0	0	1.504229	4.149497	1.984793		SD	0	9.259022	30.3525	25.37271	88.72707
slope	0.01562518						slope	2.353395224				
P_{app}	2.32518E-07						P_{app} BL-AP	3.50208E-05				
Efflux Ratio= Papp BL-AP/Papp AP-BL							150.62					

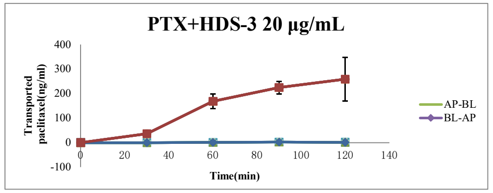

PTX+VRP 100μM												
	AP-BL						BL-AP					
Time min	0	30	60	90	120		Time min	0	30	60	90	120
1	0	9. 4972	37. 8944	83. 83768	279. 3899		1	0	62. 38698	199. 0149	293. 384	406. 339
2	0	10. 98609	32. 6054	87. 93673	272. 987		2	0	59. 5878	202. 3878	302. 8749	340. 8832
3	0	13. 08723	35. 9803	81. 73387	270. 5739		3	0	57. 988	187. 376	288. 4979	382. 4104
Mean	0	11.19017	35.49337	84.50276	274.3169		Mean	0	59.98759	196.2596	294.919	376.5442
SD	0	1.803695	2.677911	3.15446	4.555952		SD	0	2.226574	7.876067	7.31037	33.11985
slope	2.073154912						slope	3.293399189				
P_{app}	3.08505E-05						P_{app} BL-AP	4.90089E-05				
Efflux Ratio= Papp BL-AP/Papp AP-BL							1.59					

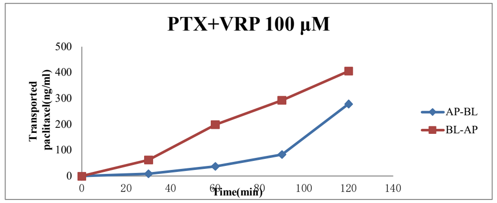

Supplement: Supplementary Materials — Data are available in the supplement file. [file 3648175.f1.zip › 3648175.f1/Figure 5 and Table 4 in Caco-2 permeability experiment (1).pdf]
