# Supplementary material for: Paclitaxel-Containing Extract Exerts Anti-Cancer Activity through Oral Administration in A549-Xenografted BALB/C Nude Mice: Synergistic Effect between Paclitaxel and Flavonoids or Lignoids
Source: Evid Based Complement Alternat Med. 2022 Apr 25;2022:3648175. doi: 10.1155/2022/3648175 (PMC9060980; doi:10.1155/2022/3648175)
Supplement: Supplementary Materials — Data are available in the supplement file. [file 3648175.f1.zip › 3648175.f1/Figure 6 MDCK-MDR1 experiment (1).pdf]

MDCK-MDR1

PTX					
BL-AP					
time(min	0	30	60	90	120
1	0	52.45521679	125.8676656	562.897	564.267
2	0	31.38463727	93.23546947	471.559	542.14
3	0	30.34066578	99.22546743	644.4472	724.775
	0	38.06017328	106.1095342	559.6344	610.394
	0	12.47739666	17.37117837	86.49027	99.6726
slope	5.807874781				
P_{app}	8.64267E-05				

PTX+VRP 100uM					
BL-AP					
time(min	0	30	60	90	120
1	0	35.6649	99.0622	174.6363357	284.965
2	0	35.4929	57.8172	105.4825694	237.612
3	0	40.805	106.157	157.9581377	247.382
	0	37.3209	87.6789	146.0256809	256.653
	0	3.01849	26.1031	36.08806578	25.0006
slope	2.07336976				
P_{app}	3.0854E-05				

PTX+HDS-2 80ug/mL					
BL-AP					
time(min	0	30	60	90	120
1	0	62.50489806	153.3184758	289.5578	312.31
2	0	44.52556401	128.4011834	243.9406	276.887
3	0	16.6102822	132.3626587	371.2498	502.519
	0	41.21358143	138.0274393	301.5828	363.905
	0	23.12586999	13.3897412	64.50082	121.343
slope	3.293933318				
P_{app}	4.90169E-05				

PTX+HDS-2 20ug/mL					
BL-AP					
time(min	0	30	60	90	120
1	0	18.5923		304.7388796	493.185
2	0	18.6364	100.283	321.6233394	442.614
3	0	32.7815	64.3057	361.9544578	481.05
	0	23.3367	82.2944	329.4388923	472.283
	0	8.17943	25.4398	29.3975806	26.4007
slope	4.16889619				
P_{app}	6.2037E-05				

PTX+HDS-3 80ug/mL					
BL-AP					
time(min	0	30	60	90	120
1	0	93.49559228	217.1515635	340.6677	320.601
2	0	55.70888184	153.1120145	304.7542	311.787
3	0	50.07421017	126.7408908	236.3381	340.473
	0	66.4262281	165.6681563	293.92	324.287
	0	23.61144297	46.49478421	53.0019	14.6937
slope	2.920226123				
P_{app}	4.34557E-05				

PTX+HDS-3 20ug/mL					
BL-AP					
time(min	0	30	60	90	120
1	0	57.4294	113.884	300.8787889	493.589
2	0	87.4308	85.9153	193.2716144	503.495
3	0	43.9019	134.984	259.5327725	448.372
	0	62.9207	111.595	251.2277252	481.819
	0	22.278	24.6145	54.28219192	29.3858
slope	3.83981511				
P_{app}	5.714E-05				

Std

0.1	0.077	0.1	0.071
0.2	0.076	0.2	0.087
0.5	0.131	0.5	0.111
5	0.424	5	0.288
20	0.724	20	0.5
100	2.582	100	2.053
400	10.294	400	
500	11.034	500	10.359

A	B	A	B
43.0128	-8.028673282	48.76609677	-4.43294
