# Supplementary material for: Paclitaxel-Containing Extract Exerts Anti-Cancer Activity through Oral Administration in A549-Xenografted BALB/C Nude Mice: Synergistic Effect between Paclitaxel and Flavonoids or Lignoids
Source: Evid Based Complement Alternat Med. 2022 Apr 25;2022:3648175. doi: 10.1155/2022/3648175 (PMC9060980; doi:10.1155/2022/3648175)
Supplement: Supplementary Materials — Data are available in the supplement file. [file 3648175.f1.zip › 3648175.f1/Figure 7 MTT in A549 (1).pdf]

	control	HDS-2 0.78125 μg/mL	HDS-2 1.5625 μg/mL	HDS-2 3.125 μg/mL	HDS-2 6.25 μg/mL	HDS-2 12.5 μg/mL	HDS-2 25 μg/mL	HDS-2 50 μg/mL	HDS-2 100 μg/mL
A490nm value	0.876	0.818	0.858	0.859	0.748	0.801	0.701	0.709	0.652
	0.83	0.859	0.817	0.841	0.688	0.803	0.751	0.691	0.665
	0.806	0.822	0.804	0.851	0.802	0.712	0.717	0.703	0.595
	0.838	0.823	0.744	0.801	0.81	0.798	0.711	0.747	0.592
	0.817	0.885	0.812	0.701	0.834	0.699	0.708	0.658	0.668
	0.837	0.724	0.833	0.758	0.868	0.702	0.702	0.672	0.706
Mean	0.834								
viability %	105.04	98.08	102.88	103.00	89.69	96.04	84.05	85.01	78.18
	99.52	103.00	97.96	100.84	82.49	96.28	90.05	82.85	79.74
	96.64	98.56	96.40	102.04	96.16	85.37	85.97	84.29	71.34
	100.48	98.68	89.21	96.04	97.12	95.68	85.25	89.57	70.98
	97.96	106.12	97.36	84.05	100.00	83.81	84.89	78.90	80.10
	100.36	86.81	99.88	90.89	104.08	84.17	84.17	80.58	84.65
Mean	100	98.54	97.28	96.14	94.92	90.23	85.73	83.53	77.50
SD	2.88	6.56	4.57	7.45	7.72	6.35	2.23	3.74	5.36

	control	PTX 50nM	PTX 50nM+HDS-2 100 μg/mL	PTX 50nM+HDS-2 50 μg/mL	PTX 50nM+HDS-2 25 μg/mL	PTX 50nM+HDS-2 12.5 μg/mL	nM+HDS-2 6.2	PTX 50nM+HDS-2 3.125 μg/mL	PTX 50nM+HDS-2 1.5625 μg/mL
	0.787	0.503	0.39	0.373	0.403	0.403	0.457	0.485	0.483
	0.767	0.548	0.381	0.445	0.423	0.397	0.398	0.489	0.494
A490nm value	0.798	0.492	0.352	0.355	0.402	0.342	0.382	0.439	0.526
	0.898	0.562	0.371	0.387	0.438	0.401	0.381	0.431	0.483
	0.655	0.528	0.398	0.369	0.399	0.499	0.497	0.478	0.487
	0.688	0.631	0.344	0.398	0.369	0.372	0.365	0.387	0.358
Mean	0.7655								
	102.81	65.71	50.95	48.73	52.65	52.65	59.70	63.36	63.10
	100.20	71.59	49.77	58.13	55.26	51.86	51.99	63.88	64.53
viability %	104.25	64.27	45.98	46.37	52.51	44.68	49.90	57.35	68.71
	117.31	73.42	48.47	50.56	57.22	52.38	49.77	56.30	63.10
	85.56	68.97	51.99	48.20	52.12	65.19	64.92	62.44	63.62
	89.88	82.43	44.94	51.99	48.20	48.60	47.68	50.56	46.77
Mean	100.00	71.06	48.68	50.66	52.99	52.56	54.00	58.98	61.64
SD	11.28	6.54	2.78	4.14	3.07	6.90	6.79	5.21	7.59

	control	HDS-3 0.78125 g/mL	HDS-3 1.5625 g/mL	HDS-3 3.125 g/mL	HDS-3 6.25 g/mL	HDS-3 12.5 g/mL	HDS-3 25 g/mL	HDS-3 50 g/mL	HDS-3 100 g/mL
A490nm value	0.573	0.713	0.734	0.725	0.709	0.695	0.593	0.632	0.664
	0.713	0.733	0.788	0.613	0.741	0.709	0.698	0.686	0.628
	0.812	0.665	0.675	0.733	0.621	0.671	0.773	0.647	0.645
	0.819	0.605	0.637	0.702	0.606	0.551	0.529	0.683	0.634
	0.741	0.732	0.736	0.634	0.723	0.748	0.754	0.679	0.549
	0.694	0.841	0.836	0.779	0.794	0.719	0.732	0.559	0.437
Mean	0.725333								
viability %	79.00	98.30	101.19	99.95	97.75	95.82	81.76	87.13	91.54
	98.30	101.06	108.64	84.51	102.16	97.75	96.23	94.58	86.58
	111.95	91.68	93.06	101.06	85.62	92.51	106.57	89.20	88.92
	112.91	83.41	87.82	96.78	83.55	75.97	72.93	94.16	87.41
	102.16	100.92	101.47	87.41	99.68	103.13	103.95	93.61	75.69
	95.68	115.95	115.26	107.40	109.47	99.13	100.92	77.07	60.25
Mean	100.00	98.55	101.24	96.19	96.37	94.05	93.73	89.29	81.73
SD	12.46	10.87	9.98	8.69	9.98	9.53	13.44	6.70	11.85

	control	PTX 50nM	PTX 50nM+HDS-3 100 g/mL	PTX 50nM+HDS-3 50 g/mL	PTX 50nM+HDS-3 25 g/mL	PTX 50nM+HDS-3 12.5 g/mL	50nM+HDS-3 6.25	PTX 50nM+HDS-3 3.125 g/mL	PTX 50nM+HDS-3 1.5625 g/mL
A490nm value	0.819	0.561	0.438	0.475	0.469	0.532	0.469	0.532	0.524
	0.814	0.616	0.396	0.429	0.456	0.427	0.535	0.661	0.523
	0.802	0.571	0.441	0.449	0.449	0.493	0.555	0.531	0.438
	0.825	0.559	0.463	0.55	0.581	0.582	0.548	0.542	0.542
	0.652	0.565	0.442	0.436	0.486	0.537	0.521	0.514	0.646
	0.812	0.561	0.401	0.437	0.494	0.552	0.494	0.458	0.51
Mean	0.787333								

viability %	104.02	71.25	55.63	60.33	59.57	67.57	59.57	67.57	66.55
	103.39	78.24	50.30	54.49	57.92	54.23	67.95	83.95	66.43
	101.86	72.52	56.01	57.03	57.03	62.62	70.49	67.44	55.63
	104.78	71.00	58.81	69.86	73.79	73.92	69.60	68.84	68.84
	82.81	71.76	56.14	55.38	61.73	68.20	66.17	65.28	82.05
	103.13	71.25	50.93	55.50	62.74	70.11	62.74	58.17	64.78
Mean	100.00	72.67	54.64	58.76	62.13	66.11	66.09	68.54	67.38
SD	8.48	2.78	3.32	5.81	6.11	6.88	4.22	8.46	8.53
