# Supplementary material for: Paclitaxel-Containing Extract Exerts Anti-Cancer Activity through Oral Administration in A549-Xenografted BALB/C Nude Mice: Synergistic Effect between Paclitaxel and Flavonoids or Lignoids
Source: Evid Based Complement Alternat Med. 2022 Apr 25;2022:3648175. doi: 10.1155/2022/3648175 (PMC9060980; doi:10.1155/2022/3648175)
Supplement: Supplementary Materials — Data are available in the supplement file. [file 3648175.f1.zip › 3648175.f1/Figure 9 data.pdf]

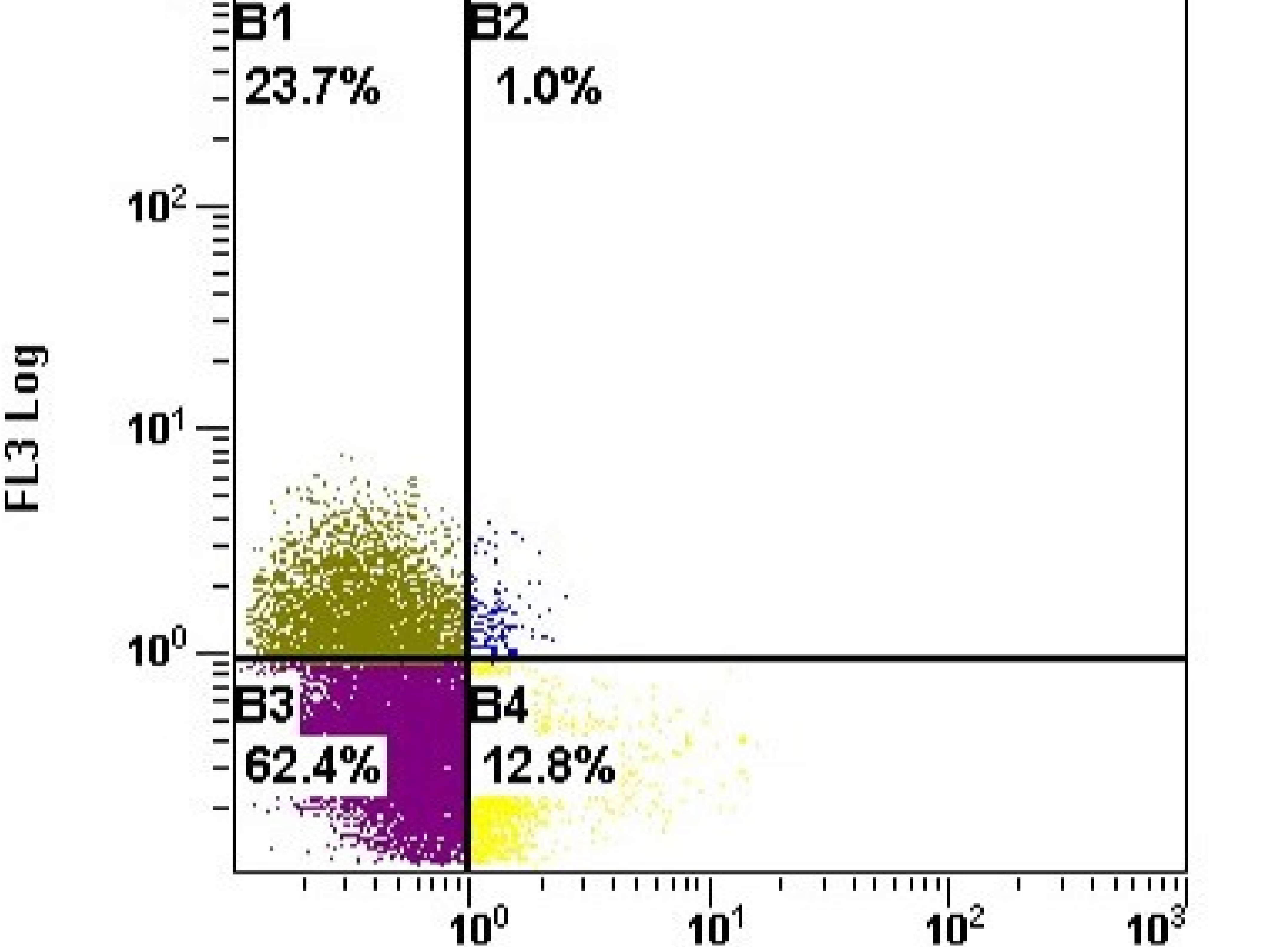
B1
23.7%
B2
1.0%
B3
62.4%
B4
12.8%
FL3 Log
10^2
10^1
10^0
10^0
10^1
10^2
10^3

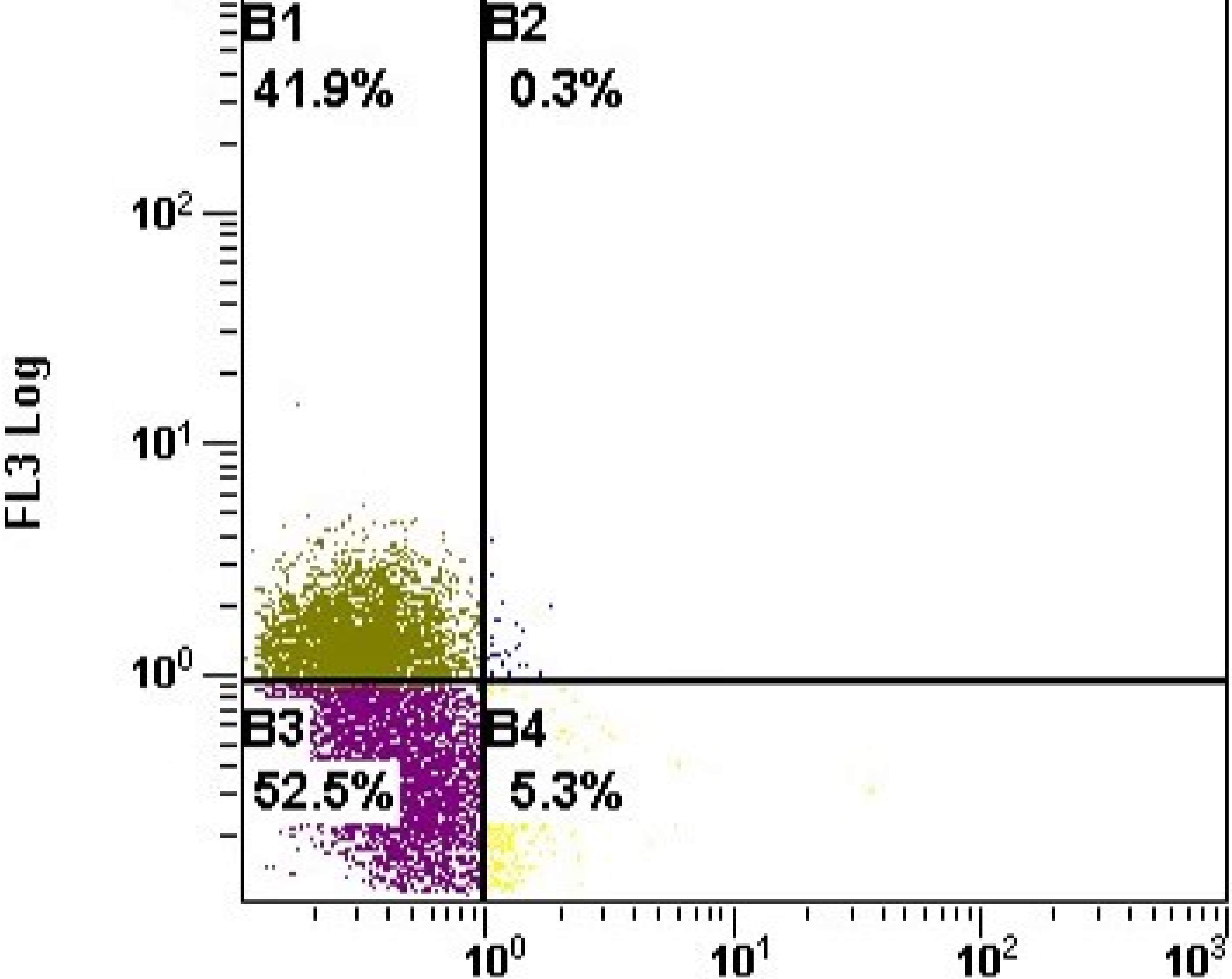
B1
41.9%
B2
0.3%
B3
52.5%
B4
5.3%
FL3 Log
10^2
10^1
10^0
10^0
10^1
10^2
10^3

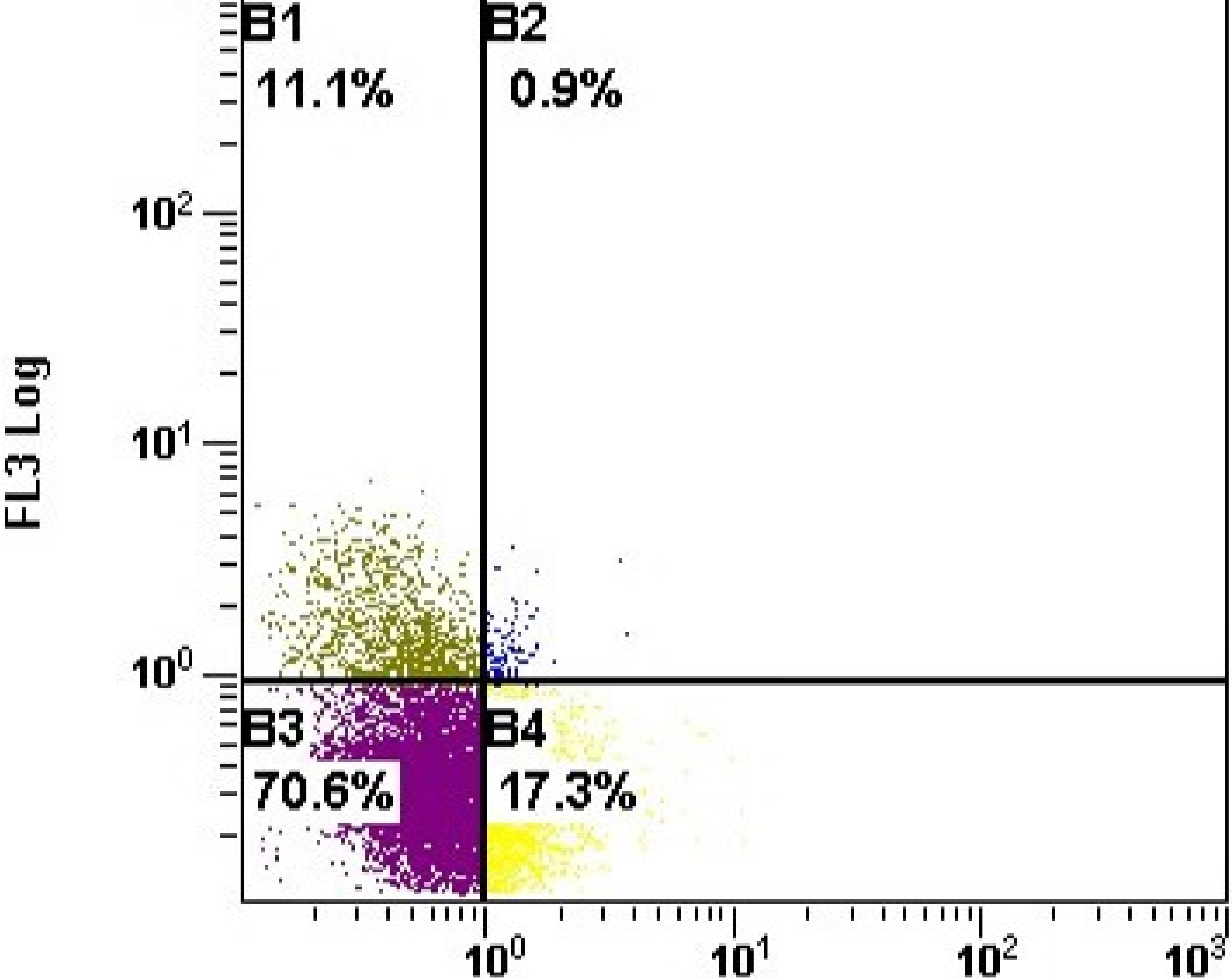

B1
11.1%
B2
0.9%
B3
70.6%
B4
17.3%
FL3 Log
10^0
10^1
10^2
10^3

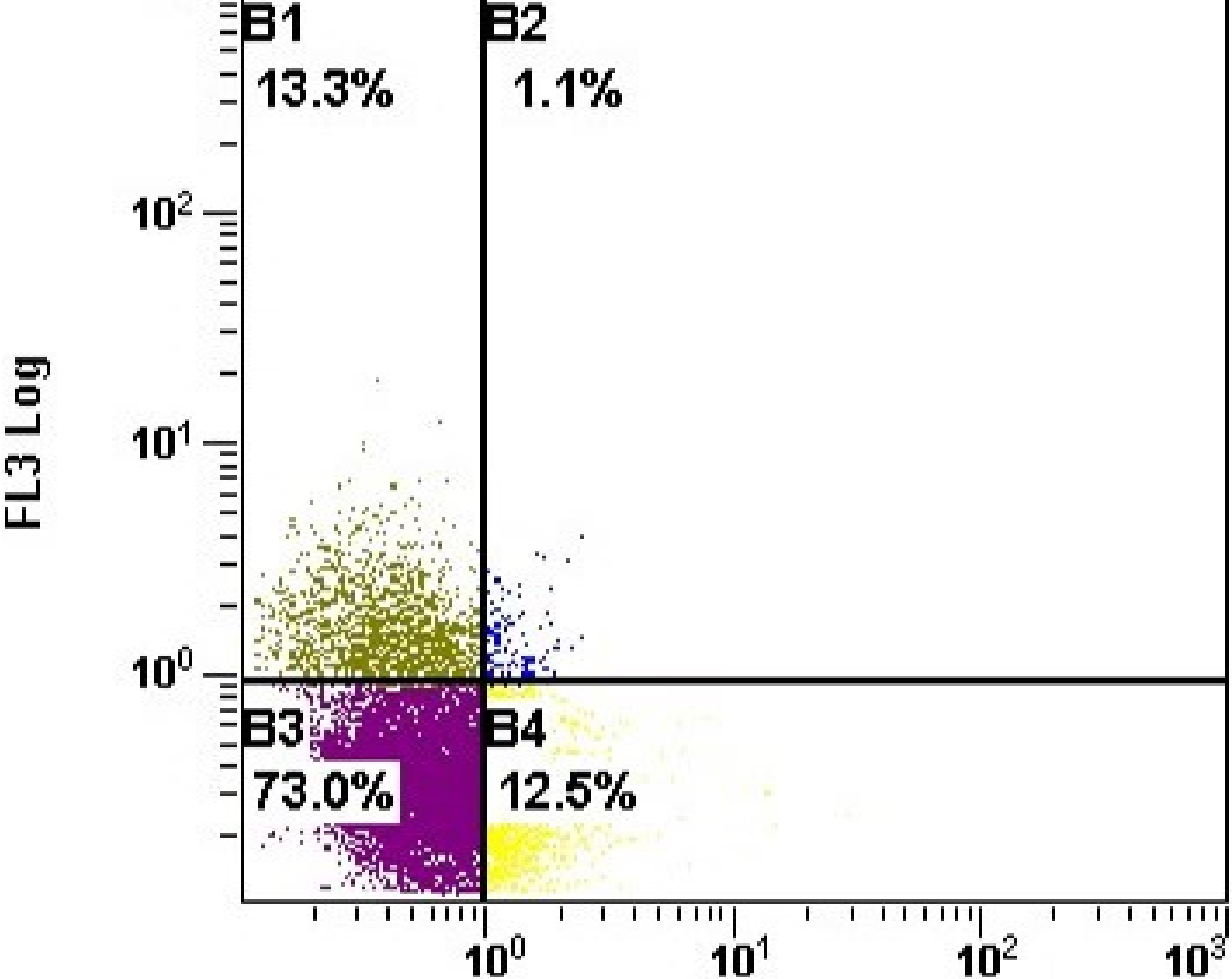

B1
13.3%
B2
1.1%
B3
73.0%
B4
12.5%
FL3 Log
10^2
10^1
10^0
10^0
10^1
10^2
10^3

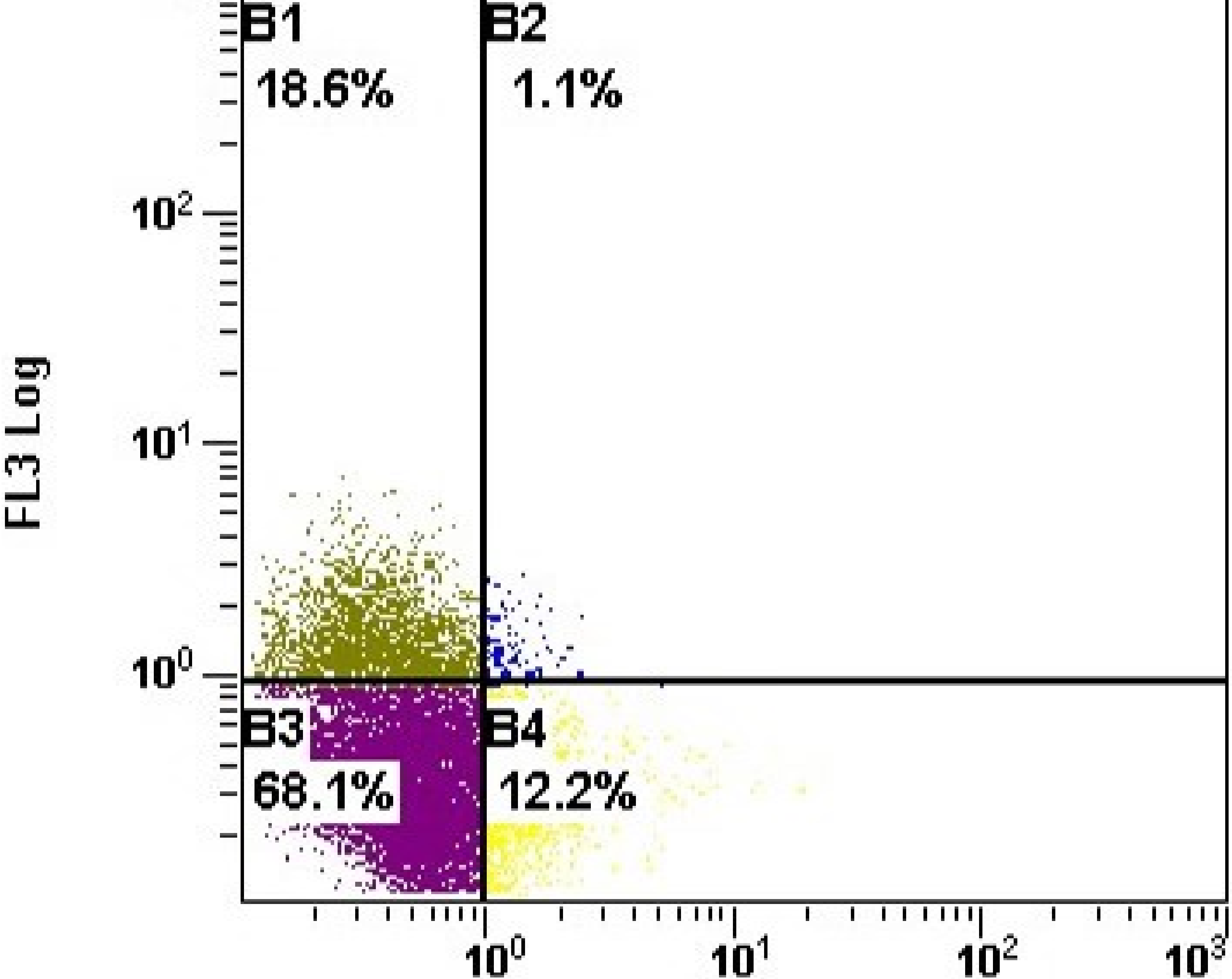

FL3 Log
B1
18.6%
B2
1.1%
B3
68.1%
B4
12.2%
10^2
10^1
10^0
10^0
10^1
10^2
10^3

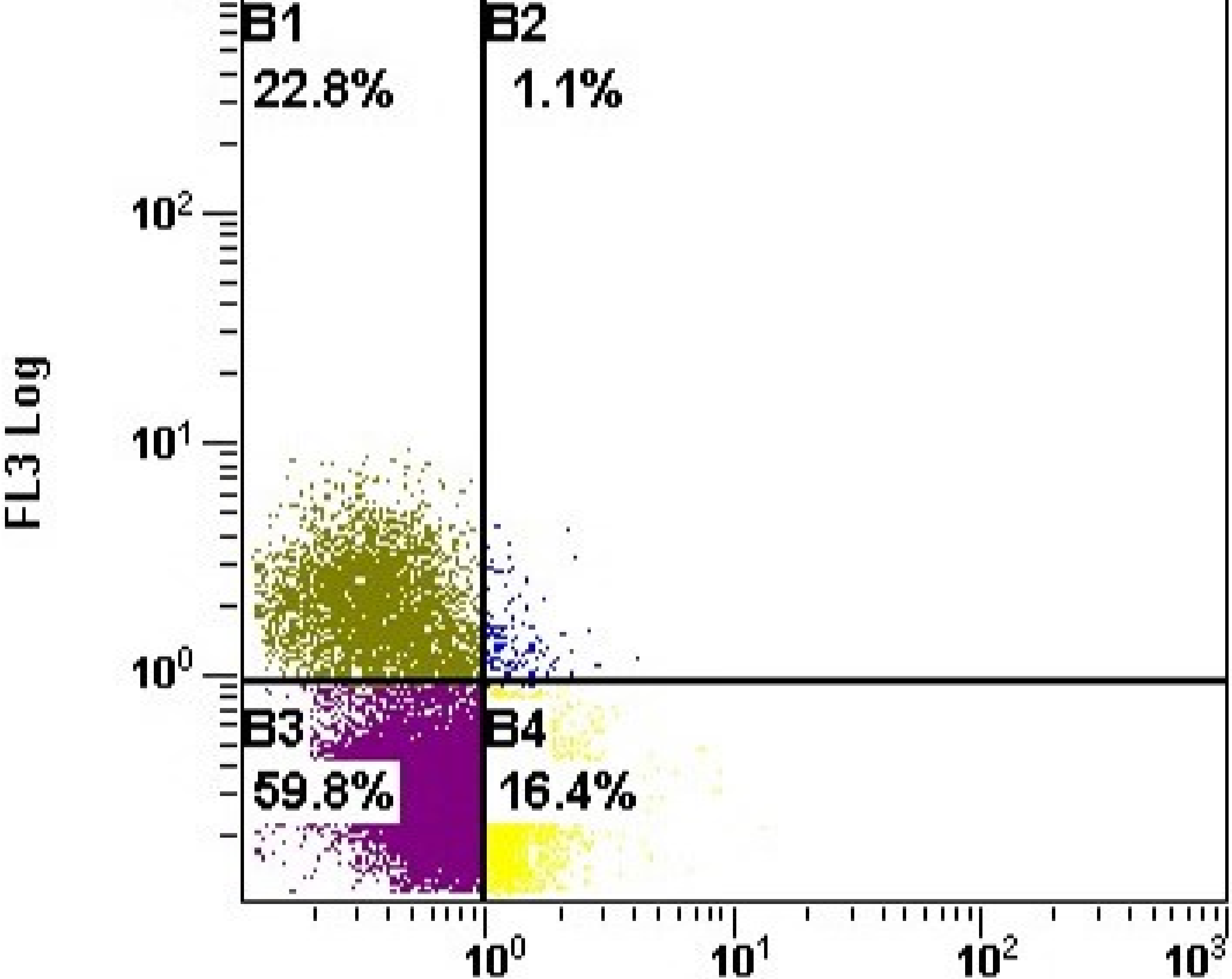
B1
22.8%
B2
1.1%
B3
59.8%
B4
16.4%
FL3 Log
10^2
10^1
10^0
10^0
10^1
10^2
10^3

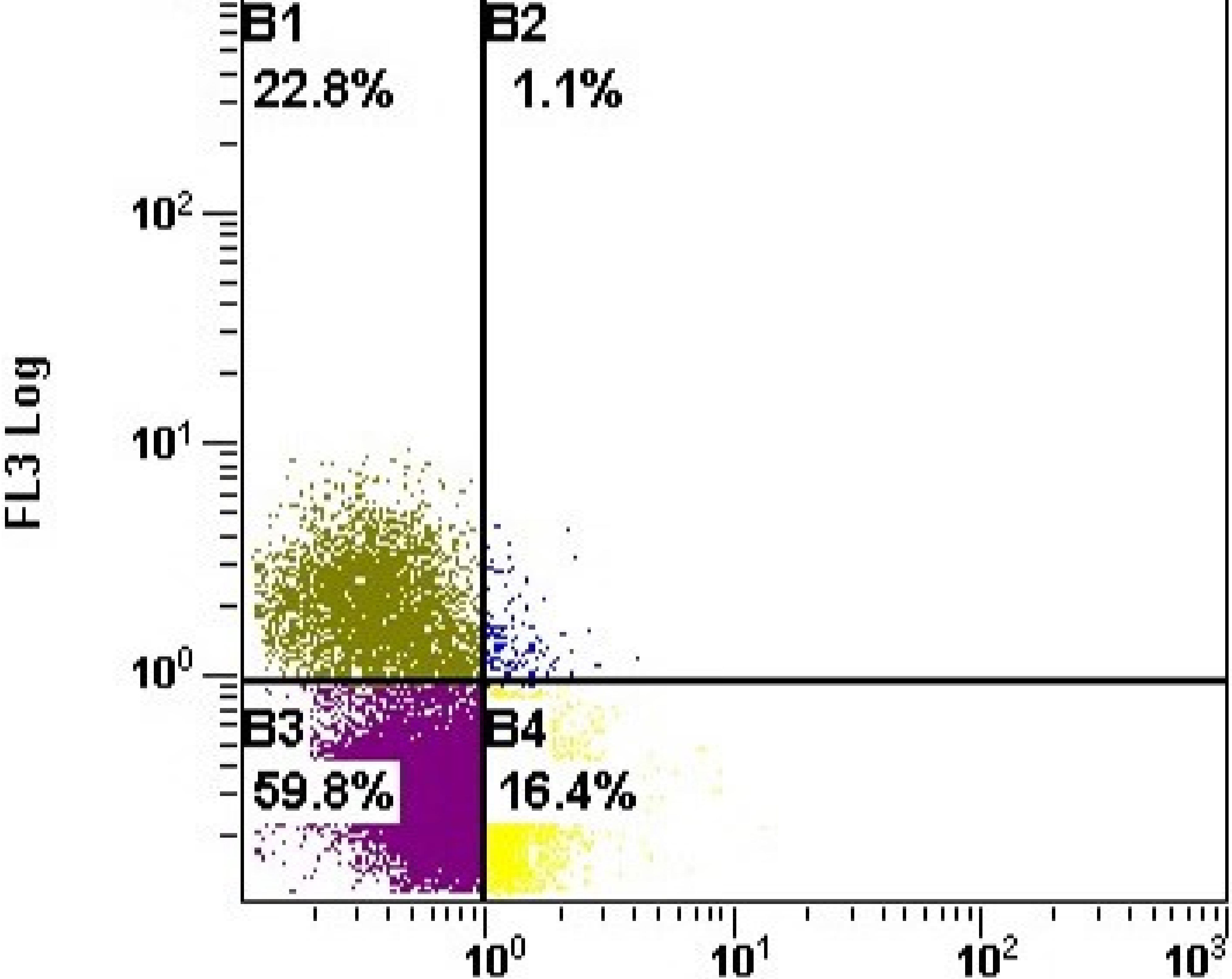

B1
22.8%
B2
1.1%
B3
59.8%
B4
16.4%
FL3 Log
10^2
10^1
10^0
10^0
10^1
10^2
10^3

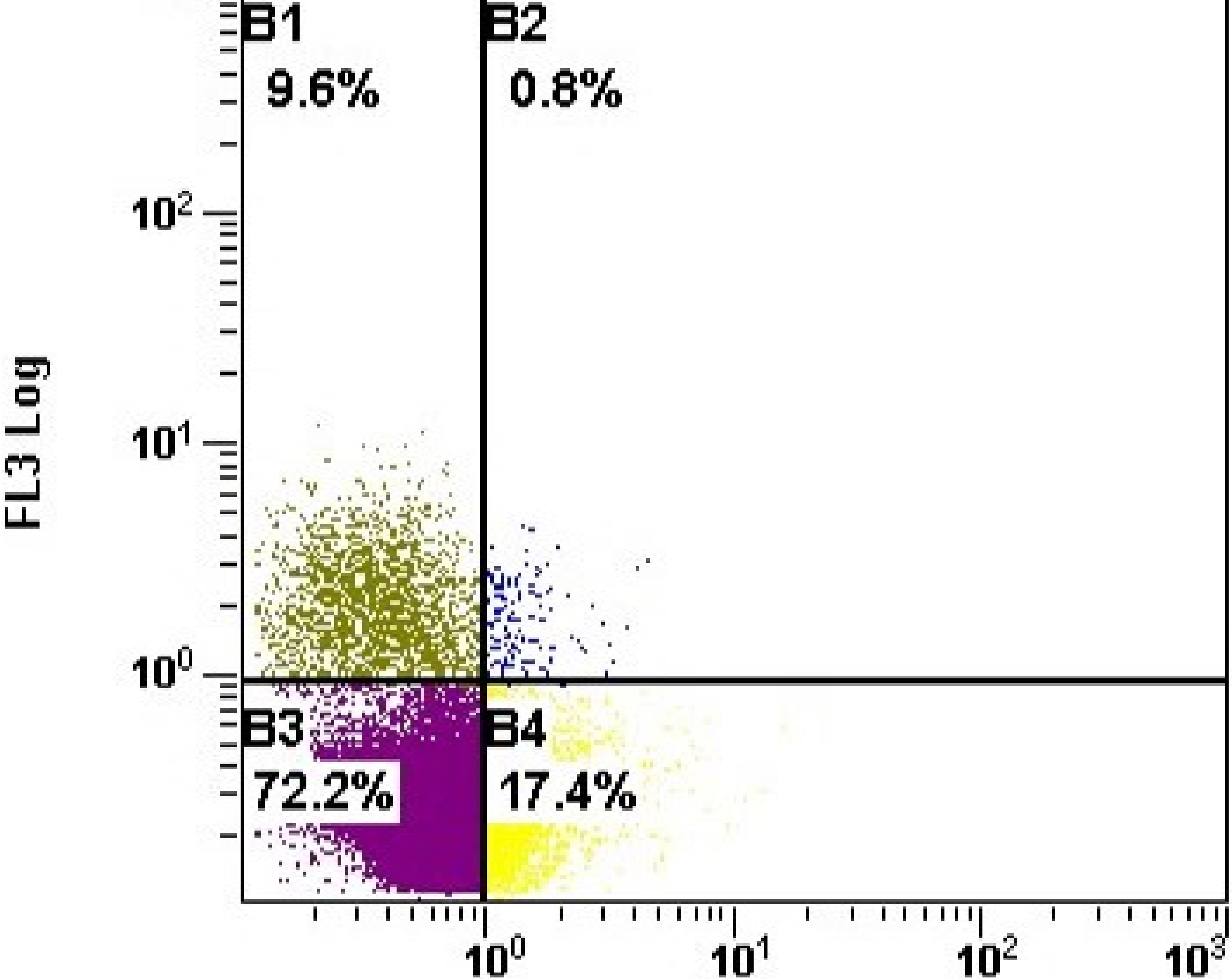

B1
9.6%
B2
0.8%
B3
72.2%
B4
17.4%
FL3 Log
10^2
10^1
10^0
10^0
10^1
10^2
10^3

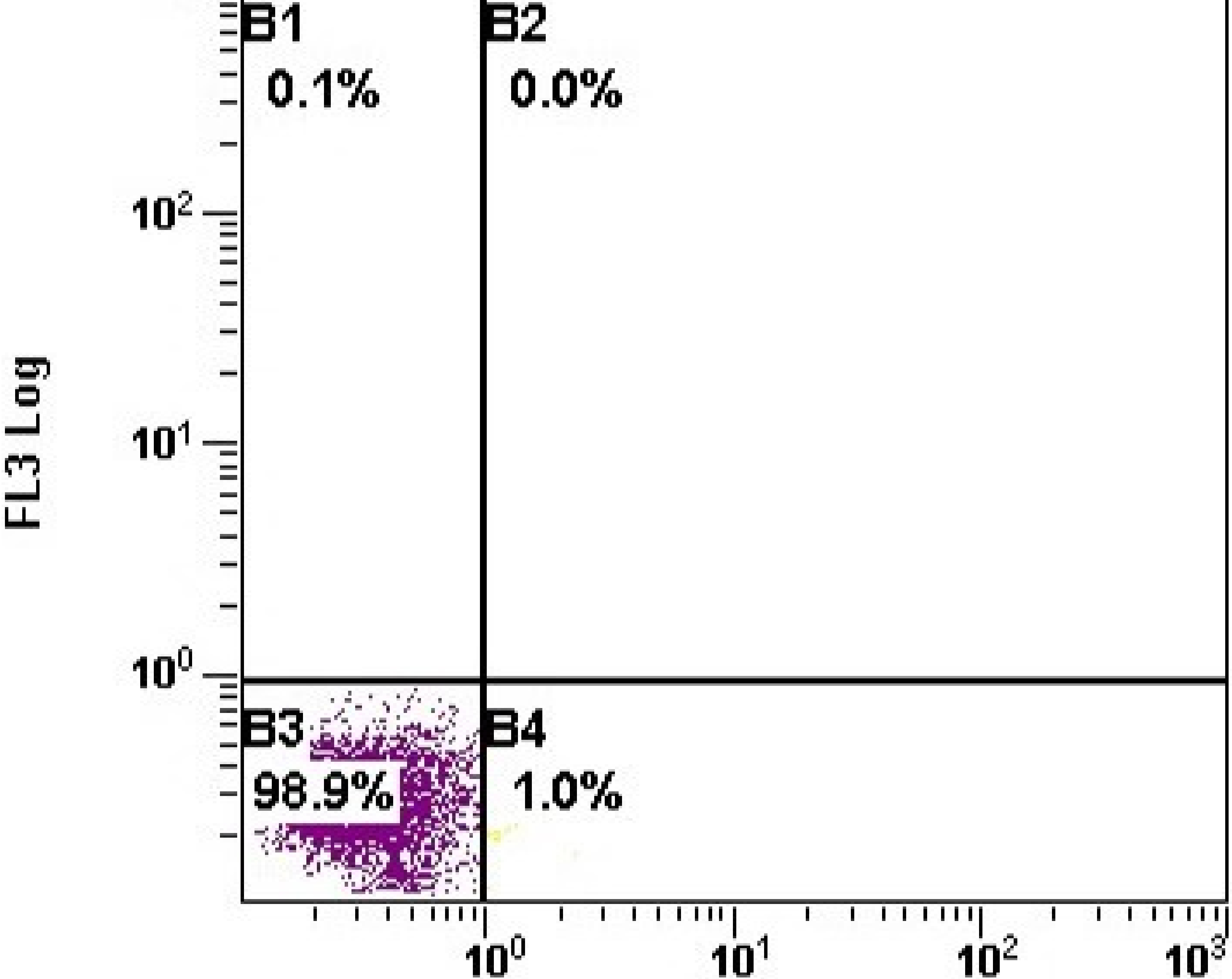
B1
0.1%
B2
0.0%
B3
98.9%
B4
1.0%
FL3 Log
10^2
10^1
10^0
10^0
10^1
10^2
10^3

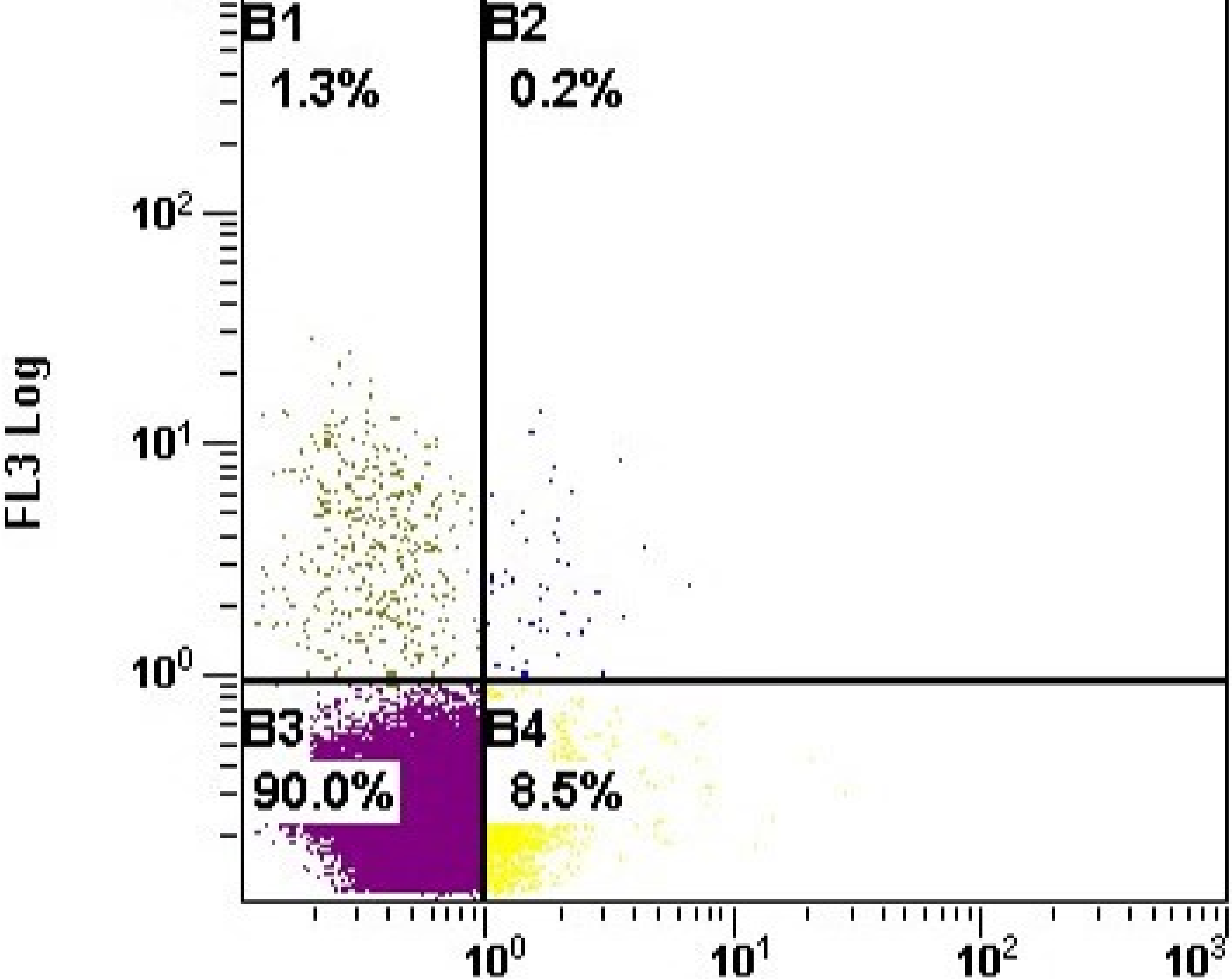
FL3 Log
B1
1.3%
B2
0.2%
B3
90.0%
B4
8.5%
10^2
10^1
10^0
10^0
10^1
10^2
10^3

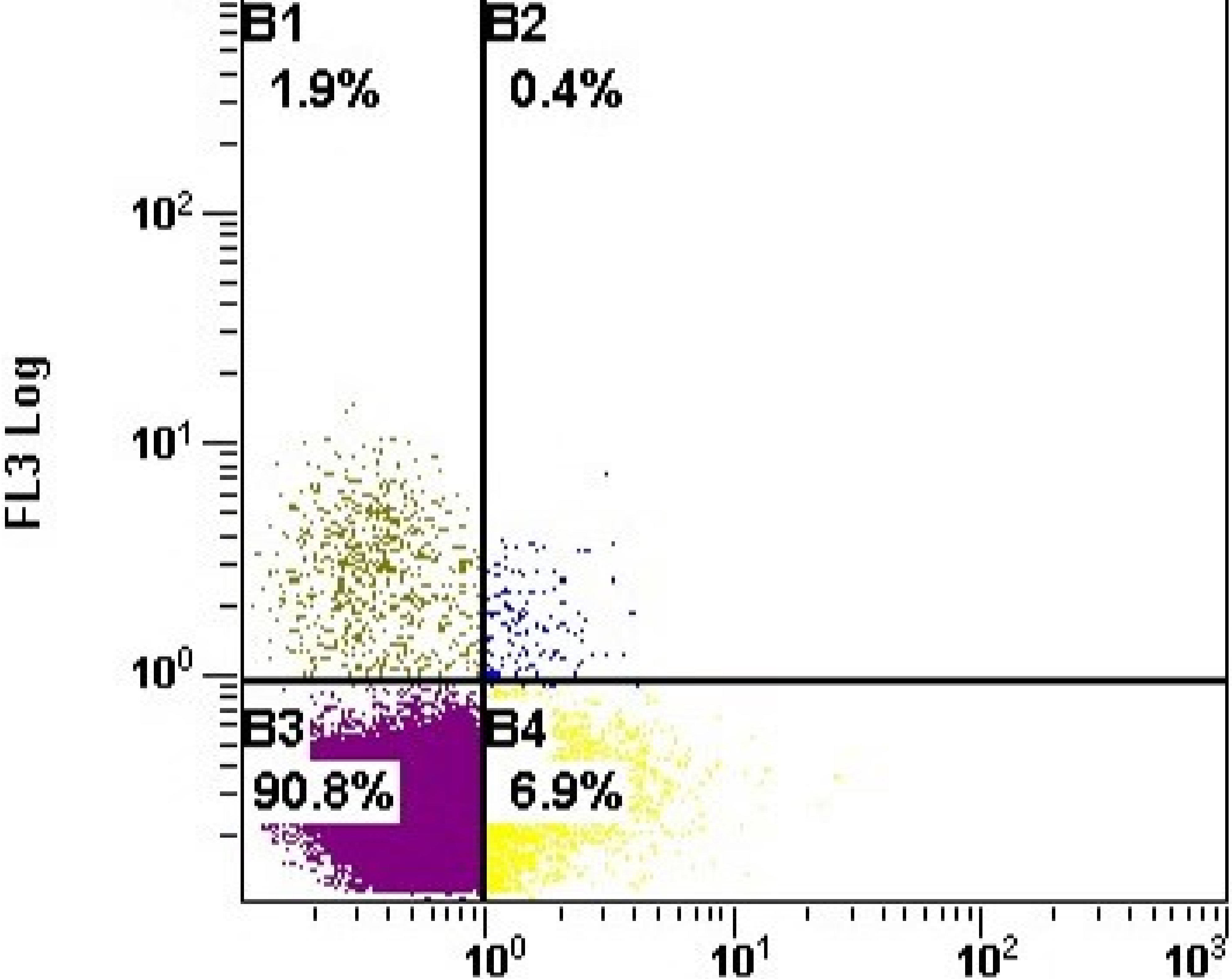

B1
1.9%
B2
0.4%
B3
90.8%
B4
6.9%
FL3 Log
10^2
10^1
10^0
10^0
10^1
10^2
10^3

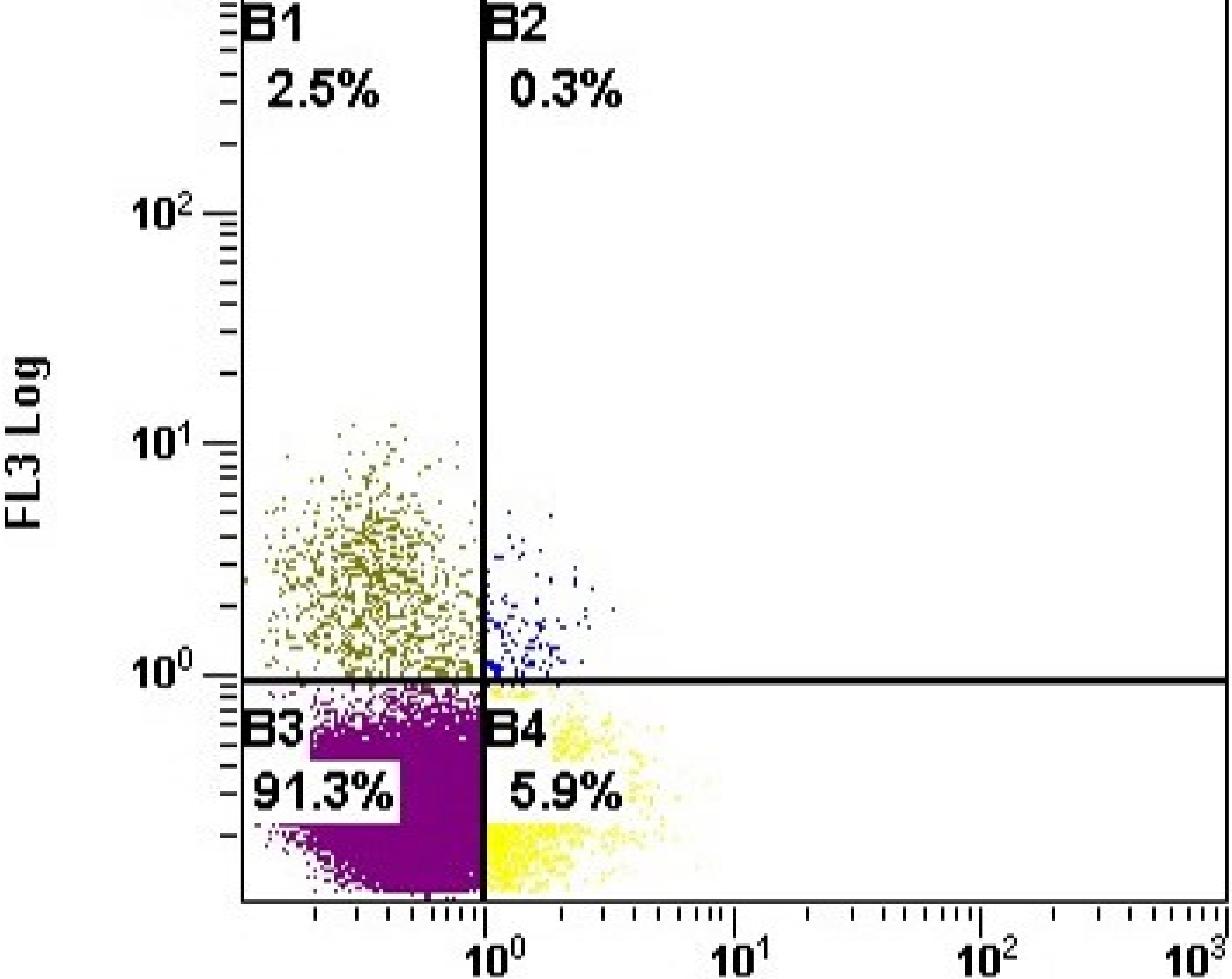

FL3 Log
B1
2.5%
B2
0.3%
B3
91.3%
B4
5.9%
10^2
10^1
10^0
10^0
10^1
10^2
10^3

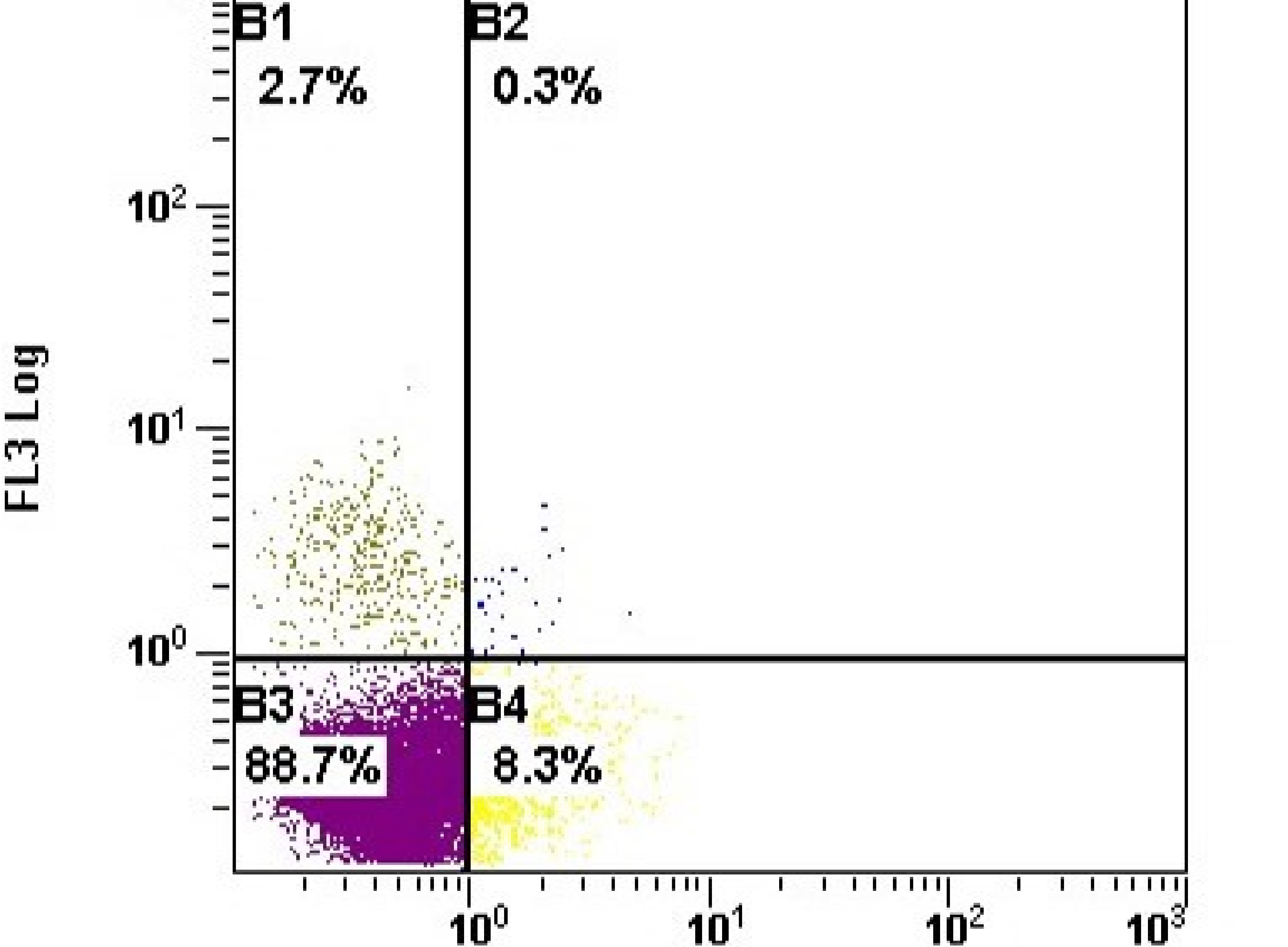
B1
2.7%
B2
0.3%
B3
88.7%
B4
8.3%
FL3 Log
10^2
10^1
10^0
10^0
10^1
10^2
10^3

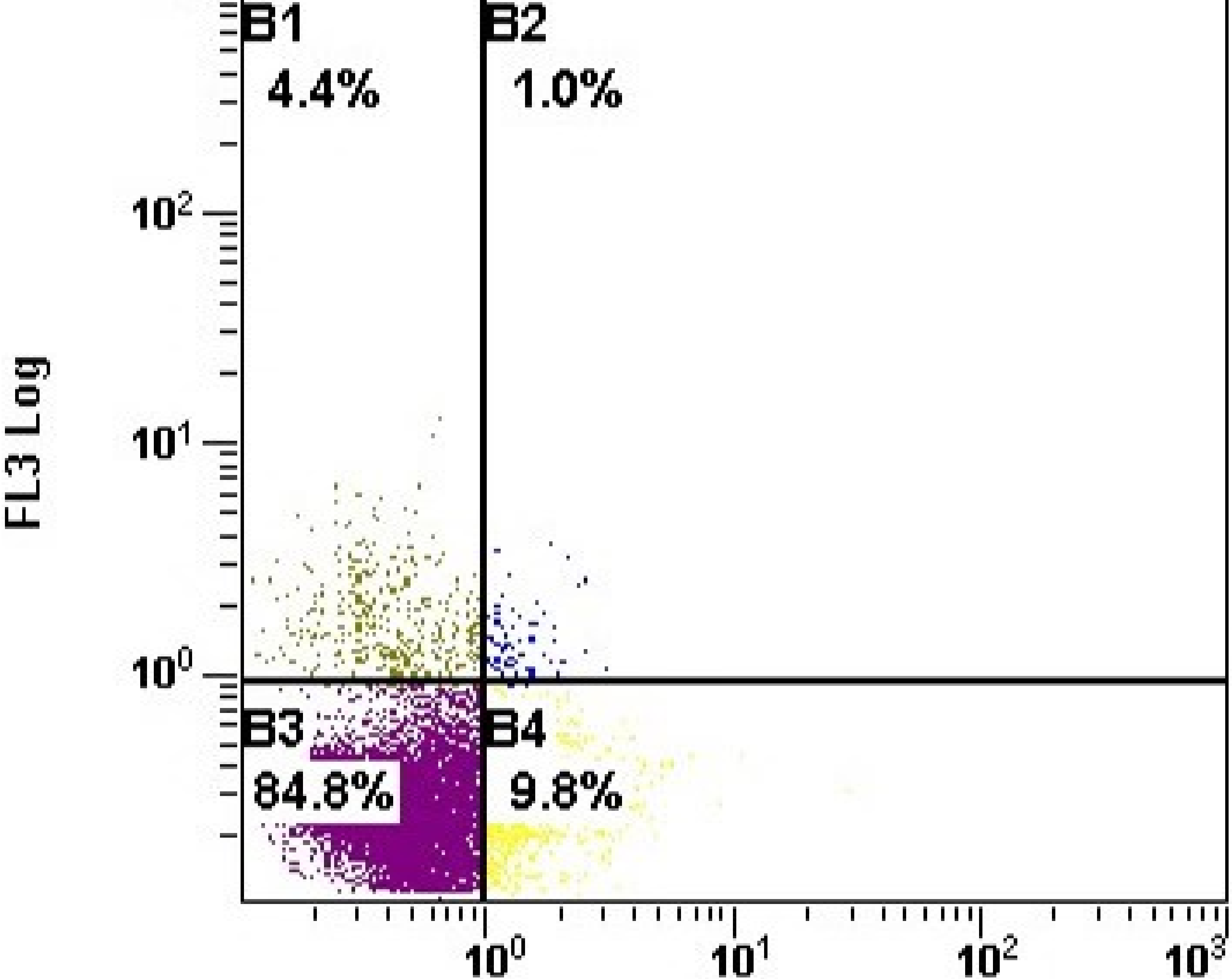

B1
4.4%
B2
1.0%
B3
84.8%
B4
9.8%
FL3 Log
10^0
10^1
10^2
10^3

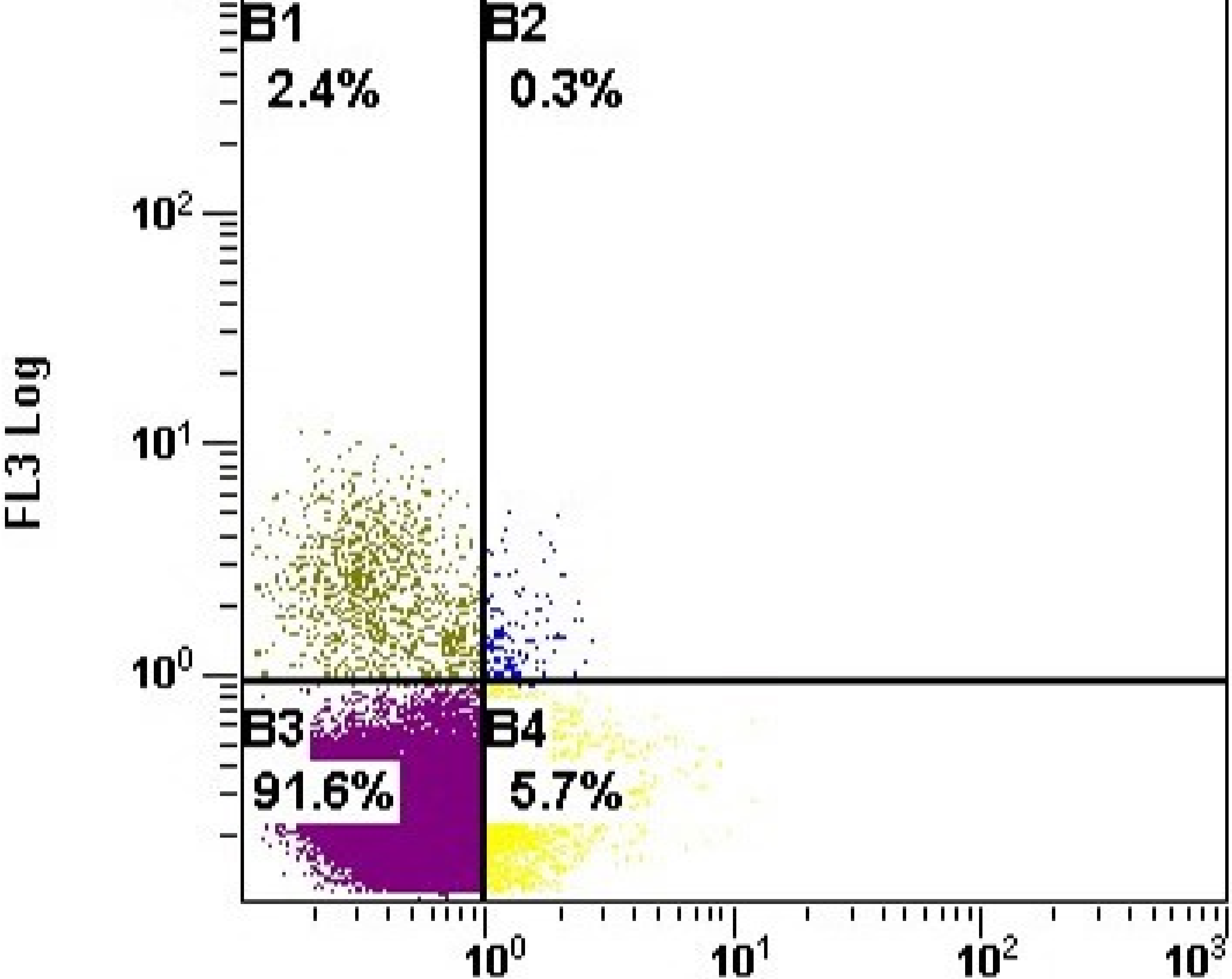
B1
2.4%
B2
0.3%
B3
91.6%
B4
5.7%
FL3 Log
10^2
10^1
10^0
10^0
10^1
10^2
10^3

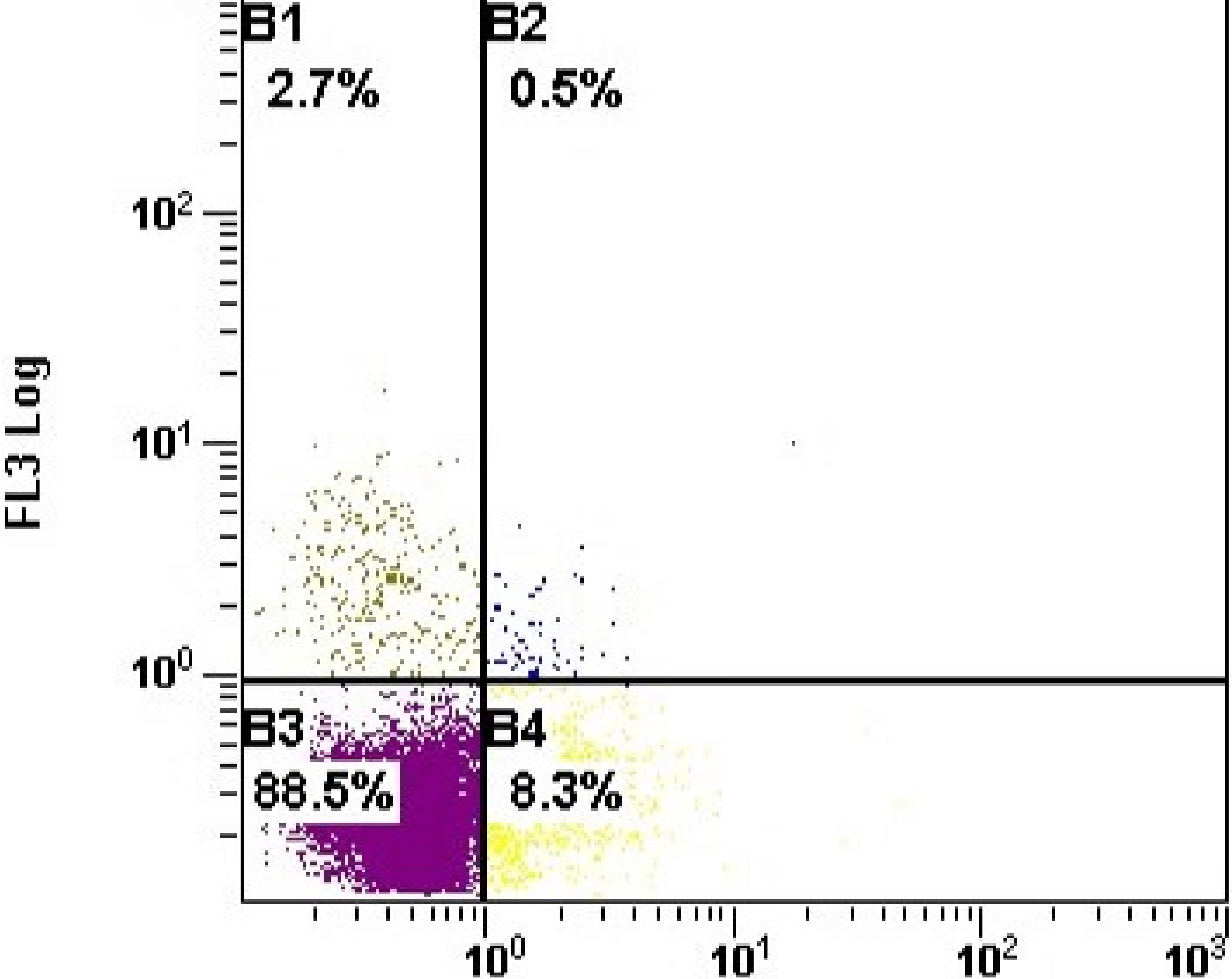

FL3 Log
B1
2.7%
B2
0.5%
B3
88.5%
B4
8.3%
10^2
10^1
10^0
10^0
10^1
10^2
10^3

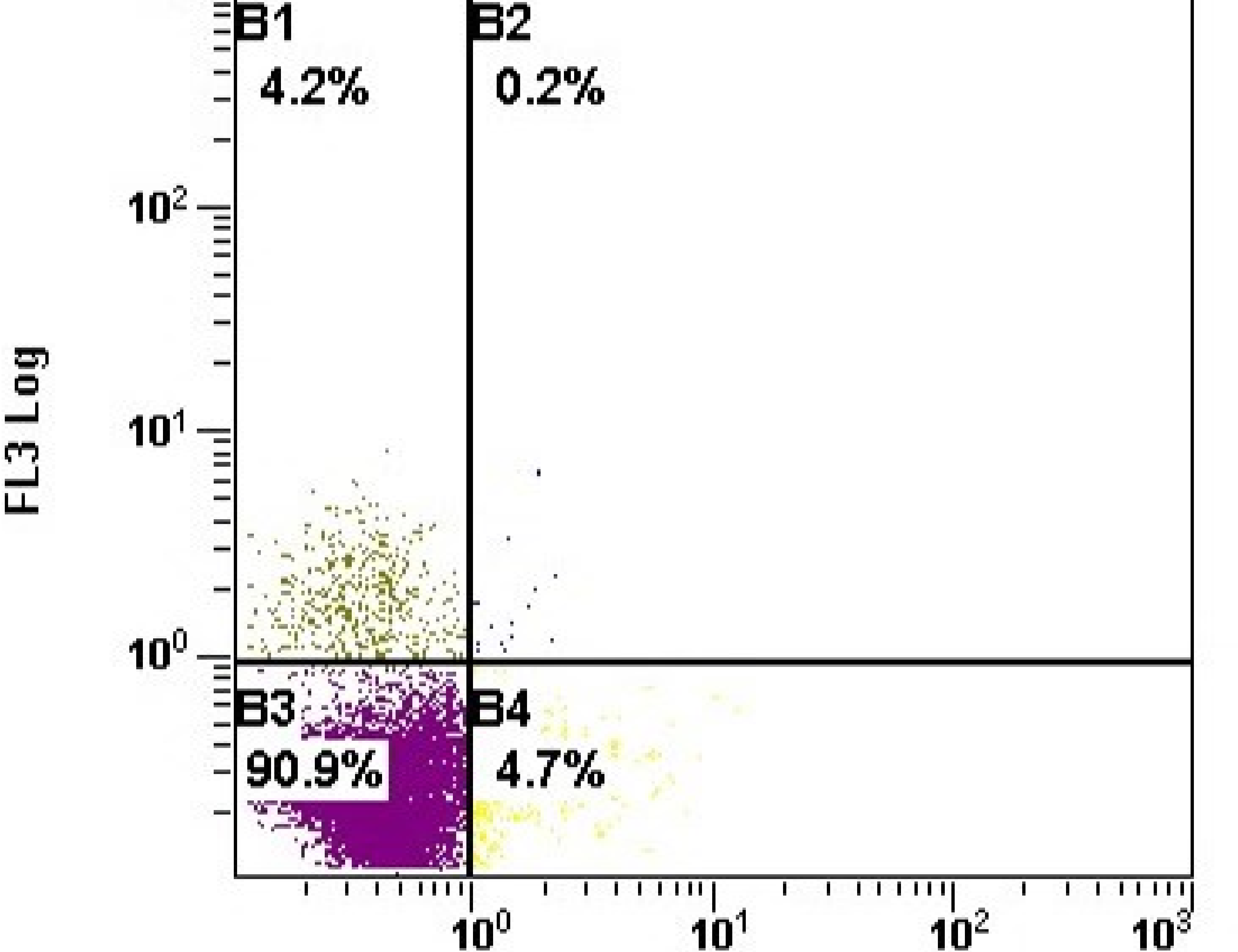

B1
4.2%
B2
0.2%
B3
90.9%
B4
4.7%
FL3 Log
10^0
10^1
10^2
10^3

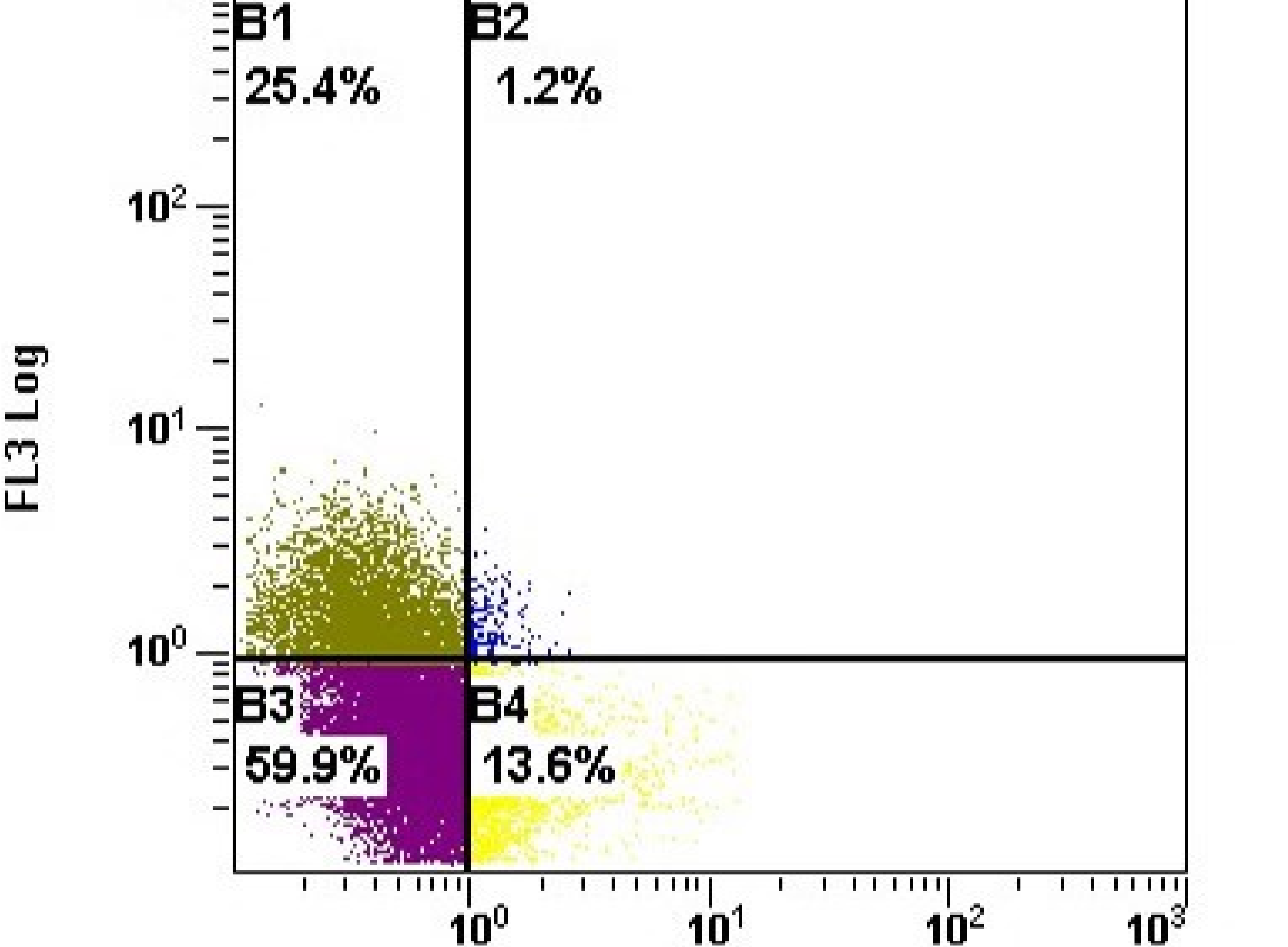
B1
25.4%
B2
1.2%
B3
59.9%
B4
13.6%
FL3 Log
10^0
10^1
10^2
10^0
10^1
10^2
10^3
