# Supplementary figures and images for: Paclitaxel-Containing Extract Exerts Anti-Cancer Activity through Oral Administration in A549-Xenografted BALB/C Nude Mice: Synergistic Effect between Paclitaxel and Flavonoids or Lignoids
Source: Evid Based Complement Alternat Med. 2022 Apr 25;2022:3648175. doi: 10.1155/2022/3648175 (PMC9060980; doi:10.1155/2022/3648175)

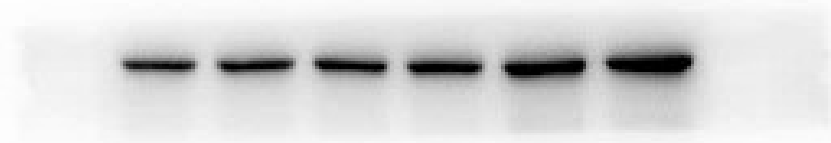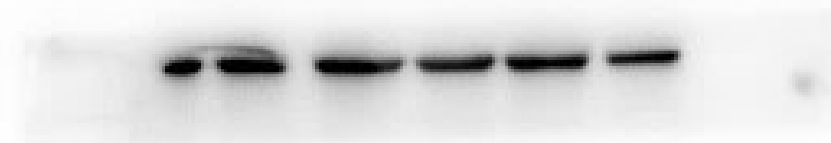

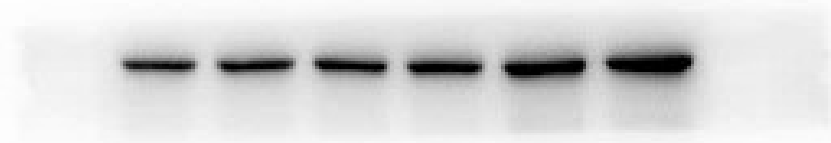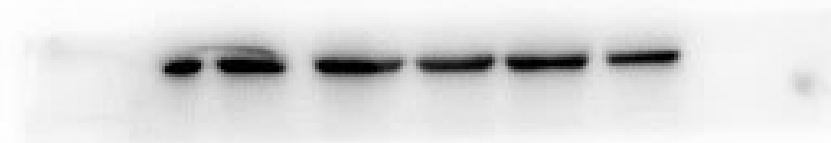

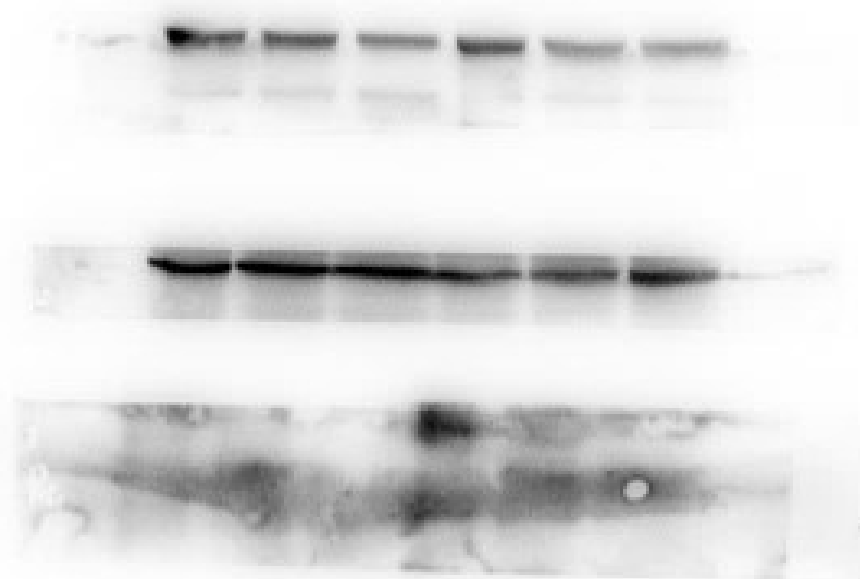

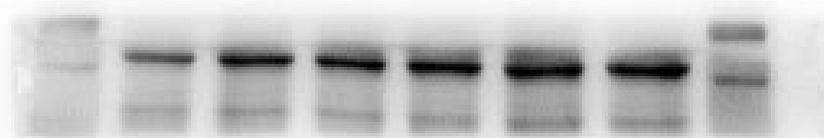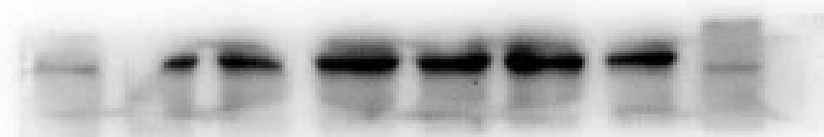

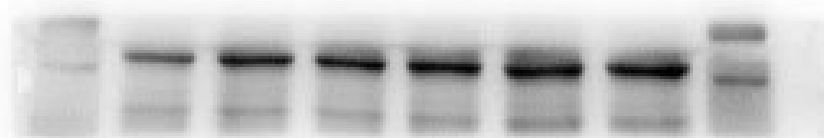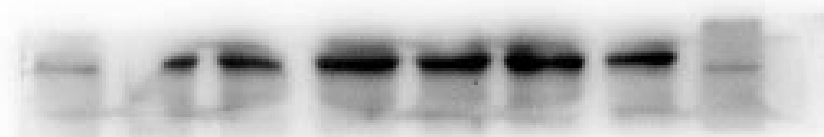

100 100 100 100 100 100

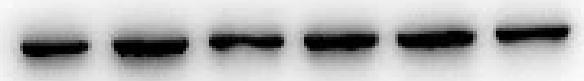

100 100 100 100 100 100

100 100 100 100 100 100

100 100 100 100 100 100

100 100 100 100 100 100

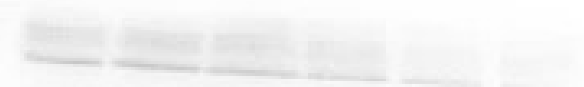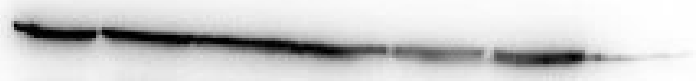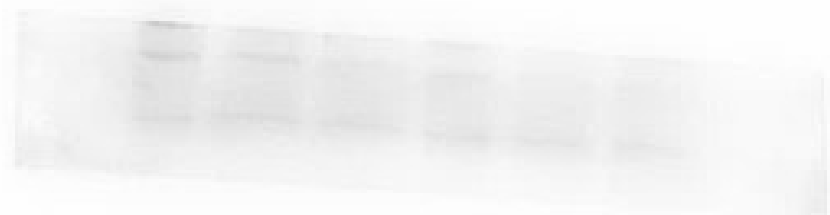

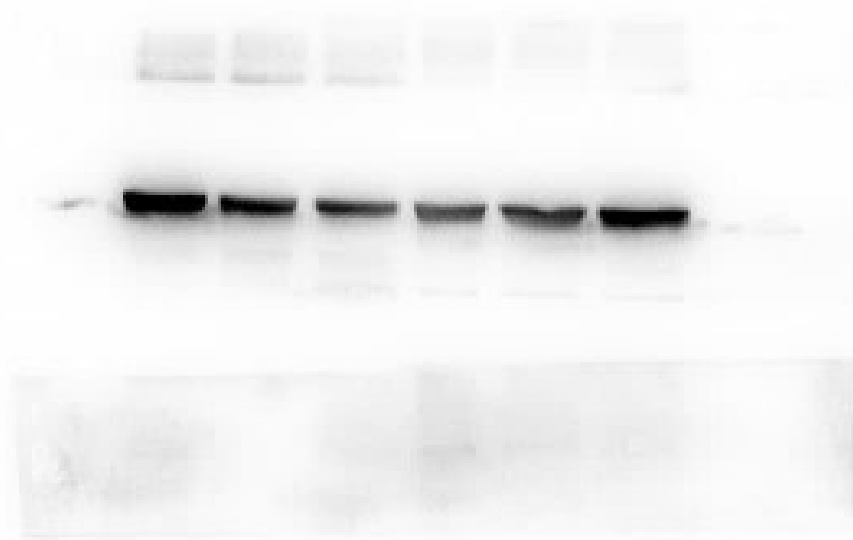

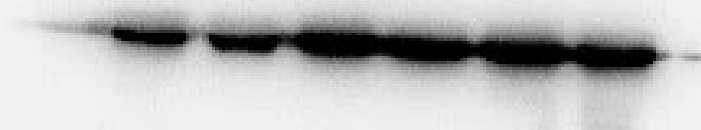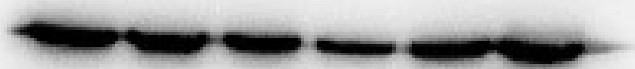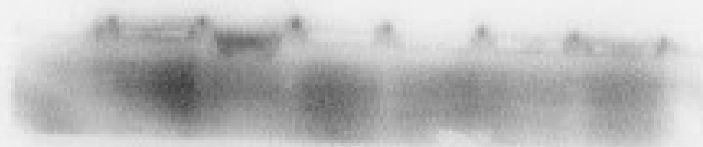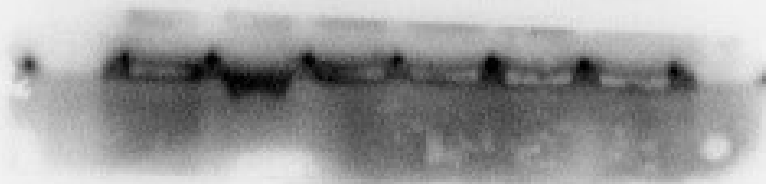

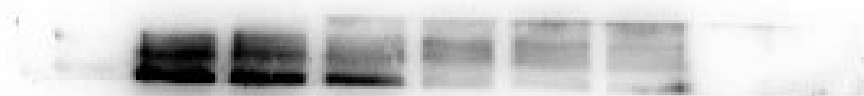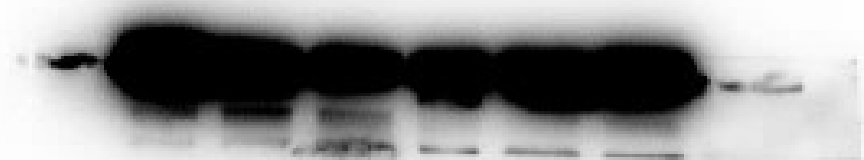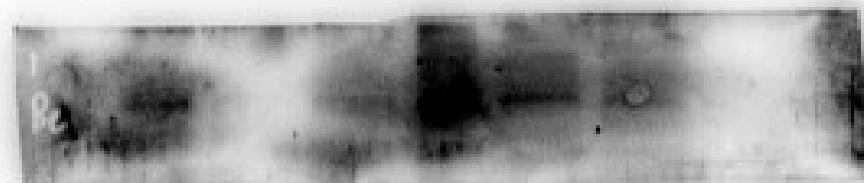

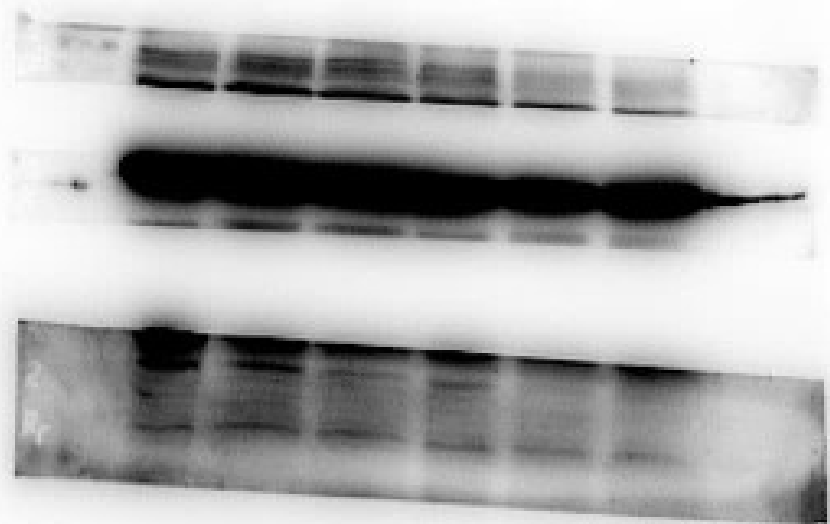

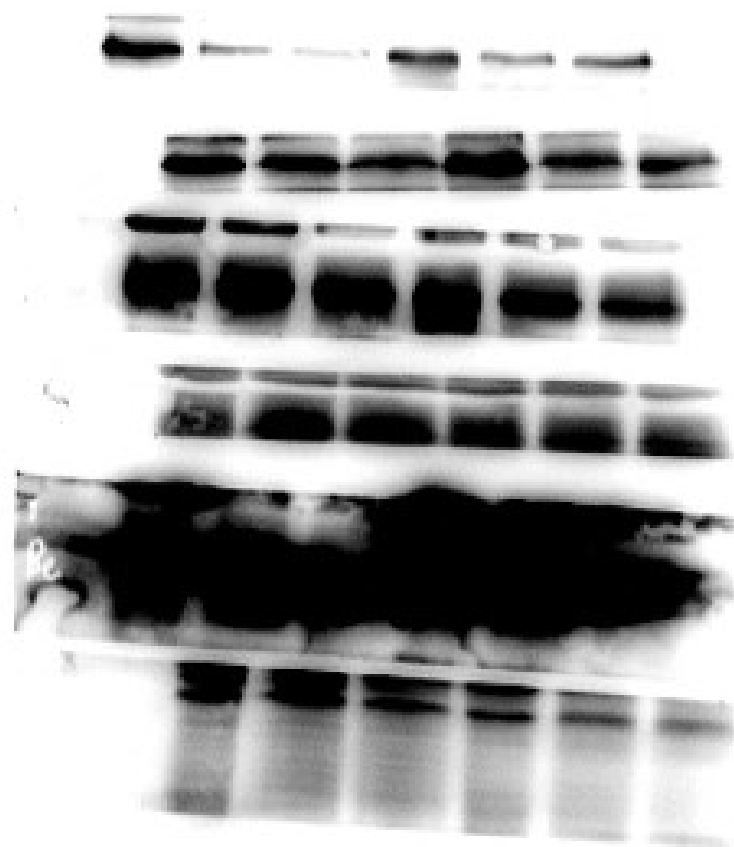

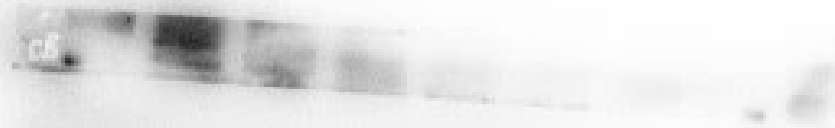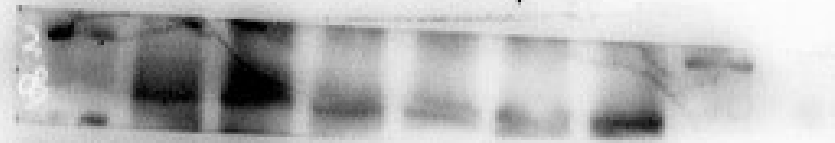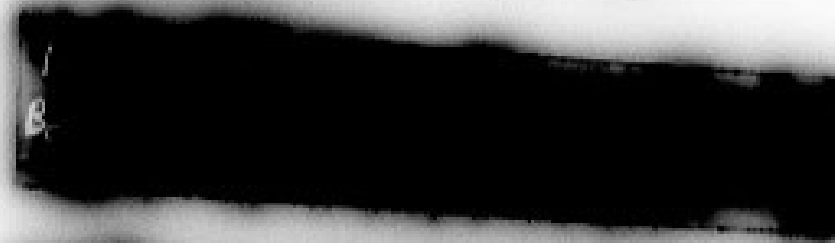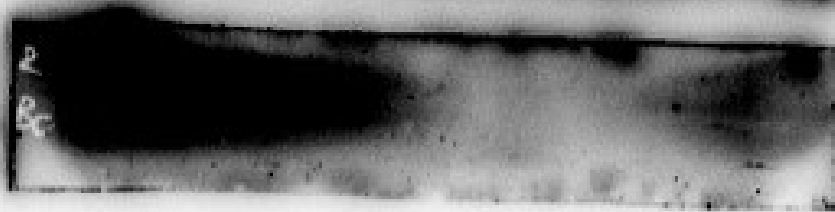

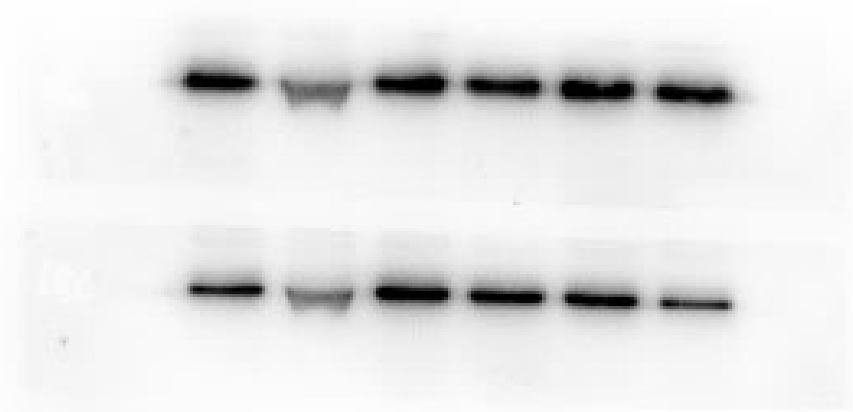

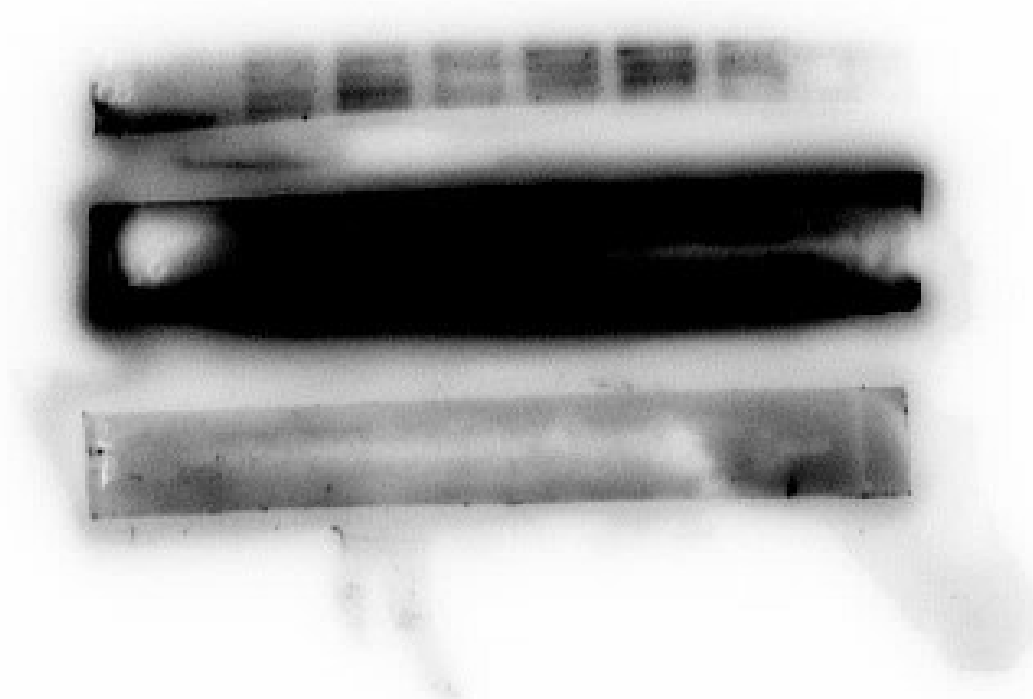

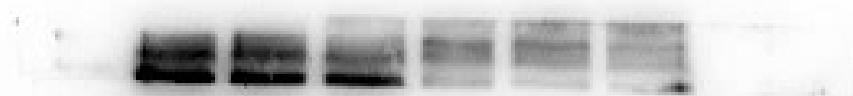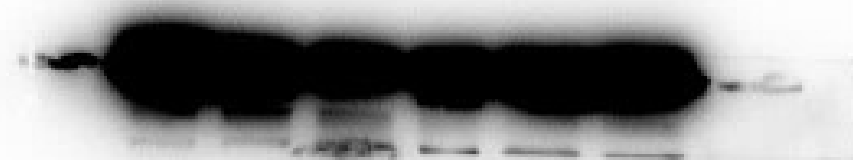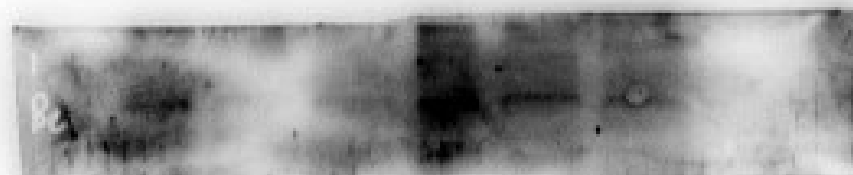

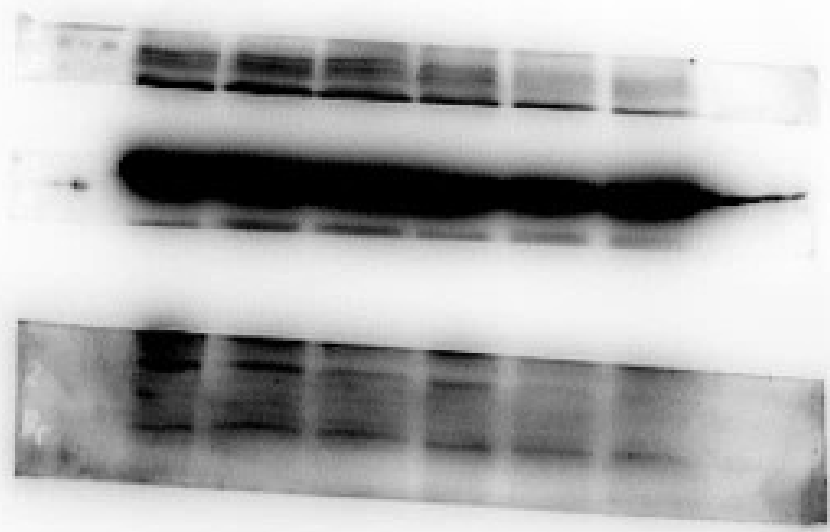

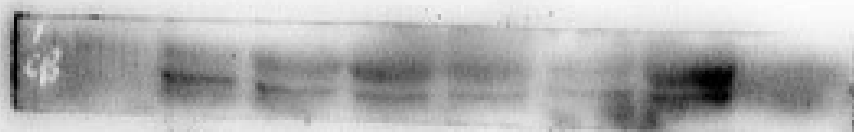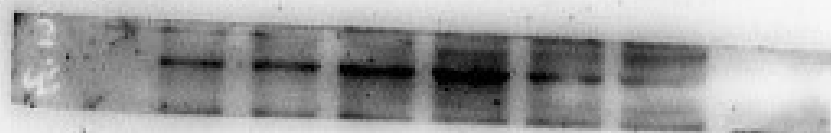

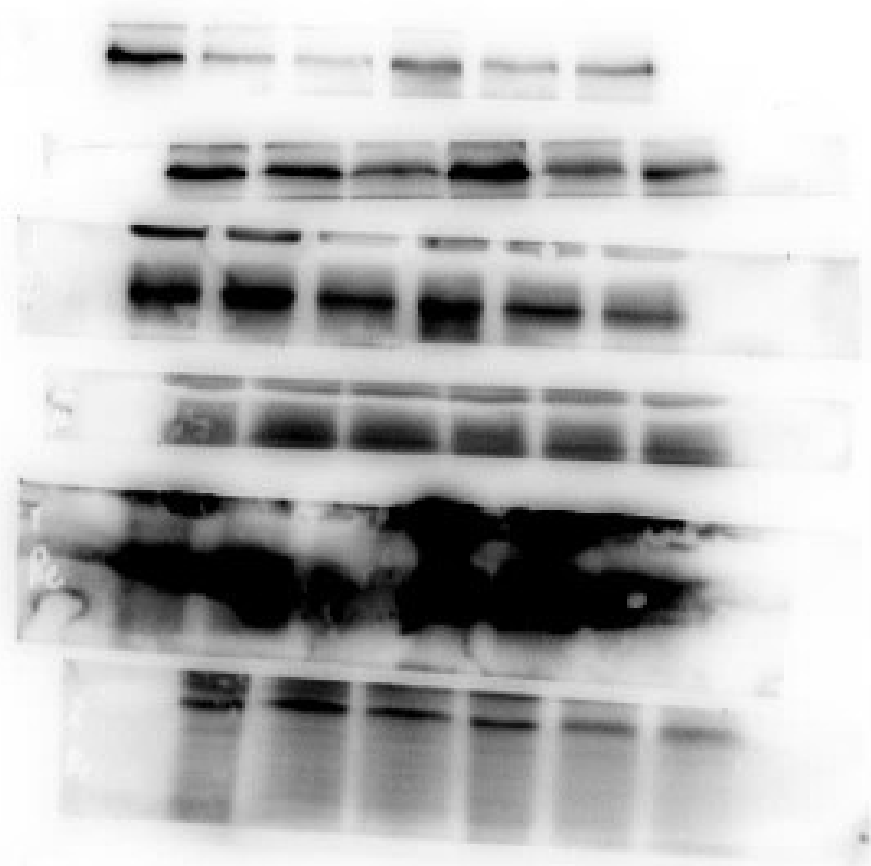

Supplement: Supplementary Materials — Data are available in the supplement file. [file 3648175.f1.zip › 3648175.f1/Figure 8 WB image (1).pdf]
